# *pan-Draft*: automated reconstruction of species-representative metabolic models from multiple genomes

**DOI:** 10.1186/s13059-024-03425-1

**Published:** 2024-10-25

**Authors:** Nicola De Bernardini, Guido Zampieri, Stefano Campanaro, Johannes Zimmermann, Silvio Waschina, Laura Treu

**Affiliations:** 1 Department of Biology, University of Padova, Via U. Bassi 58/B, Padua, 35121 Italy; 2 Evolutionary Ecology and Genetics, Zoological Institute, Kiel University, Kiel, 24118 Germany; 3 Department of Human Nutrition and Food Science, Kiel University, Heinrich-Hecht-Platz 10, Kiel, 24118 Germany; 4 Antibiotic Resistance Group, Max Planck Institute for Evolutionary Biology, Ploen, 24306 Germany

**Keywords:** Genome-scale metabolic model, Metagenome-assembled genome, Metagenomics, Species-level models, Metabolic network gapfilling, Human gut microbiome

## Abstract

The accurate reconstruction of genome-scale metabolic models (GEMs) for unculturable species poses challenges due to the incomplete and fragmented genetic information typical of metagenome-assembled genomes (MAGs). While existing tools leverage sequence homology from single genomes, this study introduces *pan-Draft*, a pan-reactome-based approach exploiting recurrent genetic evidence to determine the solid core structure of species-level GEMs. By comparing MAGs clustered at the species-level, *pan-Draft* addresses the issues due to the incompleteness and contamination of individual genomes, providing high-quality draft models and an accessory reactions catalog supporting the gapfilling step. This approach will improve our comprehension of metabolic functions of uncultured species.

**Supplementary Information:**

The online version contains supplementary material available at 10.1186/s13059-024-03425-1.

## Background

The automatic generation of genome-scale metabolic models (GEMs) for uncultured species is a powerful approach for exploring microbially mediated processes. Through GEM application, recent studies have solved many open questions that were previously impossible to tackle. For instance, they disclosed microbial dynamics based on amino acid auxotrophies in a complex microbiome [1], identified disease-specific marker molecules in the human gut [2], and proposed ecological roles and industrial applications of species that cannot be isolated [3–5]. The approach adopted for GEM reconstruction by some of the most successful tools, such as *CarveMe*, *gapse*q, *RAVEN*, *Merlin*, *AuReMe*, and *ModelSEED*, is based on sequence homology search and the definition of gene-protein-reaction rules [6–11]. This strategy defines an organism’s reactome, delineating the non-redundant set of metabolic reactions governing cellular processes [12]. Unfortunately, while significant strides in automated de novo reconstruction of metabolic models from metagenome-assembled genomes (MAGs) have been made, inherent limitations have also been faced. Challenges stem from the complex nature of the explored microbiota, encompassing diverse species with varying metabolic capabilities. Additionally, the absence of comprehensive information on gene functions and regulatory systems, coupled with the difficulty of inferring metabolic networks from incomplete genome sequences, further complicates the process [3, 13, 14]. This latter issue is of particular concern when dealing with MAGs, which are inherently prone to incompleteness and contamination. Biases affecting the quality of a MAG derive from limits in the assembly and binning procedures, which often cause the generation of metagenome collections with many low-quality features [15]. Consequently, developing a precise GEM reconstruction strategy from MAGs requires innovative frameworks that go beyond the annotation of single genomes and can take advantage of additional contextual information. Examples of relevant modeling approaches include gapfilling methods that limit the reaction universe based on taxonomic information and topology-based gapfilling methods that leverage high-order network properties to train machine learning models and resolve non-trivial inconsistencies [16, 17]. By drawing relevant information from taxonomically or topologically related organisms, gaps arising from MAG incompleteness can be resolved.

Neglecting the relevance of MAG completeness level in GEM reconstruction may result in erroneous prediction of metabolic capabilities. For instance, misinterpreting an artifactual deficit of functions as a genuine biological signal could occur when considering modules in the “nucleotide metabolism” and “biosynthesis of other secondary metabolites” domains, which have been found to be most affected by genome incompleteness [18]. In general, GEMs derived from low-quality MAGs have limited ability in representing reliable metabolic capabilities [19]. Additionally, although there is a positive correlation between MAG completeness and the structural integrity of MAG-derived metabolic models [6], highly complete MAGs may fail to capture core genes [20]. As a consequence, there is a pressing need for novel reconstruction approaches that consider biases induced by the technical limitations of the metagenomic approach.

A common way around MAG incompleteness relies on mapping the taxa under investigation to the closest high-quality genome [19, 21]. For instance, a nucleotide similarity search on databases may allow the retrieval of genomes associated to the same species-level genome bin (SGB), which is a collection of sequences binned together based on their similarity and assumed to represent an individual microbial species. However, since a large fraction of the species living in the natural environment are yet to be isolated, many SGBs include only metagenomic sequences [22, 23]. As a result, for these species similarity search can, at best, identify MAGs of high quality according to MIMAG standards [24]. Thus, in these cases the retrieved best MAGs within a SGB remain susceptible to incompleteness, thereby limiting the interpretability in related analysis.

In recent years, multiple efforts have tried to organize extensive MAG collections summarizing species-level variability across diverse microbiomes [25–28]. To this end, closely related taxa can be separated by applying a 95% average nucleotide identity (ANI) threshold, as the discriminatory boundary for species-level genome clustering [29]. Previous studies have exploited these collections to improve the quality of GEMs derived from individual MAGs. Specifically, inter-species or -strain reactome analyses were performed adopting a genomic comparison approach to derive refined strain-level models starting from a reference [30, 31]. Multiple examples show the successful implementation of this homology gene-driven approach on prokaryotes and eukaryotes of biotechnological and medical interest [30–32]. For example, a model of the opportunistic human fungal pathogen *Aspergillus fumigatus* was developed from 252 isolates and MAGs to understand the metabolic background of infection versatility [33]. Unfortunately, the application of this approach is hardly scalable to large datasets due to the need for complete reference genomes and extended manual curation. Recently, new tools were developed to use microbial genomes in order to reconstruct species-level pan-models independently from manual curation [34, 35], such as the “createPanModels” software from the Microbiome Modeling Toolbox 2.0, which is capable of reconstructing pan-models at different taxonomic levels. Unfortunately, this tool is limited to the strains present in the AGORA collection, which have complete whole-genome sequences [36]. A second software, MIGRENE Toolbox, uses a reference-based model approach starting from a generalized gut microbial model to create a reaction profile for each species. Based on gene presence/absence and using the generalized models as a baseline, MIGRENE is able to reconstruct species-level GEMs starting from microbial pan-genomes [35]. The application of these tools is however challenging when applied in contexts other than the human gut microbiome.

As a more flexible alternative to previous studies, the current work introduces *pan-Draft*, a novel tool integrated into the *gapseq* pipeline for the automated reconstruction of species-level GEMs (*pan*-GEMs). The proposed method leverages the redundancy present in large MAG collections to mitigate the lack of genetic information frequently associated with single MAGs. Specifically, through pan-reactome analysis the frequency of non-redundant metabolic reactions encoded in the genomes of a SGB can be quantified, thereby effectively characterizing the whole enzymatic capacity of a group of sequences. Importantly, this approach operates independently of reference metabolic models or genomes, making it applicable to species clusters for which prior metabolic knowledge is either limited or completely absent. Cross-comparison of draft models belonging to the same taxon facilitates the reconstruction of a network representative of the majority of the metabolic variability within the cluster. Additionally, this approach enhances the gapfilling curation step by summarizing species-level metabolic diversity into a catalog of accessory reactions.

## Results

### Implementation of *pan-Draft* and dataset structure

The *pan-Draft* module is integrated within *gapseq*, a bioinformatics pipeline designed for the automated de novo reconstruction of ready-to-use GEMs. Given a genomic sequence, the pipeline uses two modules (i.e., *find* and *find-transport*) to perform a sequence similarity search against databases collecting gene-protein-reaction annotation rules and a third module (i.e., *draft*) to reconstruct a preliminary metabolic network [6]. By processing the output of the *find*, *draft*, and *find-transport* commands, *pan*-Draft aims to reconstruct high-quality draft metabolic models from several incomplete MAGs having a potentially complementary gene content through a comparative pan-reactome analysis. Briefly, a network analysis is performed using a minimum reaction frequency (MRF) threshold to generate a species-level draft model. Simultaneously, reaction weights, used in the gapfilling step, are defined depending on respective reaction frequencies (further details in the Methods section). The method is applicable to any set of prokaryotic genomes, whether they include isolates or MAGs only. However, its primary relevance is conceived for situations where standard genome-centric metagenomics struggles to accurately recover high-quality MAGs for any species of interest. The computational cost of *pan-Draft* is limited to a few minutes, making it easily applicable to pre-existing GEM collections (Additional file 1: Fig. S1). Nevertheless, the reconstruction of large GEM catalogs remains a demanding process, which can limit the use of this method on personal computers for sequence collections including hundreds of MAGs (Additional file 1: Table S1).

In order to identify the target studies that can benefit from species-level model integration methods and to validate the approach, two among the largest available MAG datasets deriving from highly different environments were used. The first is the Unified Human Gastrointestinal Genome catalog (UHGG v.2.0.1), which collects 289,232 genomes clustered into 4744 SGBs. The UHGG dataset represents a host-associated microbial domain populating either a strictly anaerobic or microaerobic environment [25]. The second dataset, the Ocean Microbiomics Database (OMD, v 1.1), collects 34,815 genomes clustered into 8308 SGBs [26]. This collection, in comparison to the former, includes a more complex and less explored microbiome spanning a wider spectrum of environmental conditions and ecological heterogeneity. Despite this variability, the majority of its metagenomes (approximately 58%) were sampled from the epipelagic zone, reflecting microbiomes adapted to aerobic conditions. With the goal of exploiting the redundancy of multiple MAGs, we counted the number of SGBs with a minimum of 30 associated MAGs clustered at 95% ANI in either dataset, resulting in 450 and 135 SGBs, respectively. Notably, the majority of these SGBs lack an isolated representative (see columns 5–6 in Table 1), highlighting both the gap existing between cultured and uncultured microorganisms and the exploitable genomic redundancy for uncultured taxa. Specifically, 375 and 126 SGBs had no isolated representative in the UHGG and the OMD catalogs (Table 1).

**Table 1:** Summary of the OMD and UHGG catalog characteristics. A minimum of one and ten isolate/s were used to filter SGBs with (w/) reference genome/s in the OMD and UHGG catalogs, respectively. In each cell is reported the number of SGBs and the corresponding number of associated genomes. The set of SGBs used in this work is highlighted by an asterisk

**Dataset**	**Total genomes**	**Total SGBs**		**w/ non-redundant sequences, and reference genome/s**	**w/o reference genomes **	**w/o reference genomes, and best MAG completeness level <90% **
UHGG	289,232	4744	≥30 MAGs	75* 66345	375 56782	28 3065
			≥20 MAGs	75 66345	496 59695	41 3373
			≥10 MAGs	78 66453	802 63847	76 3832
OMD	34,815	8308	≥30 MAGs	9* 488	126 6395	59 3089
			≥20 MAGs	14 611	249 9317	116 4456
			≥10 MAGs	25 777	611 14181	296 6909

**Fig. 1:**
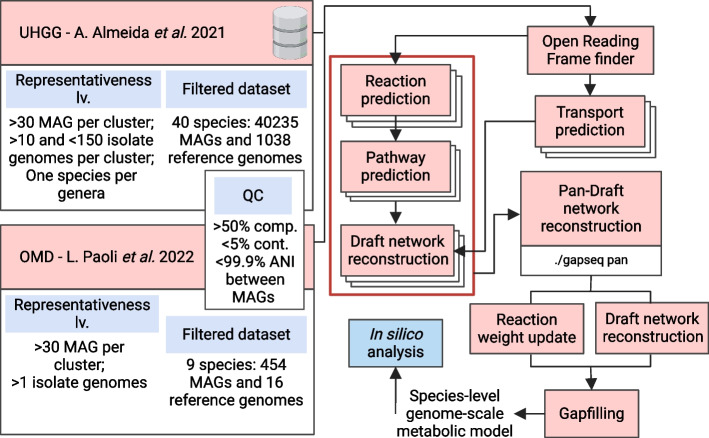
Flowchart showing the dataset filtering steps and the *pan-Draft* workflow. Set of filtering criteria implemented on the UHGG and OMD dataset to select SGBs with non-redundant genomic information for *pan-Draft* validation. A minimum threshold of 30 MAGs was imposed to reconstruct a set of draft models through *gapseq*. *pan-Draft* is integrated within the reconstruction pipeline by processing the draft models and the genetic evidence of reactions. The information used for each genome of a SGB to generate the *pan*-GEM is highlighted by the red box

The SGBs having at least one available reference genome allowed us to validate the *pan*-GEM reconstruction method. To do this, the databases underwent filtering to select non-redundant genomes on the basis of a 99.9% ANI and to exclude those originating simultaneously from the same species and sample [25]. Afterwards, SGBs with a minimum of 30 MAGs, at least one reference genome in the OMD, and at least ten reference genomes in the UHGG (Fig. 1) were further selected. The filtering resulted in 75 SGBs including 62,034 MAGs and 4311 reference genomes for the UHGG database, and nine SGBs having 472 MAGs and 16 references for the OMD catalog (Additional file 2: Table S1 and Table S2). Both datasets included only MAGs with a completeness level above 50% and contamination below 5%.

### Role of genome completeness in model reconstruction: the foundation for a “*pan*” perspective

**Fig. 2:**
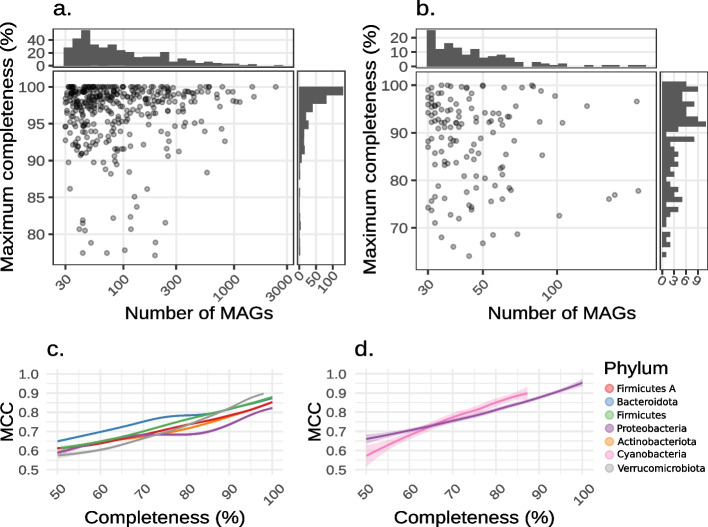
Potential improvement of the species-level metabolic model reconstruction approach. Completeness level of the representative MAGs for SGBs with no reference genome and at least 30 MAGs in the corresponding database (**a**, **b**). On the lower panels, a generalized additive model was fitted to the similarity measure of the gapfilled models reconstructed for 84 SGBs with varying completeness level compared with the species-representative reactome gold standard (**c**, **d**). Data regarding the UHGG and the OMD databases are presented in panels **a**, **c** and **b**, **d**, respectively

To define a baseline for *pan*-GEM reconstruction, we first analyzed the relationship between MAG completeness and draft GEM quality in the two datasets. The selected MAGs covered a wide spectrum of completeness in both environments, ranging from 50 to 100%. The maximal completeness of MAGs for individual SGBs lacking cultured representatives also showed significant variability. In several cases, these MAGs did not reach 90% of completeness (Fig. 2a, b). Specifically, OMD had 59 SGBs (0.7% of the total) with maximal MAG completeness levels below 90%, whereas UHGG had 28 (0.6% of the total—Table 1). Traditional de novo draft GEM reconstruction is expected to yield highly gapped models in these cases. To assess the extent of these gaps, we computed the correlation between the MAG completeness level and the quality of the reconstructed GEMs. The latter was quantified by comparing the presence and absence of reactions shared between GEMs reconstructed from individual MAGs (MAG-GEMs) and GEMs of reference genomes (iso-GEMs). This similarity was expressed as the Matthews correlation coefficient (MCC). By fitting a generalized additive model to the data (Fig. 2c, d) a strong and statistically significant positive correlation emerged (Spearman correlation, *rho* = 0.78, *P*-value < 2.2e − 16) between the genome completeness and the MCC, confirming the need of a method to overcome current completeness limits. Interestingly, significant variability in this relationship was also identified across bacterial phyla (Fig. 2c, Additional File 1: Fig. S2).

### Selection of species *pan*-reactome

Generating species-level metabolic models could require combining draft GEMs reconstructed from contaminated MAGs. Consequently, a strategy is needed to exclude exogenous reactions from the pool of endogenous SGB-specific enzymes. Moreover, correctly predicted reactions (i.e., those actually belonging to a species pan-reactome) that are present only within small sub-populations should be preferentially filtered out to generate a draft representative of a species metabolism. To achieve this, we defined a MRF threshold necessary to discriminate whether a reaction should be directly included in the draft model or not and empirically determined its optimal value. Specifically, the draft *pan*-GEM for each species was reconstructed by selecting all available MAGs and varying the MRF parameter from 0 to 100%. The *pan*-GEM quality was once again quantified by comparing the model reaction set with the species reactome gold standard.

**Fig. 3:**
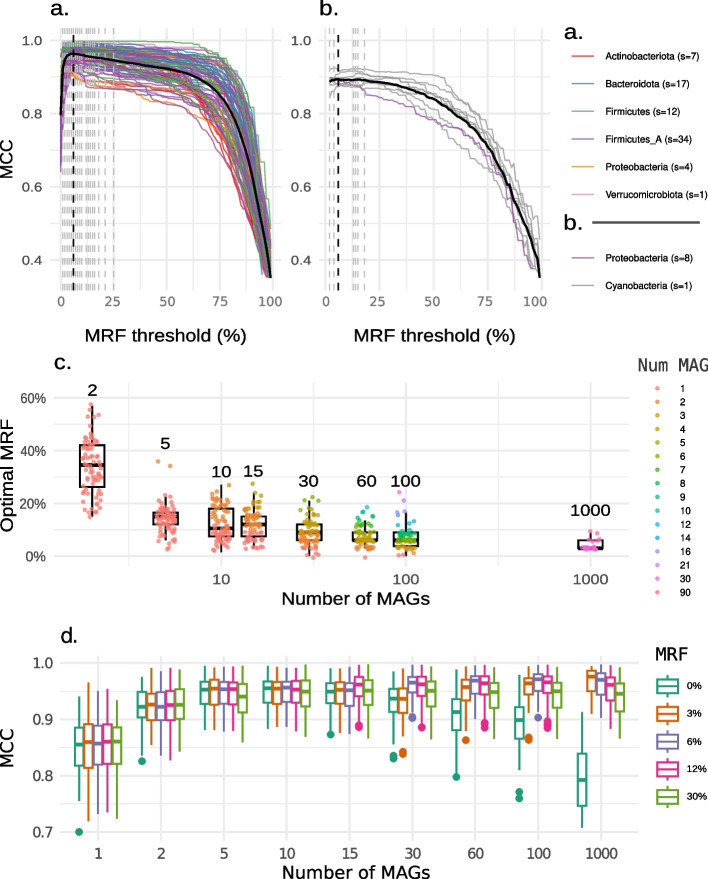
Minimum reaction frequency threshold definition for draft *pan*-GEM reconstruction. Quality of the models generated at the species level with a selected MRF ranging from 0 to 100%. The gray and dark vertical dashed lines highlight the MRF maximizing the MCC for each SGB and across all the species, respectively. The black solid line depicts the average MCC across species in the UHGG (**a**) and the OMD (**b**) dataset. Boxplots show the relationship between the optimal MRF and the number of MAGs used to reconstruct the draft *pan*-GEM (**c**), as well as the effect of varying MRF on the quality of the *pan*-GEMs (**d**). Both boxplots show the distribution across all analyzed SGBs. The results are the median (**c**) and average (**d**) of 10 iterations per SGB with a random selection of the initial MAGs. The colors in **c** show the median number of MAGs in each SGB corresponding to the selected MRF threshold, computed as the number of MAGs in the subset times the MRF

Results highlighted a certain degree of variability in optimal MRF thresholds between the UHGG and OMD datasets and across SGBs (Fig. 3a, b). Nevertheless, in both cases, a threshold above 20% tends to underestimate the size of the SGB reactome, as indicated by the steep decrease in MCC for MRF thresholds between 20 and 100% in all the species. Below that value, the MCC curves flatten, arguably due to the simultaneous effect of contaminant reactions and reactions associated with accessory metabolism present at different frequencies in the species sub-populations. As a trade-off, the optimal MRF value was thus obtained by averaging the MCC across all the species in any of the two datasets and selecting the threshold maximizing this metric. The optimal MRF threshold was thus equal to 6% for both the UHGG and the OMD catalogs (Fig. 3a, b). Overall, these results suggest that a threshold within the range of 5–10% allows the reconstruction of solid *pan*-GEMs for SGBs across different and distant taxonomic groups.

Further validation of the identified optimal threshold was performed by testing MRFs ranging from 0 to 100% on MAG subsamples of variable size. Specifically, MAGs were randomly sampled to form batches of *n* = 2, 5, 10, 15, 30, 60, 100, and 1000 genomes, iterating ten times for each SGB, and the obtained MCCs were averaged over the iterations for a robust characterization. The results confirmed that the optimal MRF lies between 5 and 10% for batches including tens to one thousand MAGs and suggested that it roughly approaches 1/n with a decreasing batch size below 15 MAGs (Fig. 3c). This was in part expected given that, with few MAGs, unique differences between genomes become proportionally more frequent and, at the same time, contaminant and accessory reactions get less distinguishable based on their frequency. With *n* MAGs, these reactions typically have a frequency of 1/*n* and the mean optimal MRF lies just below this value, essentially including all the reactions in at least one MAG-GEM (Fig. 3c). As a result, most *pan*-GEMs reconstructed from fewer than 10 MAGs incorporate the union of all reactions in the associated MAG-GEMs. In contrast, when the number of available MAGs is 15 or above, a low MRF threshold is essential to filter out (rarer) contaminant reactions and retain accessory ones.

Such a transition can be further appreciated when observing the effect of five different MRF thresholds on the quality of *pan*-GEMs reconstructed with an increasing number of MAGs (Fig. 3d). The results clearly show a drastic model improvement using few MAGs (2–5) as compared to a single MAG, with the MCC nearing its maximum at 30 MAGs and showing marginal improvements using larger batches. In the extreme case of employing a 0% threshold—i.e., including all the reactions present in any available MAG—the performance is the best achievable one at low MAG numbers, whereas it yields a MCC drop of 0.02 ± 0.02 using 30 MAGs and 0.17 ± 0.06 using 1000 MAGs. Thus, while this permissive approach can be safely used when having genome sequences from isolated microorganisms [34, 36], it would lead to a false positive increase of 65.78 ± 41.11 and 424.73 ± 147.63 reactions with 30 and 1000 MAGs, respectively.

In the following analyses, a 6% MRF threshold was set as standard to reconstruct the species-level GEMs. Based on the hypothesis that the contaminating genetic material present in a MAG is not redundant across the species, this threshold provides a high level of confidence to exclude contaminating reactions when dealing with lowly contaminated (estimated level < 5%) MAGs.

### Properties of species-level metabolic models

**Fig. 4:**
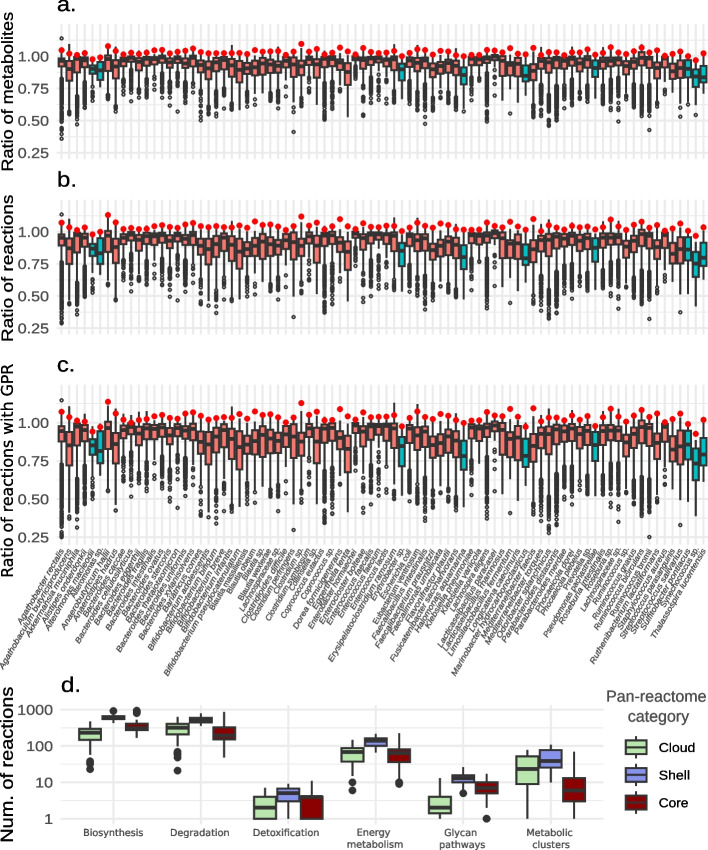
Structural and metabolic features of *pan*-GEMs. The upper boxplots summarize the ratios of the number of metabolites (**a**), the number of reactions (**b**), and the number of reactions with associated genetic information (**c**) in MAG-GEMs and *pan*-GEMs, relative to the corresponding median values of iso-GEMs. Red dots indicate the statistics for the *pan*-GEMs reconstructed with all the MAGs of an SGB, either in the UHGG (red boxes) or OMD dataset (light blue boxes). The lower boxplot shows the functional classification of reactions grouped into pan-reactome categories by MetaCyc pathways ontology (**d**). Core, shell, and cloud consist of all reactions with a frequency higher than 95%, between 5 and 95%, and below 5%, respectively, in all the GEMs of any SGB

Having established an optimal pan-reactome selection criterion, we set out to characterize the generated *pan*-GEMs. On average, they possessed 3.1 ± 2.1% more metabolites and 4.0 ± 2.7% more reactions than their counterparts obtained from reference genomes (Fig. 4a–b). Thus, as also visible in Fig. 3, they largely reflected the corresponding iso-GEMs in terms of metabolic network components. In contrast, MAG-based GEMs of origin contained up to 64% fewer metabolites and 75% fewer reactions.

The most represented functional super-groups of metabolic reactions pertained to biosynthesis and degradation (Fig. 4d). In particular, core metabolism had numerous reactions for the biosynthesis of lipids, nucleotides, secondary metabolites, and cofactors and for the degradation of carbohydrates (Additional file 1: Fig. S3). Both amino acid biosynthesis and degradation were highly represented pathways. Most of these biomolecules are involved in primary metabolism and have been previously linked with core genome functions by pan-genome analysis [37]. Shell and cloud reactions generally followed the same trends of core reactions, with some exceptions where cloud reactions were over-represented as compared to the core: biosynthesis of storage compounds and polyamines and degradation of fatty acids and lipids, aromatic compounds, and amino acids.

### Species-level metabolic models show improved network structure

**Fig. 5:**
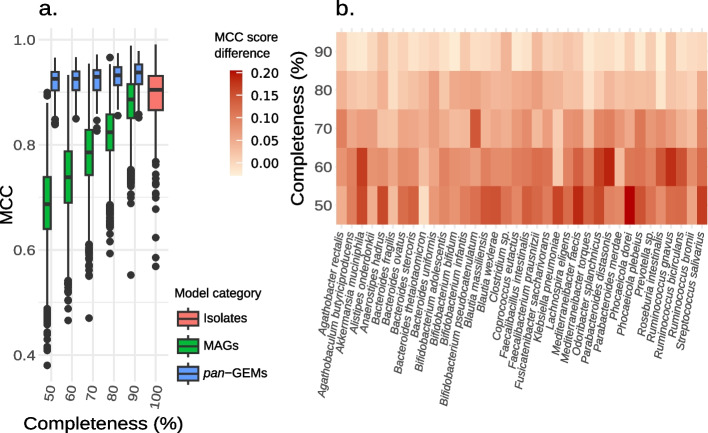
Structural quality improvement obtained for the *pan*-GEMs in comparison to the MAG models. Graphs showing the changes in quality between the *pan*-GEM and MAGs’ models in relation to the reference reaction set. Global results are reported for all SGBs except *E. coli* (**a**) and for single species (**b**). The heatmap reports explicitly the average difference between the MCC of each species *pan*-GEM and of the GEM from its most complete MAG. Species-level model improvements were analyzed based on MAG completeness. Completeness level values highlight the thresholds used to subset the MAGs into five groups. Only SGB with more than 30 MAGs in each subset were depicted. All statistics are calculated on gapfilled models

To assess the extent of improvement in the quality of a *pan*-GEM compared to the individual GEMs of the same species, a second set of model reconstructions was generated. MAGs were divided into five sets based on equally spaced intervals over their completeness range and, for each SGB, 30 MAGs were randomly sampled for 10 times within every available completeness interval. Those MAG sets were then used for MAG-GEM and *pan*-GEM reconstruction while recording model quality statistics for each set (Fig. 5). In this way, multiple *pan*-GEMs were generated for every SGB, each time simulating the discovery of a different MAG set in a predefined completeness interval. As a result, the variability in model quality was found to be highly dependent on genome completeness levels. More specifically, the variability of the MCC calculated for GEMs obtained considering all the species under investigation tends to decrease as completeness increases (Fig. 5a). This was partially expected since pathway inference and gapfilling are susceptible to the specific combination of genes identified in an individual MAG. Conversely, the quality of *pan*-GEMs generated iteratively using 30 random MAGs associated with the same SGB of the same cluster of completeness had a strongly reduced variability and an enhanced structure. The MCC had a steadily increasing value, with the maximum associated with the MAGs belonging to the best cluster (90–100% completeness). Most notably, the highest improvement during *pan*-GEM generation was obtained with highly incomplete genomes. Therefore, the improvement rate was inversely dependent on the completeness of the MAGs utilized. Interestingly, the quality of *pan*-GEMs was comparable with that of the corresponding iso-GEMs, even when starting from low-quality MAGs with completeness levels between 50 and 60% (Fig. 5b). This observation held true for 74 SGBs, but not for *Escherichia coli*, which was processed independently because the average quality of its iso-GEMs was predicted to be lower compared to the iso-GEMs of the other species (see Additional file 1: 2.4 Linking *Escherichia coli* genomic variability to model quality: insights from pan-reactome content [38, 39]). Specifically, the *pan*-GEMs showed a slight overestimation of the reaction content of individual iso-GEMs. This overestimation was attributed to the species’ wide genetic variability and the large number of available isolates (Additional file 1: Fig. S4). Despite this difference, the result for all species clearly highlights the potential of the proposed approach to generate highly accurate species-level GEMs from fragmented MAGs with a low completeness level (Fig. 5b, Additional file 1: Fig. S5).

At a finer scale, similar patterns can be observed across the 75 SGBs having at least 30 MAGs per completeness interval (Fig. 5b, Additional file 1: Fig. S6). The results showed that the vast majority of *pan*-GEMs exhibited larger model quality improvements at lower MAG completeness, thus suggesting that *pan-Draft* is suitable across taxonomic groups. Certain species, such as those belonging to the genus *Ruminococcus*,* Streptococcus*, and *Faecalibacillus*, demonstrated a pronounced progressive gradient of improvement rate across the various completeness levels. Moreover, all the species showed positive improvement for highly complete MAGs, which despite being minor are worth considering. Overall, these results highlight the improvement brought by *pan*-GEMs as compared to the models obtained from the most complete MAGs, i.e., those that would be most likely selected in a typical metagenome modeling study.

### Species-level metabolic models show higher performance in predicting fermentation by-products

**Fig. 6:**
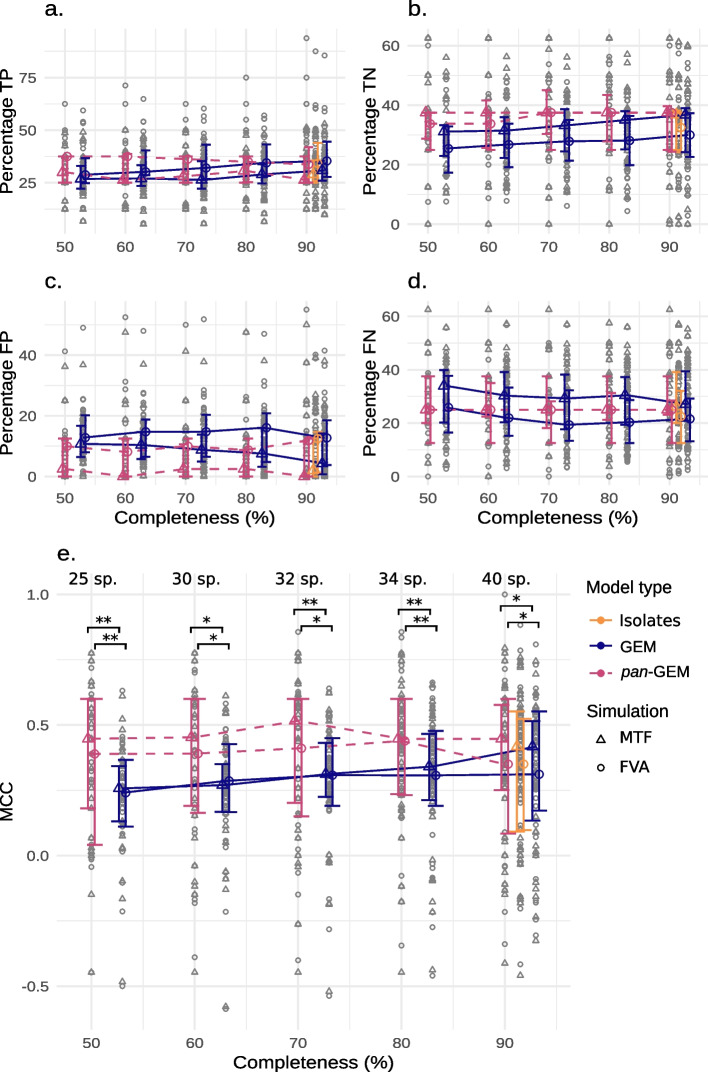
Improvement in fermentation product prediction by the *pan*-GEMs in comparison to MAG- and iso-GEMs. Results of the fermentation product test under anaerobic growth obtained with FVA (circles) and MTF flux balance analysis (triangular). Data summarized at the species level (gray symbols) show in percentage the number of TP (**a**), TN (**b**), FP (**c**), and FN (**d**) obtained across all genomes within a species. Similarly, the overall performance is summarized with the MCC (**e**). Colored dots and error bars show the median, 1st, and 3.^rd^ quartile of all species within the subset obtained according to the MAG completeness level. The number of bacterial organisms tested for each completeness level subset is reported above the box group (n. sp.). Statistically significant differences in the MCC distribution, as determined by a paired t-test, are indicated as follows: * *P* < 0.05, ** *P* < 0.01

Next, we sought to test whether the obtained *pan*-GEMs could more accurately reproduce fermentation capabilities of gut microbial species compared to traditional MAG- and iso-GEMs. The fermentative potential for a given compound was defined as the capacity of a species to secrete that compound over a pre-defined flux threshold normalized over its growth rate (details in the “Methods” section). To assess this, a comparative analysis of the predictive accuracy of in silico simulations was conducted for eight common anaerobic fermentation products using metabolic model reconstructions for 41 different species of the UHGG catalog (Fig. 6). Validation was performed on selected organisms having: (i) at least a set of *pan*-GEMs among the 75 SGBs previously reconstructed, (ii) fermentation products experimentally described in literature or in the NJC19 dataset (Additional File 2: Table S3) [40]. The selected metabolites include common anaerobic fermentation end-products, such as acetate, butyrate, lactate, and ethanol. The evaluation involved quantifying the percentage of correctly and incorrectly estimated end-products based on the total number of predictions performed for all the models of a species (Additional File 2: Table S5). Specifically, the confusion matrix per SGB was determined by summing up all the predictions belonging to the same category (e.g. true positive (TP), true negative (TN), false positive (FP), or false negative (FN)). Afterwards, the average percentage of TP and TN metabolites across species was estimated through the normalization of the confusion matrix of each SGB by the total number of tests in that group (i.e., = num. GEMs * num. tested compounds; Additional file 2: Table S6). Also in this case, to investigate the influence of the MAG completeness level on the prediction performance, the species-level models were divided into five sets with increasing source MAG quality. According to the minimize-total-flux (MTF) analysis and flux variability analysis (FVA), the *pan*-GEMs consistently outperformed the GEMs of individual MAGs with completeness levels between 50 and 90% (Fig. 6a, b). In comparison to high-quality MAG-GEMs (i.e. completeness levels > 90%), the number of correct positive predictions by *pan*-GEMs showed a small decline, but the values remained comparable to those of iso-GEMs. A more detailed analysis revealed that models of highly incomplete MAGs (completeness level between 50 and 60%) exhibited a high rate of FP (Additional File 1: Fig. S7), whereas corresponding *pan*-GEMs tended to reduce the number of scattered FPand consolidate the correct predictions. Indeed, the variability of predictions is reduced because a high percentage of *pan*-GEMs converge to the same by-product. Statistically significant differences in MCC were observed for all MTF and FVA simulations (paired *t*-test, *P* < 0.05—Additional File 2: Table S4). On the contrary, the differences were not significant for iso-GEMs compared to the other two groups of models sourced by genomes with completeness above 90%.

Finally, a comparison against GEMs from the MIGRENE collection was performed to determine whether *pan*-GEMs reached state-of-the-art performances. This test involved *pan*-GEMs from 36 SGBs with species names matching those in the MIGRENE collection and assessing the same fermentation products as before (Additional File 2: Table S3). *pan*-GEMs demonstrated superior performance, showing consistently more correct positive and negative predictions for both FVA and MTF analysis (Additional File 1: Fig. S8c). Specifically, *pan*-GEMs appeared to predict well the secretion of small molecules such as acetate, butyrate, and ethanol but showed low sensitivity for succinate, propionate, and lactate (Additional File 1: Fig. S8b). In contrast, MIGRENE’s models estimated more TPs for these latter metabolites but produced numerous erroneous estimations for hydrogen and butyrate (Additional File 1: Fig. S8a). Although *pan-Draft* showed lower sensitivity for a subset of by-products in FVA, the net MCC improvement was 0.20 and 0.18 for FVA and MTF, respectively. Overall, these results highlight the capacity of *pan-Draft* to enhance the prediction accuracy of the main fermentation products of representative bacteria of the human gut microbiome during anaerobic growth.

## Discussion

To date, cultured microbial species represent only a small fraction of global microbial diversity, as demonstrated by an extensive comparison of 3170 metagenomes with the NCBI collection of known reference genomes (RefSeq release 93; 151,730 isolates) [27]. Here, on average only ~ 14.6% of reads per sample could be aligned to the references [27]. Thus, in silico metabolic network analysis of most microbial species, just like genomic analysis, has to rely on MAGs, with the associated risks of potentially missing relevant functional components. For this reason, the present study introduced *pan-Draft*, a new tool that integrates an automatic genome-scale model reconstruction pipeline and provides a framework to obtain a holistic perspective on both the potential and variability of unculturable species metabolism. The novelty of *pan-Draft* lies in the combination of a homology-based approach, predicting the metabolic potential of a genome, with a pan-reactome analysis assessing which reactions are common constituents of the metabolism of a taxon. Other software motivated by similar rationales, such as the MIGRENE toolbox, are currently in development [35]. Compared to this tool, which implements a species pan-genome-based reconstruction approach, *pan-Draft* leverages a pan-reactome approach that preserves the gene scores obtained through homology search and allows a more informed gapfilling of a species’ reaction set. Moreover, *pan-Draft* does not require context-specific reference models or an extended gene catalog and leverages gene-protein-reaction annotation rules of multiple datasets (i.e., MetaCyc [41], KEGG [42], and ModelSEED [43]). This allows an easier application of metabolic network reconstructions outside the scope of highly studied environments such as the human gut microbiome.

To evaluate the models generated with *pan-Draft*, large publicly available databases were screened to identify SGBs containing both MAGs and high-quality reference genomes from species grown in pure culture. The structural quality of the species-level models was assessed by comparing obtained species-level reactomes with the reaction set from reference genomes. The results suggest that *pan-Draft* generates accurate network reconstructions by filling gaps in a species’ metabolic functions encoded in a single genome. For example, it has been observed that ribosomal protein genes could be missing in more than 20–40% of near-complete MAGs [44]. Therefore, while these essential genes are often absent, others may share a similar fate in MAGs, thus highlighting the importance of pan-reactome-based reconstructions. Nevertheless, one of the most interesting aspects of GEMs lies in theirpotential to accurately predict a species’ functional role in the environment, such as providing insights into secreted compounds and the consequent microbial ecosystem dynamics. Therefore, the functional quality of *pan*-GEMs was evaluated here by considering the production of common fermentation metabolites across MAGs with various completeness levels.

### The crucial need for redundant metagenomic collections

The validation of *pan-Draft* on distantly related species demonstrated that the model reconstruction method is solid with respect to the microbial phylogenetic origin. Using the suggested MRF threshold, a *pan*-GEM can be reconstructed for any species of interest. However, the potential of *pan-Draft* grows along with the level of MAG incompleteness (Fig. 5a), thus the tool is especially relevant for uncultivated species difficult to characterize with standard shotgun-based metagenomic approaches. Challenges in obtaining high-quality MAGs stem from various factors, such as the microbiota complex structure, the high strain heterogeneity, and the abundance of target species relative to the overall community [20, 45]. In the chosen databases, a considerable number of species potentially affected by these issues was found. Specifically, the maximum completeness level of MAGs never exceeds 90% in 28 frequently retrieved species in the UHGG database (e.g. members of the genera *Angelakisella*), and in 59 of the OMD catalog (Fig. 2a, b). Despite having only about 1/8th of the genomes present in the UHGG, the OMD dataset hosts a greater number of species suitable for significant model enhancement using *pan-Draft*. This suggests that complex and under-investigated microbiomes, such as those of ocean or soil, are the most interesting targets for the *pan-Draft* usage. Moreover, this study demonstrated that the application of *pan*-*Draft* could be extended to all the 375 and 126 frequently retrieved SGBs present in UHGG and OMD catalogs that are represented by highly complete MAGs. This extension is justified by the consistent improvement in *pan*-GEM quality observed for both low-quality and high-quality MAGs (> 90% completeness, Fig. 5). In addition, this work confirmed the previously detected positive correlation between the genome completeness and the GEM structural quality (Fig. 2c, d). This correlation, initially observed using only the representative MAG of 127 SGBs, highlighted how the GEMs of MAGs with completeness between 90 and 100% register quantifiable improvements [6]. Further supporting evidence comes from a previous comparison of MAGs and isolated genomes of *E. coli* from the same community [20]. This verified that on average, despite the high completeness (~ 95%), MAGs correctly captured only 77% of the core genes and 50% of the variable genes within the population. Moreover, analyzing the GTDB database to understand how genome completeness influences KEGG metabolic pathway fullness, a variation from 70 to 100% in completeness level was found to correspond with a constant increase in module fullness (15 ± 10%) [18]. In summary, focusing on retrieving a species’ highest quality MAG from publicly available databases, as well as adopting methods for expanding MAG annotation [46], helps to minimize issues in GEM reconstruction; however, it does not guarantee to capture the complete functional signal of a species. To overcome this limit, a more comprehensive set of genes can be independently recovered in different MAGs, thus *pan-Draft* can be an essential aid for not-yet-cultured microbes.

### Relevant parameters for species-level model reconstructions

To correctly infer inter-species metabolic interactions using in silico microbial community simulations, it is essential that the individual building blocks (i.e., the single GEMs) accurately predict the species’ requirement and the production of metabolites. The reliability of these predictions depends on whether the reconstruction and curation (e.g. gapfilling) of the model correctly capture the species metabolism [13].

In this study, *pan-Draft* was proven to successfully enhance both the structural quality and prediction accuracy of GEMs reconstructed at the species level, illustrating the applicability of the tool across diverse prokaryotic taxa*.* The structural enhancement of *pan*-GEMs was observed even when using as few as 2 MAGs, demonstrating the applicability of the approach to species encountered in a relatively limited number of samples in the environmental microbiota of interest. An example of a broadly relevant dataset including 536 potential targets (SGBs with no reference genomes) is the Genomes from Earth’s Microbiomes catalog [27]. However, there are also examples specific to certain contexts, like Limnochordia DTU010, a species not yet isolated found in the Biogas Microbiome database over 30 times and believed to play a significant metabolic role in the process [28, 47]. Moreover, while *pan-Draft* has been introduced for reconstructing species-level models, in principle its applicability may not be limited to low-rank taxonomy. For instance, it could be extended to the genus level by properly curating the selection of input MAGs. Moreover, it is worth noting that the use of a subset of available MAGs was done only for test purposes, while for an accurate representation of a species’ reactome, all available MAGs should be considered. Another important parameter impacting the quality and structure of the resulting species-level models are MRF thresholds other than 6%, which is the suggested default value. This study highlighted a certain degree of variability across the optimal MRF of distinct species, thus indicating that adjusting the threshold within a few percentage points might enhance the reconstruction performance in different datasets. However, determining an optimal MRF value for non-isolated species could pose significant challenges due to the lack of a reference reaction set.

This study also benchmarked the ability of *pan*-GEMs to accurately predict how a species converts nutrients present in a medium into biomass and by-products. Previous studies have already adopted this validation approach to assess the predictive performance of GEMs [6]. The identification test of anaerobic fermentation metabolites in up to 20 *pan*-GEMs demonstrated significant improvements (Fig. 6). Notably, *pan*-GEMs exhibited reduced margin for erroneous prediction as compared to GEMs from MAGs with the same completeness level. This observation suggests that the *pan-Draft* module effectively enhances the structure and the predictive capacity of metabolic models compared to the single-MAG approach.

### Limitations and outlook of the approach

*pan-Draft* suffers from the loss of strain-level resolution when reconstructing the metabolic network of a SGB. If the frequency of genomes associated with a specific strain is low within the MAGs collection, reconstructing the species-level draft model is likely to shade the metabolic capacity of that strain. Indeed, MAG reconstructed from metagenomic samples likely consists of chimeric sequences of multiple strains [48]. To date, state-of-the-art tools are capable of predicting the number of strains in a microbial community or the set of single nucleotide polymorphisms, but they still face challenges in disentangling their genome sequence [49, 50]. Phasing and assembly of strain haplotypes are possible only by integrating long and short reads approaches [51, 52]. Therefore, considering the high complexity of the environmental microbial guilds, it is likely that MAGs in large metagenomic databases, such as those used in this study, represent mixed-strain species. To enhance the resolution of *pan-Draft* models, the approach could exploit single amplified genomes (SAGs) or very long high-quality reads [52]. Recent studies implementing single-cell sequencing of human gut microbiome samples have demonstrated that SAGs can reliably retrieve the genomic information of individual strains [53]. Moreover, SAGs manifest the ideal characteristics for a pan-reactome analysis at a low taxonomic level. They are usually generated in high numbers, share low contamination levels, and are highly fragmented (i.e., their completeness rarely exceeds 50%) [53].

Moreover, future developments of *pan-Draft* could also consider integrating multiple omics, such as metatranscriptomic, into the modeling approach. Genome-centric metatranscriptomics has been shown to contribute to moving from the simple prediction of the functional potential towards the estimation of the actual functional activity [14]. In this context, integrating metatranscriptomic data on top of refined *pan*-GEMs can buffer the strain homogenization effect and other potential issues derived from the integration of erroneous reactions due to contamination. Finally, future studies should explore the impact of contamination on the quality of species-level models, specifically testing its influence on the MRF threshold.

## Conclusions

Given the high economic and time costs associated with the isolation of microbial species and for generating complete and uncontaminated genomes, traditional metagenomic approaches are expected to dominate microbial community analysis for the foreseeable future [54, 55]. This dominance will result in extensive MAG collections containing multiple versions of species’ genomes. In this context, we introduced *pan-Draft*, a tool capable of enhancing our capacity to investigate the metabolism of elusive minorities in microbial communities using scattered genetic information. The primary goal of *pan-Draft* is to generate metabolic models that faithfully represent a species’ potential by addressing challenges associated with binning issues affecting individual MAGs, thereby preventing the loss of relevant parts of the metabolic network. The results of this work indicate that *pan-*GEMs can serve as an improved building block for larger ecosystem-scale models. Furthermore, the tool provides a foundation for versatile pan-reactome analysis at different taxonomic levels and its logic lends itself to being generalized to other GEM reconstruction tools. Therefore, as more metagenomes from understudied environments become available through standardized and centralized resources, the usage of the presented tool is expected to become particularly relevant in the coming years.

## Methods

### Reference genome and metagenome databases

The datasets used to benchmark the approach include the updated version of the Unified Human Gastrointestinal Genome catalog (UHGG v.2.0.1), accessible at http://ftp.ebi.ac.uk/pub/databases/metagenomics/mgnify_genomes/, and the Ocean Microbiomics Database (OMD, v 1.1), available at https://microbiomics.io/ocean/ [25, 26]. These datasets represent two of the most recent and complete representations of the human gut and ocean microbiome, both published in 2022. Additionally, both datasets include MAGs from various biosamples and reference genomes from isolated microbes. Where possible, their curators clustered reference genomes and MAGs belonging to the same species based on 95% ANI. In order to reduce the redundancy of genomes associated with identical samples being processed in multiple studies, preliminary filtering was applied to the dataset (see Almeida et al., 2021 for details) [25]. More specifically, MAGs were initially filtered based on the study and the sample ID. Additionally, we de-replicated conspecific genomes, employing a stringent 99.9% ANI threshold. This procedure was executed using dRep (v.3.4.5) with specific options, namely “-pa 0.999 –SkipSecondary” [56]. The original taxonomic classification based on GTDB-Tk annotation (v0.3.1 for UHGG and v.1.0.2 for OMD) and MAG quality assessment based on CheckM (v.1.0.11 for UHGG and v.1.0.13 for OMD) were kept unchanged [57, 58]. The taxonomic classification includes suffixes added according to the GTDB nomenclature to indicate groups of species that, while not appearing monophyletic in the GTDB reference tree, have other evidence suggesting they might actually be monophyletic, as well as groups whose placement tends to change or be unstable between different releases of the database. Estimation of MAG completeness and contamination through CheckM is possible even without matched reference genomes as this tool uses a broad set of lineage-specific marker genes obtained through the reconstruction of a reference genome tree.

To quantify the number of species that could potentially benefit from the application of the *pan-Draft* module, the datasets were filtered to select species without any matching reference genome and with a minimum of 30 MAGs. On the contrary, to assess the quality of models reconstructed for single MAGs and at the species level, species-specific reference reaction collections were generated by selecting species represented by genomes derived from isolated cultures. The datasets were filtered according to the number of available reference genomes in the two environments. For the OMD dataset, species were included in the analyses if they had at least one reference genome. In contrast, for species in the human gut microbiome, a minimum of 10 genomes was required. Again, a minimum number of 30 MAGs per species was required for SGBs to be selected in both datasets.

### Generation of the genome-based metabolic model collection

The GEMs of the analyzed genomes were reconstructed using *gapseq* (v.1.2, sequence DB md5: bf8ba98) [6]. Briefly, the reconstruction process follows these steps: (i) prediction of proteins encoded in the query genome using pyrodigal (v.2.2.0) [59]; (ii) prediction of metabolic and transport pathways by *gapseq* through a similarity search between open reading frames (ORFs) and a database of reference sequences; (iii) reconstruction of a draft metabolic network based on the identified genes; (iv) prediction of a growth medium for the organism based on the metabolic capabilities inferred by the draft model; (v) evaluation of whether gaps in the model’s incomplete metabolic pathways can be filled to enable biomass production using flux balance analysis and the predicted growth medium.

The generation of growth medium for model gapfilling was performed using the prediction module (./*gapseq medium*), which uses logical expressions for the presence and absence of specific pathways and reactions to select compounds to be added to the medium [1]. Oxygen availability was excluded from the predicted media for MAGs belonging to the UHGG dataset (-c: “cpd00007:0”) since species colonizing the lumen of the human gut are adapted to extremely low concentrations of oxygen [60].

### *pan-Draft* metabolic model reconstruction approach

The source code has been integrated into *gapseq* and is accessible at https://github.com/jotech/gapseq through the wrapper script./*gapseq*. The added module for the reconstruction of *pan-Draft* models can be invoked with the following call./*gapseq pan*. Figure 1 provides an overview of how the *pan-Draft* module integrates within the *gapseq* pipeline.

For the reconstruction of species-level GEMs, the draft models (in*.RDS* format) of all the MAGs belonging to the same species are combined. The aim of this step is to assess which reactions share higher frequency among the models in order to identify the centralmetabolism of a species and exclude the rarest functions. Specifically, reaction presence and absence in/from each of the models are summarized in a binary matrix. It is important to notice that multiple copies of a gene generate a unique identifier in the GEMs, therefore the encoded reaction is counted one single time per model. Metabolite names are standardized, giving preference to compounds annotated as cytoplasmic (“-c0”) over the unspecified ones. Simultaneously, reactions are filtered based on the MRF threshold identifying their minimum necessary occurrence frequency in the genome pool—for details on the MRF selection, refer to the “Methods” section below. The reaction IDs are used to extract the reaction information, including the reaction formula and compartment, from the first model having that ID in the genome dataset. Consistency between models generated with the same *gapseq* version guarantees that ModelSEED entries are associated with standardized parameters [43]. The set of selected reactions are then used to build the *pan*-GEM from scratch.

Subsequently, the reaction-to-gene associations (i.e., *rxnXgenes.RDS*) and the reaction weights (i.e., *rxnWeights.RDS*) information of preliminary draft models are merged into summary tables, which are used in the following gapfilling steps. The representative entry of each reaction is selected by identifying the one with the lower weight score assigned by previous runs of the *draft* module. The same entry ID is used to retrieve the reaction-to-gene information. Based on the hypothesis that the reaction frequency within the MAG pool can be used to tune the original weight, this is updated by computing the median of all the corresponding weights of that reaction across draft models. Missing values due to failure to detect a reaction in a fraction of the MAG pool, possibly due to MAG contamination or strain-specific differences, are taken into account by normalizing the number of total weights used to compute the median. Specifically, the maximum weight associated with a reaction (i.e., 100) is added a number of times equal to the number of draft models in the MAG pool that lack genomic sequences retrieving blast hits for that reaction. Additionally, *pan-Draft* requires the pathways tables (i.e., *all-Pathways.tbl*) generated with the *find* module. These tables are summarized in a revised pathway table listing the complete pathways detected in the query draft network with a frequency exceeding the MRF.

In the present study, models generated from MAGs belonging to the same species, determined based on an ANI higher than 95%, were utilized as input for *pan-Draft*. Moreover, to be included in the *pan*-GEM, reactions had to have a MRF higher than 6%.

### Statistical analysis and model quality quantification

The preliminary characterization of the species-level models was performed using two approaches. First, the number of reactions and metabolites in *pan*-GEMs was compared to those of MAG- and iso-GEMs. Second, the distribution of the species pan-reactome across functional groups was analyzed by categorizing reactions according to the first two layers of the MetaCyc pathways ontology, available in the GitHub repository at dat/meta_rea_pwy-gapseq.tbl. Subsequently, the quality of the reconstructed metabolic models was assessed through pairwise network comparisons. The reactions encoded by genes in the reference genomes were aggregated to define the full metabolic potential of a microbial species. The resulting lists of reactions from this process will be referred to as species-representative reactome gold standards. Simultaneously, we also defined the universe of available reactions to generate a bacterial model with *gapseq.* This was done by parsing the "dbhit" column of the “-all-Reactions.tbl” table which identifies pathway predictions in bacterial species mapping to the *gapseq* reaction database. The resulting 7333 reactions were filtered by selecting only those annotated as “approved” or “corrected” in the./dat/seed_reactions_corrected.tsv table, available in the GitHub repository. After filtration, 3760 reactions represented the reaction universe used by *gapseq* to build bacterial GEM. This filtered list was then compared to the species-representative reactome gold standards to define the negative reaction set of each SGB, which includes biochemical transformation outside the metabolic boundaries of the species.

Subsequently, the reactomes of the MAGs were derived from their corresponding GEMs and stored as a binary matrix indicating the presence or absence of reactions in each model (rxn2mod.tsv). To assess accuracy, the MCC was computed by comparing each column of the matrix with the reactome gold standard expressed in a consistent binary form (Fig. 2c, d). This same metric was applied to evaluate the quality of the reconstructed *pan*-GEMs (Figs. 3 and 5), with the MCC formula defined according to Eq. 1.

1 $$\textit{MCC}=\frac{\textit{TN}\times\textit{TP}-\textit{FN}\times\textit{FP}}{\sqrt{(\textit{TP}+\textit{FP})(\textit{TP}+\textit{FN})(\textit{TN}+\textit{FP})(\textit{TN}+\textit{FN})}}$$

Additionally, a generalized additive model fitted to the data to highlight the correlation between GEM quality and MAG completeness (Fig. 2c, d) This was implemented using the “stat_smooth()” function from the “ggplot2” library, with parameters method = “mgcv::gam” and formula = *y* ~ *s*(*x*, bs = “cs”) [61].

### Definition of the minimum reaction frequency in the genome pool

The MRF is the minimum occurrence in the genome pool, expressed in percentage, necessary for a reaction to be included in the *pan*-GEM. This frequency is calculated as the ratio of the number of MAG-GEMs containing the reaction to the total number of MAG-GEMs in that SGB. To determine the MRF necessary for a reaction to be included in the draft metabolic network, the quality of draft *pan*-GEMs was evaluated across various frequency thresholds. Comprehensive evaluations were carried out using thresholds ranging from 0 to 100%, with 1 percentage point intervals. Quality evaluations involved structural network comparisons of the reconstructed *pan*-GEMs with the corresponding species reactome gold standard (Fig. 3). The optimal values for both datasets were determined by selecting the threshold that maximized the average MCC across all different species. Based on the analyzed dataset the final recommended threshold was set at 6%. Additionally, to validate whether the identified threshold is reliable using a varying number of genomes, each SGB was randomly sampled to form batches of 2, 5, 10, 15, 30, 60, 100, and 1000 MAGs, with each sampling process repeated ten times. The optimal MRF for each SGB was determined by computing the median across iterations of the MRF that maximizes the MCC in each batch. The minimum number of MAGs per SGB used as a threshold to include the reaction in the *pan*-GEM was computed as the number of MAGs in the batch multiplied by the MRF. The effect of varying MRF on the quality of *pan*-GEMs was determined by averaging the MCC across the 10 iterations of each SGB.

### Quantify structural improvement of species-level models with respect to MAGs completeness

MAGs from the UHGG database were categorized into groups based on five completeness levels, each set at intervals of 10% points from 50% up to 100% (Fig. 5). Afterwards, iteratively for 10 times, 30 MAGs of each species were randomly selected and used to reconstruct *pan*-GEMs. The reconstructed draft models were gapfilled using a predicted medium, and their quality was quantified by comparison to the species-representative reactome gold standard. In this case, the full metabolic potential of the species was determined as previously, but using the gapfilled version for GEMs derived from the reference genomes. The quality of the GEMs for reference genomes was assessed by comparing the individual reactomes with the species-representative reactome gold standard.

### Validation of fermentation product predictions

To assess the potential of *pan-Draft* to predict bacterial metabolism compared to individual metabolic models from single MAGs, we conducted anaerobic growth simulations on selected species models to compare the predicted export with known fermentation by-products (Additional File 2: Table S3). The method used is an adapted version of the approach implemented by Zimmerman and colleagues [6]. Briefly, preliminary gapfilling of the metabolic networks was performed using *gapseq* (v.1.2, sequence DB md5: bf8ba98) with a complex growth medium whose composition is available in the GitHub repository at dat/media/FT.csv. The same anaerobic medium was employed while performing flux balance analysis (FBA) coupled with minimization of total flux (MTF) and flux variability analysis (FVA) [62, 63]. All the simulations were performed in R (v.4.3.1) using sybil (v.2.2.0) and CPLEX solver (v.22.11) [64–66]. The analysis encompassed eight common anoxic fermentation products for a maximum of 40 different bacterial species for each completeness level (Additional file 2: Table S3). Validation was performed on species that met the following conditions: (i) it was possible to generate at least a set of *pan*-GEMs for the organism, (ii) its fermentation products have been experimentally described either in primary literature or in the NJC19 dataset [40]. Additionally, as described above, models were divided into 5 subsets based on the completeness level of the MAGs used to reconstruct them. Moreover, *pan-Draft* was benchmarked against the GEMs from the MIGRENE collection (see Additional file 1).

To summarize the predictions for individual models of multiple MAGs within a species, we considered metabolites with a positive outflow as actual by-products. Species were considered metabolically capable of secreting a specific compound if positive values were obtained when maximizing the export flux during FVA performed at the maximum growth rate. We set a minimum threshold to include in the fermentation end-product spectrum of the model only metabolites with normalized exchange fluxes (measured in mmol ∗ gDW^−1^) higher than 1e^−4^. Normalization was achieved by rescaling the outflow of individual fermentation products by the predicted growth rate of the respective organism. The performance of the models’ simulations was summarized at the species level by computing the total number of correctly (TP and TN) and incorrectly (FP and FN) predicted by-products over the total number of predictions for the species. This resulted in a measure of the percentage of models that correctly predict the generation of specific metabolites, divided according to the completeness level of the original MAGs. The performances were further summarized by computing the mean and standard deviation across species of simple metrics and the MCC. The degree of improvement between species-level models and models derived from individual MAGs was assessed with a paired *t*-test (R lib. stats, v.4.0.3). The test was implemented by comparing the MCC performance obtained by MAG-GEMs and the corresponding *pan*-GEMs. This was repeated independently for the MTF and the FVA results and for each MAG completeness level.

## Supplementary Information

Additional file 1: Supplementary figures S1-S8. The file includes definitions of pan-reactome terms and describes how the comparison of fermentation product predictions with the MIGRENE model collection was conducted. It presents findings related to the computational cost of the pipeline, the relationship between model quality and MAG completeness, pan-reactome features of species-level metabolic models, and insights into their fermentation capacity predictions. The file also discusses consequences of *Escherichia coli* genomic variability of *pan*-GEMs reconstruction.

Additional file 2: Supplementary tables S1-S6. Table S1. Reference genomes of SGB that passed the filtering in the UHGG and OMD databases. Table S2. Details on the genomes in the SGBs that met the criteria from Table S1. Table S3. Known fermentation products of species with data from primary literature or the NJC19 dataset. Table S4. Comparison of the fermentation product predictions of the best MAG-GEMs and pan-GEMs. Table S5. Results from Minimize-Total-Flux and Flux Variability analyses for pan-GEMs, MAG-GEMs, and iso-GEMs. Table S6: Prediction accuracy of models normalized by the total number of predictions.

Additional file 3. Review history.

## Data Availability

The genome and metagenome collections used in this work include data from the Ocean Microbiomics Database [67] and the Unified Human Gastrointestinal Genome collection [68]. To compare *pan-Draft* with other state-of-the-art tools, species-level GEMs generated using the MIGRENE Toolbox were sourced from the Human Gut Microbiome Atlas [69]. Reference fermentation products of the species were obtained from both primary literature and the NJC19 dataset [40]. *pan-Draft* is coded in R and is freely accessible under the GNU General Public License (v3.0) on GitHub [70]. Comprehensive documentation is provided at https://gapseq.readthedocs.io. All findings presented in this manuscript were generated using *gapseq* version 1.2, the version of the source code used is deposited on Zenodo [71]. Metagenomic datasets used for constructing and validating models were sourced from publicly available databases and referenced in the related sections of the manuscript. The GEMs generated for all the analyzed isolates and MAGs, together with the *pan*-GEMs reconstructed using all available MAGs in a SGB, are available on Zenodo [72]. The provided models are in SBML format and have been gapfilled on the predicted minimal medium (./gapseq medium). Additional scripts employed for the benchmarking can be found on GitHub at the repository: https://github.com/nicola-debernardini/panDraftEval.

## References

[1] Starke S, Harris DMM, Zimmermann J, Schuchardt S, Oumari M, Frank D, et al. Amino acid auxotrophies in human gut bacteria are linked to higher microbiome diversity and long-term stability. ISME J. 2023;17:2370–80. 37891427 10.1038/s41396-023-01537-3 PMC10689445

[2] Basile A, Heinken A, Hertel J, Smarr L, Li W, Treu L, et al. Longitudinal flux balance analyses of a patient with episodic colonic inflammation reveals microbiome metabolic dynamics. Gut Microbes. 2023;15:2226921. 37438876 10.1080/19490976.2023.2226921 PMC10339767

[3] Somerville V, Grigaitis P, Battjes J, Moro F, Teusink B. Use and limitations of genome-scale metabolic models in food microbiology. Curr Opin Food Sci. 2022;43:225–31.

[4] Greses S, De Bernardini N, Treu L, Campanaro S, González-Fernández C. Genome-centric metagenomics revealed the effect of pH on the microbiome involved in short-chain fatty acids and ethanol production. Bioresour Technol. 2023;377:128920. 36934910 10.1016/j.biortech.2023.128920

[5] Saifuddin M, Bhatnagar JM, Segrè D, Finzi AC. Microbial carbon use efficiency predicted from genome-scale metabolic models. Nat Commun. 2019;10:3568. 31395870 10.1038/s41467-019-11488-z PMC6687798

[6] Zimmermann J, Kaleta C, Waschina S. gapseq: informed prediction of bacterial metabolic pathways and reconstruction of accurate metabolic models. Genome Biol. 2021;22:81. 33691770 10.1186/s13059-021-02295-1 PMC7949252

[7] Machado D, Andrejev S, Tramontano M, Patil KR. Fast automated reconstruction of genome-scale metabolic models for microbial species and communities. Nucleic Acids Res. 2018;46:7542–53. 30192979 10.1093/nar/gky537 PMC6125623

[8] Faria JP, Liu F, Edirisinghe JN, Gupta N, Seaver SMD, Freiburger AP, et al. ModelSEED v2: High-throughput genome-scale metabolic model reconstruction with enhanced energy biosynthesis pathway prediction. 2023. Preprint at: http://biorxiv.org/lookup/doi/10.1101/2023.10.04.556561.

[9] Wang H, Marcišauskas S, Sánchez BJ, Domenzain I, Hermansson D, Agren R, et al. RAVEN 2.0: a versatile toolbox for metabolic network reconstruction and a case study on Streptomyces coelicolor. Ouzounis CA, editor. PLOS Comput Biol. 2018;14:e1006541. 30335785 10.1371/journal.pcbi.1006541 PMC6207324

[10] Capela J, Lagoa D, Rodrigues R, Cunha E, Cruz F, Barbosa A, et al. merlin, an improved framework for the reconstruction of high-quality genome-scale metabolic models. Nucleic Acids Res. 2022;50:6052–66. 35694833 10.1093/nar/gkac459 PMC9226533

[11] Aite M, Chevallier M, Frioux C, Trottier C, Got J, Cortés MP, et al. Traceability, reproducibility and wiki-exploration for “à-la-carte” reconstructions of genome-scale metabolic models. Nielsen J, editor. PLOS Comput Biol. 2018;14:e1006146. 29791443 10.1371/journal.pcbi.1006146 PMC5988327

[12] Garza DR, Von Meijenfeldt FAB, Van Dijk B, Boleij A, Huynen MA, Dutilh BE. Nutrition or nature: using elementary flux modes to disentangle the complex forces shaping prokaryote pan-genomes. BMC Ecol Evol. 2022;22:101. 35974327 10.1186/s12862-022-02052-3 PMC9382767

[13] Bernstein DB, Sulheim S, Almaas E, Segrè D. Addressing uncertainty in genome-scale metabolic model reconstruction and analysis. Genome Biol. 2021;22:64. 33602294 10.1186/s13059-021-02289-z PMC7890832

[14] Zampieri G, Campanaro S, Angione C, Treu L. Metatranscriptomics-guided genome-scale metabolic modeling of microbial communities. Cell Rep Methods. 2023;3: 100383. 36814842 10.1016/j.crmeth.2022.100383 PMC9939383

[15] Orellana E, Guerrero LD, Davies-Sala C, Altina M, Pontiggia RM, Erijman L. Extracellular hydrolytic potential drives microbiome shifts during anaerobic co-digestion of sewage sludge and food waste. Bioresour Technol. 2022;343: 126102. 34634462 10.1016/j.biortech.2021.126102

[16] Chen C, Liao C, Liu Y-Y. Teasing out missing reactions in genome-scale metabolic networks through hypergraph learning. Nat Commun. 2023;14:2375. 37185345 10.1038/s41467-023-38110-7 PMC10130184

[17] Ong WK, Midford PE, Karp PD. Taxonomic weighting improves the accuracy of a gap-filling algorithm for metabolic models. Cowen L, editor. Bioinformatics. 2020;36:1823–30. 31688932 10.1093/bioinformatics/btz813 PMC7523652

[18] Eisenhofer R, Odriozola I, Alberdi A. Impact of microbial genome completeness on metagenomic functional inference. ISME Commun. 2023;3:12. 36797336 10.1038/s43705-023-00221-z PMC9935889

[19] Zorrilla F, Buric F, Patil KR, Zelezniak A. metaGEM: reconstruction of genome scale metabolic models directly from metagenomes. Nucleic Acids Res. 2021;49:e126–e126. 34614189 10.1093/nar/gkab815 PMC8643649

[20] Meziti A, Rodriguez-R LM, Hatt JK, Peña-Gonzalez A, Levy K, Konstantinidis KT. The Reliability of Metagenome-Assembled Genomes (MAGs) in representing natural populations: insights from comparing MAGs against isolate genomes derived from the same fecal sample. McBain AJ, editor. Appl Environ Microbiol. 2021;87:e02593-20. 33452027 10.1128/AEM.02593-20 PMC8105024

[21] De Bernardini N, Basile A, Zampieri G, Kovalovszki A, De Diego DB, Offer E, et al. Integrating metagenomic binning with flux balance analysis to unravel syntrophies in anaerobic CO2 methanation. Microbiome. 2022;10:117. 35918706 10.1186/s40168-022-01311-1 PMC9347119

[22] Amann RI, Ludwig W, Schleifer KH. Phylogenetic identification and in situ detection of individual microbial cells without cultivation. Microbiol Rev. 1995;59:143–69. 7535888 10.1128/mr.59.1.143-169.1995 PMC239358

[23] Hugenholtz P, Goebel BM, Pace NR. Impact of Culture-Independent Studies on the Emerging Phylogenetic View of Bacterial Diversity. J Bacteriol. 1998;180:4765–74. 9733676 10.1128/jb.180.18.4765-4774.1998 PMC107498

[24] The Genome Standards Consortium, Bowers RM, Kyrpides NC, Stepanauskas R, Harmon-Smith M, Doud D, et al. Minimum information about a single amplified genome (MISAG) and a metagenome-assembled genome (MIMAG) of bacteria and archaea. Nat Biotechnol. 2017;35:725–31. 28787424 10.1038/nbt.3893 PMC6436528

[25] Almeida A, Nayfach S, Boland M, Strozzi F, Beracochea M, Shi ZJ, et al. A unified catalog of 204,938 reference genomes from the human gut microbiome. Nat Biotechnol. 2021;39:105–14. 32690973 10.1038/s41587-020-0603-3 PMC7801254

[26] Paoli L, Ruscheweyh H-J, Forneris CC, Hubrich F, Kautsar S, Bhushan A, et al. Biosynthetic potential of the global ocean microbiome. Nature. 2022;607:111–8. 35732736 10.1038/s41586-022-04862-3 PMC9259500

[27] Nayfach S, Roux S, Seshadri R, Udwary D, Varghese N, Schulz F, et al. A genomic catalog of Earth’s microbiomes. Nat Biotechnol. 2021;39:499–509. 33169036 10.1038/s41587-020-0718-6 PMC8041624

[28] Centurion VB, Rossi A, Orellana E, Ghiotto G, Kakuk B, Morlino MS, et al. A unified compendium of prokaryotic and viral genomes from over 300 anaerobic digestion microbiomes. Environ Microbiome. 2024;19:1. 38167520 10.1186/s40793-023-00545-2 PMC10762816

[29] Konstantinidis KT, Tiedje JM. Genomic insights that advance the species definition for prokaryotes. Proc Natl Acad Sci. 2005;102:2567–72. 15701695 10.1073/pnas.0409727102 PMC549018

[30] Seif Y, Kavvas E, Lachance J-C, Yurkovich JT, Nuccio S-P, Fang X, et al. Genome-scale metabolic reconstructions of multiple Salmonella strains reveal serovar-specific metabolic traits. Nat Commun. 2018;9:3771. 30218022 10.1038/s41467-018-06112-5 PMC6138749

[31] Lu H, Kerkhoven EJ, Nielsen J. A Pan-Draft Metabolic Model Reflects Evolutionary Diversity across 332 Yeast Species. Biomolecules. 2022;12: 1632. 36358981 10.3390/biom12111632 PMC9687678

[32] Blázquez B, San León D, Rojas A, Tortajada M, Nogales J. New Insights on Metabolic Features of Bacillus subtilis Based on Multistrain Genome-Scale Metabolic Modeling. Int J Mol Sci. 2023;24: 7091. 37108252 10.3390/ijms24087091 PMC10138676

[33] Mirhakkak MH, Chen X, Ni Y, Heinekamp T, Sae-Ong T, Xu L-L, et al. Genome-scale metabolic modeling of Aspergillus fumigatus strains reveals growth dependencies on the lung microbiome. Nat Commun. 2023;14:4369. 37474497 10.1038/s41467-023-39982-5 PMC10359302

[34] Heinken A, Thiele I. Microbiome Modelling Toolbox 2.0: efficient, tractable modelling of microbiome communities. Wren J, editor. Bioinformatics. 2022;38:2367–8. 35157025 10.1093/bioinformatics/btac082 PMC9004645

[35] Bidkhori G, Shoaie S. MIGRENE: the toolbox for microbial and individualized gems reactobiome and community network modelling. Metabolites. 2024;14:132. 38535292 10.3390/metabo14030132 PMC10972203

[36] Heinken A, Hertel J, Acharya G, Ravcheev DA, Nyga M, Okpala OE, et al. Genome-scale metabolic reconstruction of 7,302 human microorganisms for personalized medicine. Nat Biotechnol. 2023. https://www.nature.com/articles/s41587-022-01628-0. 10.1038/s41587-022-01628-0 PMC10497413 36658342

[37] Hyun JC, Monk JM, Palsson BO. Comparative pangenomics: analysis of 12 microbial pathogen pangenomes reveals conserved global structures of genetic and functional diversity. BMC Genomics. 2022;23:7. 34983386 10.1186/s12864-021-08223-8 PMC8725406

[38] Tantoso E, Eisenhaber B, Kirsch M, Shitov V, Zhao Z, Eisenhaber F. To kill or to be killed: pangenome analysis of Escherichia coli strains reveals a tailocin specific for pandemic ST131. BMC Biol. 2022;20:146. 35710371 10.1186/s12915-022-01347-7 PMC9205054

[39] Shoer S, Reicher L, Pilpel Y, Segal E. Pangenomes of Human Gut Microbiota Uncover Links Between Genetic Diversity and Stress Response. 2024. Preprint at http://biorxiv.org/lookup/doi/10.1101/2024.04.17.589959. 10.1016/j.chom.2024.08.017 PMC12060796 39353429

[40] Lim R, Cabatbat JJT, Martin TLP, Kim H, Kim S, Sung J, et al. Large-scale metabolic interaction network of the mouse and human gut microbiota. Sci Data. 2020;7:204. 32591517 10.1038/s41597-020-0516-5 PMC7320173

[41] Caspi R, Altman T, Billington R, Dreher K, Foerster H, Fulcher CA, et al. The MetaCyc database of metabolic pathways and enzymes and the BioCyc collection of Pathway/Genome Databases. Nucleic Acids Res. 2014;42:D459–71. 24225315 10.1093/nar/gkt1103 PMC3964957

[42] Kanehisa M, Sato Y, Furumichi M, Morishima K, Tanabe M. New approach for understanding genome variations in KEGG. Nucleic Acids Res. 2019;47:D590–5. 30321428 10.1093/nar/gky962 PMC6324070

[43] Henry CS, DeJongh M, Best AA, Frybarger PM, Linsay B, Stevens RL. High-throughput generation, optimization and analysis of genome-scale metabolic models. Nat Biotechnol. 2010;28:977–82. 20802497 10.1038/nbt.1672

[44] Mise K, Iwasaki W. Unexpected absence of ribosomal protein genes from metagenome-assembled genomes. ISME Commun. 2022;2:118. 37938339 10.1038/s43705-022-00204-6 PMC9723686

[45] Liu S, Moon CD, Zheng N, Huws S, Zhao S, Wang J. Opportunities and challenges of using metagenomic data to bring uncultured microbes into cultivation. Microbiome. 2022;10:76. 35546409 10.1186/s40168-022-01272-5 PMC9097414

[46] Palù M, Basile A, Zampieri G, Treu L, Rossi A, Morlino MS, et al. KEMET – A python tool for KEGG Module evaluation and microbial genome annotation expansion. Comput Struct Biotechnol J. 2022;20:1481–6. 35422973 10.1016/j.csbj.2022.03.015 PMC8976094

[47] Puchol-Royo R, Pascual J, Ortega-Legarreta A, Otto P, Tideman J, De Vries S-J, et al. Unveiling the ecology, taxonomy and metabolic capabilities of MBA03, a potential key player in anaerobic digestion. 2023. Preprint at: http://biorxiv.org/lookup/doi/10.1101/2023.09.08.556800. 10.1111/1751-7915.70258 PMC12663870 41316964

[48] Roodgar M, Good BH, Garud NR, Martis S, Avula M, Zhou W, et al. Longitudinal linked-read sequencing reveals ecological and evolutionary responses of a human gut microbiome during antibiotic treatment. Genome Res. 2021;31:1433–46. 34301627 10.1101/gr.265058.120 PMC8327913

[49] Olm MR, Crits-Christoph A, Bouma-Gregson K, Firek BA, Morowitz MJ, Banfield JF. inStrain profiles population microdiversity from metagenomic data and sensitively detects shared microbial strains. Nat Biotechnol. 2021;39:727–36. 33462508 10.1038/s41587-020-00797-0 PMC9223867

[50] Quince C, Nurk S, Raguideau S, James R, Soyer OS, Summers JK, et al. STRONG: metagenomics strain resolution on assembly graphs. Genome Biol. 2021;22:214. 34311761 10.1186/s13059-021-02419-7 PMC8311964

[51] Feng Z, Clemente JC, Wong B, Schadt EE. Detecting and phasing minor single-nucleotide variants from long-read sequencing data. Nat Commun. 2021;12:3032. 34031367 10.1038/s41467-021-23289-4 PMC8144375

[52] Kazantseva E, Donmez A, Frolova M, Pop M, Kolmogorov M. Strainy: phasing and assembly of strain haplotypes from long-read metagenome sequencing. Preprint at: 2023. http://biorxiv.org/lookup/doi/10.1101/2023.01.31.526521. 10.1038/s41592-024-02424-1 PMC13005716 39327484

[53] Zheng W, Zhao S, Yin Y, Zhang H, Needham DM, Evans ED, et al. High-throughput, single-microbe genomics with strain resolution, applied to a human gut microbiome. Science. 2022;376: eabm1483. 35653470 10.1126/science.abm1483

[54] Richardson L, Allen B, Baldi G, Beracochea M, Bileschi ML, Burdett T, et al. MGnify: the microbiome sequence data analysis resource in 2023. Nucleic Acids Res. 2023;51:D753–9. 36477304 10.1093/nar/gkac1080 PMC9825492

[55] Lobanov V, Gobet A, Joyce A. Ecosystem-specific microbiota and microbiome databases in the era of big data. Environ Microbiome. 2022;17:37. 35842686 10.1186/s40793-022-00433-1 PMC9287977

[56] Olm MR, Brown CT, Brooks B, Banfield JF. dRep: a tool for fast and accurate genomic comparisons that enables improved genome recovery from metagenomes through de-replication. ISME J. 2017;11:2864–8. 28742071 10.1038/ismej.2017.126 PMC5702732

[57] Chaumeil P-A, Mussig AJ, Hugenholtz P, Parks DH. GTDB-Tk: a toolkit to classify genomes with the genome taxonomy database. Hancock J, editor. Bioinformatics. 2020;36:1925–7. 10.1093/bioinformatics/btz848 PMC7703759 31730192

[58] Parks DH, Imelfort M, Skennerton CT, Hugenholtz P, Tyson GW. CheckM: assessing the quality of microbial genomes recovered from isolates, single cells, and metagenomes. Genome Res. 2015;25:1043–55. 25977477 10.1101/gr.186072.114 PMC4484387

[59] Larralde M. Pyrodigal: Python bindings and interface to Prodigal, an efficient method for gene prediction in prokaryotes. J Open Source Softw. 2022;7:4296.

[60] Albenberg L, Esipova TV, Judge CP, Bittinger K, Chen J, Laughlin A, et al. Correlation Between Intraluminal Oxygen Gradient and Radial Partitioning of Intestinal Microbiota. Gastroenterology. 2014;147:1055-1063.e8. 25046162 10.1053/j.gastro.2014.07.020 PMC4252572

[61] Wickham H, Chang W, Henry L, Lin Pedersen T, Takahashi K, Wilke C, et al. ggplot2: Elegant Graphics for Data Analysis. Springer-Verlag New York; 2016. Available from: https://ggplot2.tidyverse.org.

[62] Mahadevan R, Schilling CH. The effects of alternate optimal solutions in constraint-based genome-scale metabolic models. Metab Eng. 2003;5:264–76. 14642354 10.1016/j.ymben.2003.09.002

[63] Holzhütter H. The principle of flux minimization and its application to estimate stationary fluxes in metabolic networks. Eur J Biochem. 2004;271:2905–22. 15233787 10.1111/j.1432-1033.2004.04213.x

[64] Cplex, I. I. V12. 1: User’s Manual for CPLEX. International Business Machines Corporation. 2009. Available from: https://www.ibm.com/products/ilog-cplex-optimization-studio.

[65] R Core Team. R. A language and environment for statistical computing. R Foundation for Statistical Computing, Vienna, Austria. 2024. Available from: https://www.R-project.org/.

[66] Gelius-Dietrich G, Desouki AA, Fritzemeier CJ, Lercher MJ. sybil – Efficient constraint-based modelling in R. BMC Syst Biol. 2013;7: 125. 24224957 10.1186/1752-0509-7-125 PMC3843580

[67] Paoli L, Ruscheweyh H-J, Forneris CC, Hubrich F, Kautsar S, Bhushan A, et al. Ocean Microbiomics Database. Datasets. 2022. https://microbiomics.io/ocean/.

[68] Almeida A, Nayfach S, Boland M, Strozzi F, Beracochea M, Shi ZJ, et al. Unified Human Gastrointestinal Genome collection. Datasets. 2020. http://ftp.ebi.ac.uk/pub/databases/metagenomics/mgnify_genomes/human-gut/.

[69] Bidkhori G, Shoaie S. Human Gut Microbiome Atlas. 2022 https://www.microbiomeatlas.org/.

[70] De Bernardini N, Zampieri G, Campanaro S, Zimmermann J, Waschina S, Treu L. pan-Draft: Automated reconstruction of species-representative metabolic models from multiple genomes. Github. 2024. https://github.com/jotech/gapseq/. 10.1186/s13059-024-03425-1 PMC11515315 39456096

[71] De Bernardini N, Zampieri G, Campanaro S, Zimmermann J, Waschina S, Treu L. pan-Draft: gapseq source code. 2024. 10.5281/zenodo.12912033

[72] De Bernardini N, Zampieri G, Campanaro S, Zimmermann J, Waschina S, Treu L. pan-Draft models for 75 UHGG and 8 OMD bacterial species. 2024. 10.5281/zenodo.12806931.

