# Supplementary material for: pan-Draft: automated reconstruction of species-representative metabolic models from multiple genomes
Source: Genome Biol. 2024 Oct 25;25:280. doi: 10.1186/s13059-024-03425-1 (PMC11515315; doi:10.1186/s13059-024-03425-1)
Supplement: Supplementary file 1 — Additional file 1: Supplementary figures S1-S8. The file includes definitions of pan-reactome terms and describes how the comparison of fermentation product predictions with the MIGRENE model collection was conducted. It presents findings related to the computational cost of the pipeline, the relationship between model quality and MAG completeness, pan-reactome features of species-level metabolic models, and insights into their fermentation capacity predictions. The file also discusses consequences of Escherichia coli genomic variability of pan-GEMs reconstruction. [file 13059_2024_3425_MOESM1_ESM.docx]

*pan-Draft*: Automated reconstruction of species-representative metabolic models from multiple genomes

Nicola De Bernardini^a^, Guido Zampieri^a*^, Stefano Campanaro^a*^, Johannes Zimmermann^b,d^, Silvio Waschina^c#^, Laura Treu^a#^.

^a^ Department of Biology, University of Padova, Via U. Bassi 58/b, 35121, Padova, Italy

^b^ Evolutionary Ecology and Genetics, Zoological Institute, Kiel University, 24118, Kiel, Germany

^c^ Department of Human Nutrition and Food Science, Kiel University, Heinrich-Hecht-Platz 10, 24118, Kiel, Germany
^d^ Antibiotic resistance group, Max Planck Institute for Evolutionary Biology, 24306, Ploen, Germany

^*^Corresponding authors:

Stefano Campanaro (stefano[.campanaro@unipd.it](mailto:laura.treu@unipd.it)), Department of Biology, University of Padova, Via U. Bassi 58/b, 35121 Padova, Italy.

Guido Zampieri (guido.zampieri@unipd.it), Department of Biology, University of Padova, Via U. Bassi 58/b, 35121 Padova, Italy.

## Additional methods

### Definition of pan-reactome terms

To facilitate the understanding of pangenomic analysis, the genes in explored sequences are usually classified based on their frequency. The defined categories aim to clarify the distribution and significance of genes and biological functions within a pan-genome, providing insights into both the conserved and variable aspects of a species’ genome. Here, we similarly define key terms to present the features of species-level genome bin (SGB) metabolism, which are used to describe the output of *pan-Draft* and correspond to metabolic reactions in a *pan*-GEM. The terms correspond to the following categories:

- Strict core: this category includes reactions with a frequency of 100%, meaning these reactions are present in all the genomes analyzed.
- Core: this category includes reactions with a frequency above 95%, meaning that these reactions are present in most genomes. It includes reactions that are widespread and relevant for a species' primary metabolic processes. This category includes reactions that could be absent from the strict core category due to the incompleteness of MAGs.
- Shell: this category includes reactions with a frequency between 5% and 95%. These reactions are not universally present across the genomes and may represent functions that are beneficial but not strictly essential.
- Cloud: this category includes reactions with a frequency below 5%, meaning that these reactions are present in only a small subset of genomes. It includes reactions that are often associated with specialized functions, such as environmental adaptation or resistance to specific conditions.

### Comparison of fermentation product predictions with a state-of-the-art tool

*pan-Draft* was benchmarked against models in the MIGRENE collection using a strategy similar to the one described in the main text. Species were selected for the analysis only when matching pairs could be identified between the MIGRENE collection and the SGBs analyzed here [1]. Species were matched based on their taxonomy at the species level. This resulted in assessing the production of eight anoxic fermentation products for 36 bacterial species (Additional File 2: Table S3). The fermentation capacity for *pan*-GEMs was assessed by randomly sampling 30 MAGs with a completeness between 90 and 100% from each SGB. A complex growth medium (available in the GitHub repository at dat/media/FT.csv) was employed for both MTF and FVA, and simulations were performed in R (v.4.3.1) using sybil (v.2.2.0) and the CPLEX solver (v.22.11). Like in the tests described in the main text, every metabolite with an outflow flux higher than 1e-4 mmol∗gDW^−1^ was counted as a positive prediction.

## Additional results

### Computational cost of the pipeline

The time required for reconstructing *pan*-GEMs varies significantly depending on the number of MAGs involved in species-level model generation. On average, generating a single GEM with up to two threads requires about two hours, with the most computationally expensive step being the identification of metabolic pathways (Table S1).

**Table S1. Computational cost of the reconstructions steps*.*** Statistics were estimated for the four *gapseq* modules necessary to generate functional GEMs and averaging results over 15,000 model reconstructions.

| ***Reconstruction step*** | ***Average time*** *(hh:mm:ss)* | ***Standard deviation*** *(hh:mm:ss)* | ***Computed on n model reconstructions*** | ***Num. of used cores*** |
| --- | --- | --- | --- | --- |
| ***gapseq find*** | *01:58:20* | *00:24:40* | *15871* | *2* |
| ***gapseq find_transport*** | *00:06:38* | *00:02:55* | *15924* | *1* |
| ***gapseq draft*** | *00:05:32* | *00:01:59* | *15368* | *1* |
| ***gapseq fill*** | *00:04:30* | *00:00:35* | *14258* | *2* |

Within this framework, *pan-Draft* had a marginal cost, demonstrating to scale proportionally with the number of models processed (Fig. S1). Indeed, it required approximately 23 minutes to process more than 4,500 GEMs with a single core. Given the low memory requirements (<2 GB) and the ability to use a single thread per GEM, we believe the process can be efficiently parallelized. This is especially true for reconstructing *pan*-GEMs of environmental communities, which typically include a limited subset of SGBs with more than 30 MAGs, making them manageable on a small workstation. However, generating *pan*-GEMs for model species, such as *Escherichia coli*, which count over 2,000 MAGs in the UHGG database, will likely necessitate high-performance computing. When applying *pan-Draft* on extensively studied species it may be computationally more efficient to select a subsample of the total available MAGs.

*
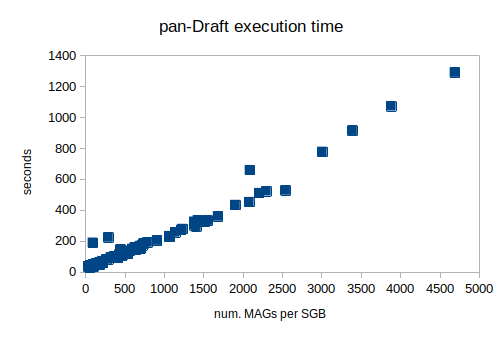
*

**Fig. S1. Computational cost of *pan-Draft*.** Relationship between computing time (in seconds) and number of processed GEMs*.*

### Relation between model quality and MAG completeness level

*
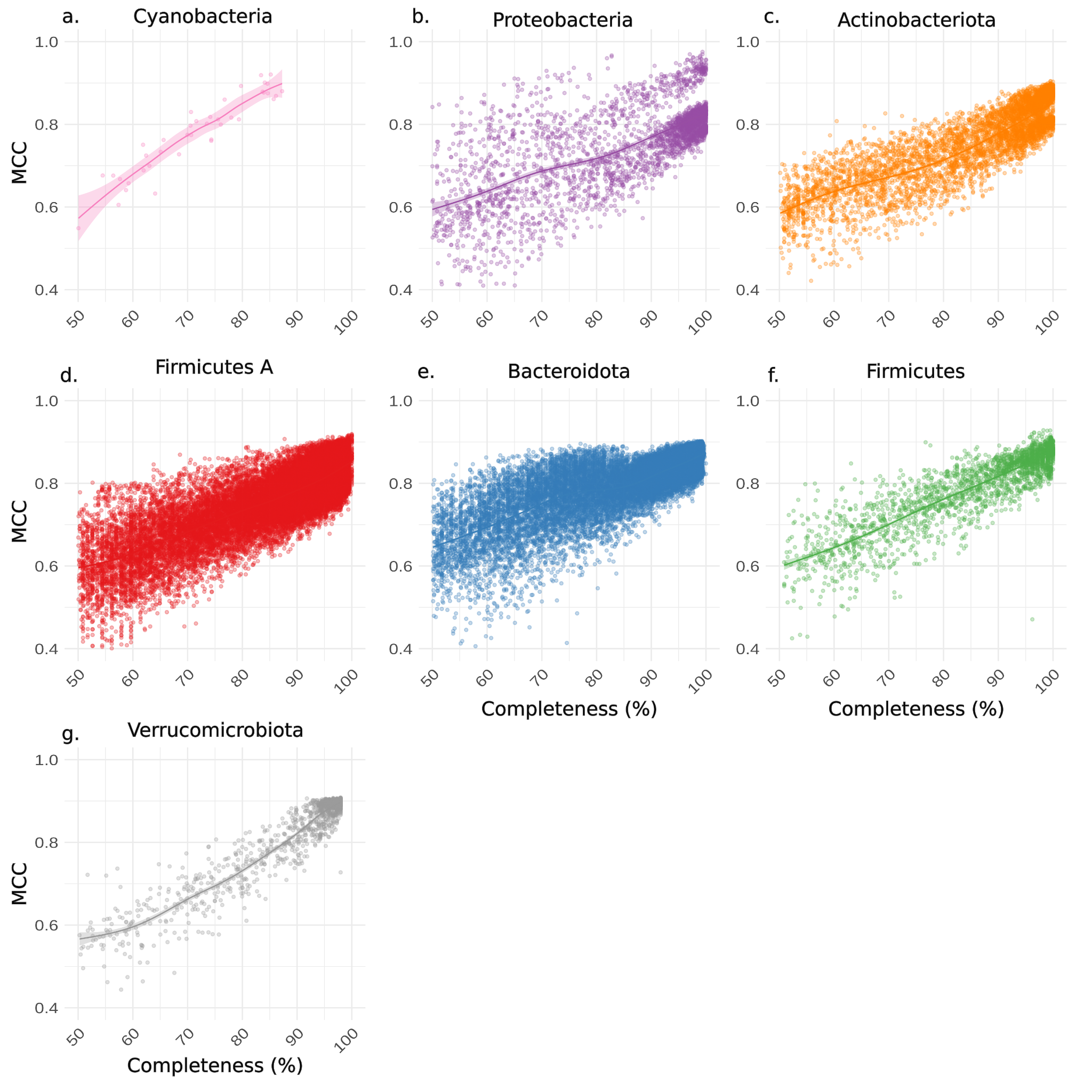
*

**Fig. S2. Comparative analysis of gapfilled metabolic models across phyla for different MAG completeness levels.** Panels depict the Matthews correlation coefficient (MCC) measuring structural similarity between gapfilled GEMs for 84 SGBs and their species-representative reactome gold standard. Results are shown for SGB in both the UHGG and OMD dataset including Cyanobacteria (**a**), Proteobacteria (**b**)**,** Actinobacteriota (**c**), Firmicutes A (**d**), Bacteroidota (**e**), Firmicutes (**f**), and Verrucomicrobiota (**g**).

### Pan-reactome features of species-level metabolic models


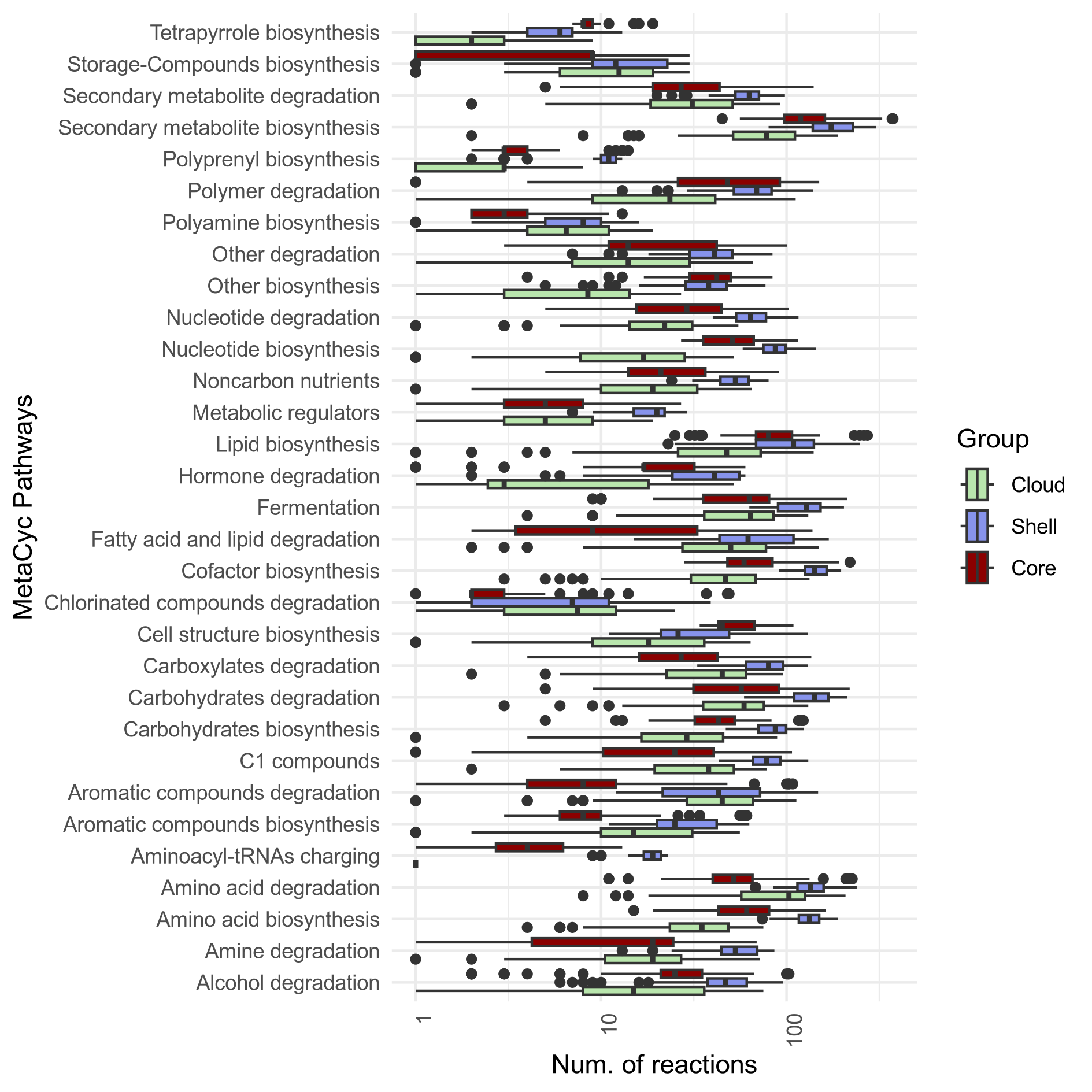


**Fig. S3. Pan-reactome features of *pan*-GEMs.** Classification of reactions grouped into pan-reactome categories by MetaCyc pathways ontology, focusing on the second layer of the metabolic pathways hierarchy under the “Degradation” and “Biosynthesis” classes. Core, shell, and cloud consist of all reactions with a frequency higher than 95%, between 5% and 95%, and below 5%, respectively, in all the GEMs of any SGB.

### Linking *Escherichia coli* genomic variability to model quality: insights from pan-reactome content

On average, the iso-GEMs of *E. coli* exhibited a quality that was 0.18 MCC points lower than the iso-GEMs of the other species and this caused the models to stand out as outliers compared to other SGBs. Due to this marked difference, further independent analyses were conducted on *E. coli*. An hypothesis was formulated linking the observed discrepancy to the genetic variability within the species. It was expected that large pan-genomes, such as the one of *E. coli* [2], could impact positively the total number of reactions included in the reactome gold standard (R_gs_). A large R_gs_ compared to the genetic content of individual genomes can in fact reduce the estimated structural quality of individual MAG- and iso-GEMs. The variability within R_gs_ is further supported by the fact that distinct subgroups, each with specific genetic profiles, can be identified in *E. coli* [3]. In this context, the hierarchical clustering of isolates based on pairwise ANI revealed that the UHGG database contains distinct species subgroups (Fig. S4).


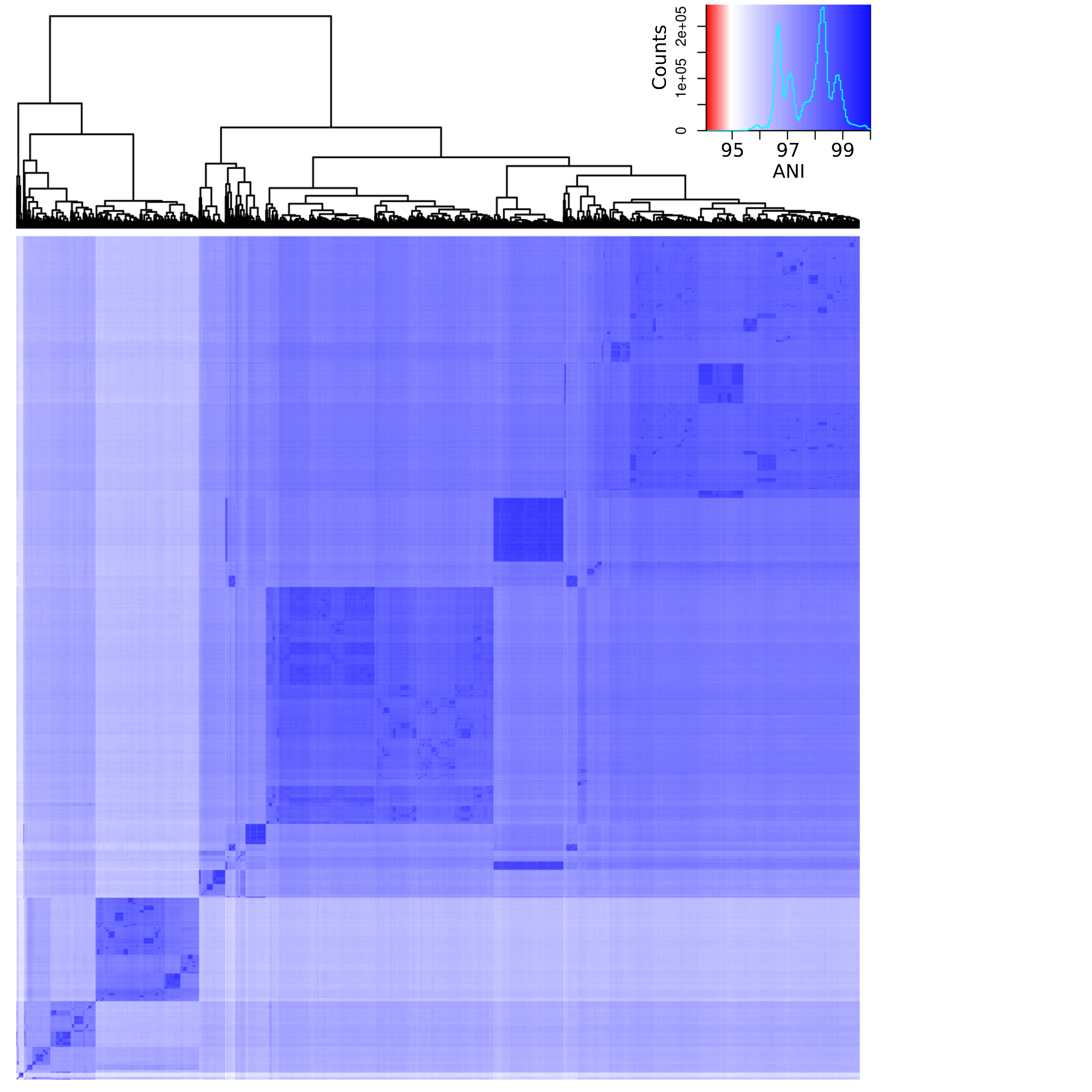


**Fig. S4. Hierarchical clustering of 2,160 *E. coli* isolates based on pairwise ANI.** The clustering highlights clear genomic similarities within subgroups (**a**). The density plot shows four distinct peaks, indicating that similarities between subgroups are concentrated around specific ANI values (**b**).

However, further investigation of the *E. coli* SGB revealed a simpler yet subtle explanation for the discrepancy in model quality. R_gs_ can capture the genetic variability of a species only when a large number of genomes are analyzed. This principle is central to describing an accurate trajectory of pan-genomes and was clearly shown by Shoer et al. (2024), who highlighted the strong correlation existing between the number of genes in a pan-genome and the number of strains used to construct it [4]. Here, a clear negative correlation was observed between the average MCC of iso-GEMs and the number of isolates used to define R_gs_ (linear regression model, p-value < 2.4e-8; Fig. S5a). This trend suggests that SGBs with a higher number of isolates accumulate in their R_gs_ more unique genetic features. Indeed, the number of reactions unique to a single iso-GEMs or present in less than 5% of the total iso-GEMs of a SGBs showed a strong positive correlation with the number of isolates (Fig. S5b). These cloud features are likely absent in individual GEMs, thus increasing the number of FN and lowering the overall MCC when their reaction content is compared to R_gs_.

Despite these considerations, comparing MAG-GEMs with *pan*-GEMs showed structural improvements in the latter, particularly for models of highly incomplete genomes and consistently with the observations in other SGBs (Fig. S5c). The comparison also demonstrated that in this species using a MRF of 6% caused the *pan*-GEMs to overestimate the reaction content of individual iso-GEMs. This threshold likely filters out unique model differences, such as those caused by contamination, but does not select strain-specific reactions when different subgroups of the species are analyzed together. Therefore, the *pan*-GEMs end up representing mixed metabolic capacities of different phylogroups. Since distinct subgroups are characterized by higher genetic similarities, a possible solution to prevent the generation of hybrid GEMs in species such as *E. coli* consists in splitting the genomes in groups a priori. As shown above, clustering the genomes based on ANI similarity can help to identify subgroups which can then be processed independently to reconstruct phylogroup-specific *pan*-GEMs.


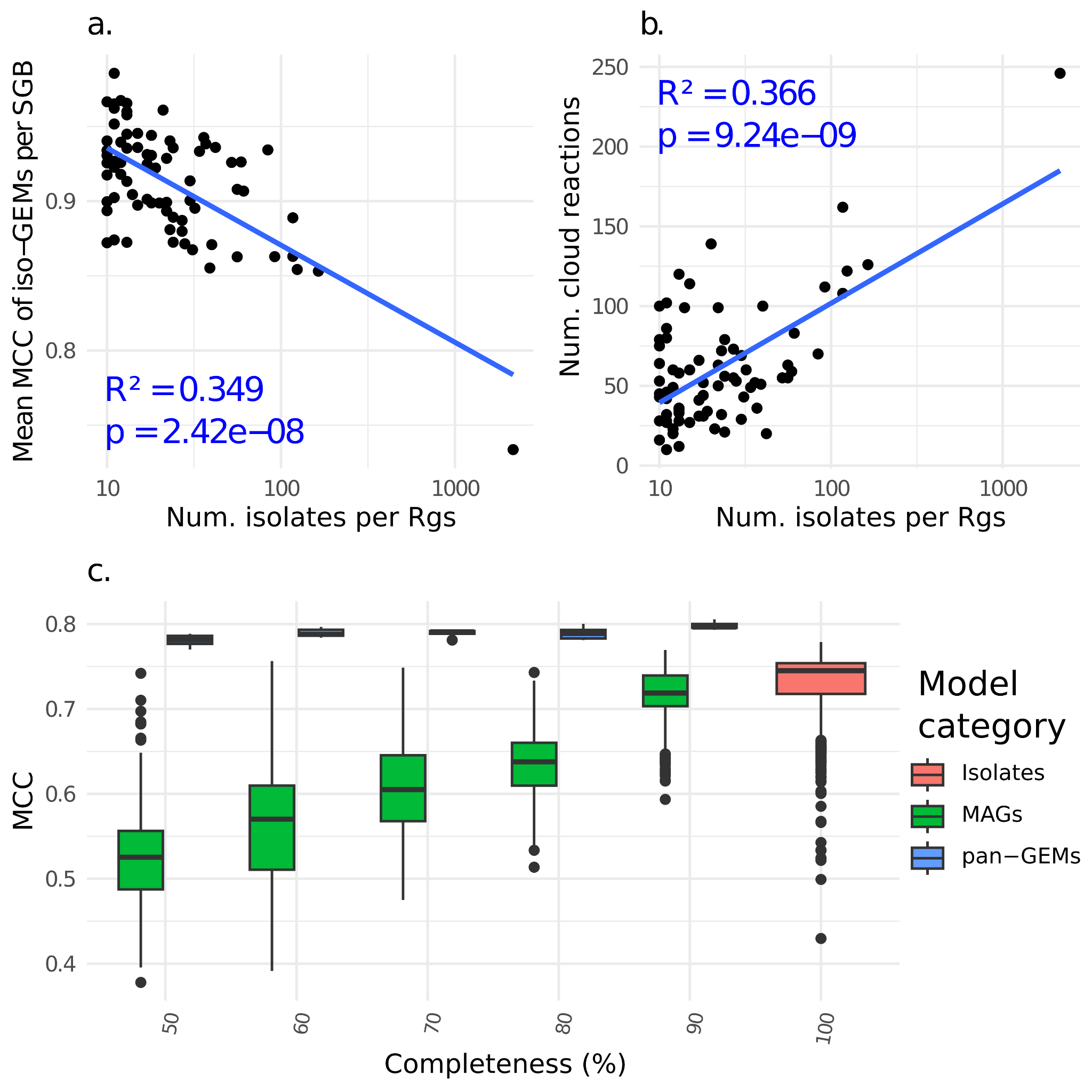


**Fig. S5. Effect of the number of isolates used to define R_gs_ on the estimated structural quality of GEMs.** Linear regression models show the correlation between the number of isolates per SGB used to define the R_gs_ and the mean structural quality of the iso-GEMs (**a**), as well as the correlation with the number of cloud reactions in R_gs_ (**b**). The figure also compares the structural quality estimates for *pan*-GEMs against MAG-GEMs and iso-GEMs in *E. coli* (**c**).

### Structural improvement of species-level metabolic models


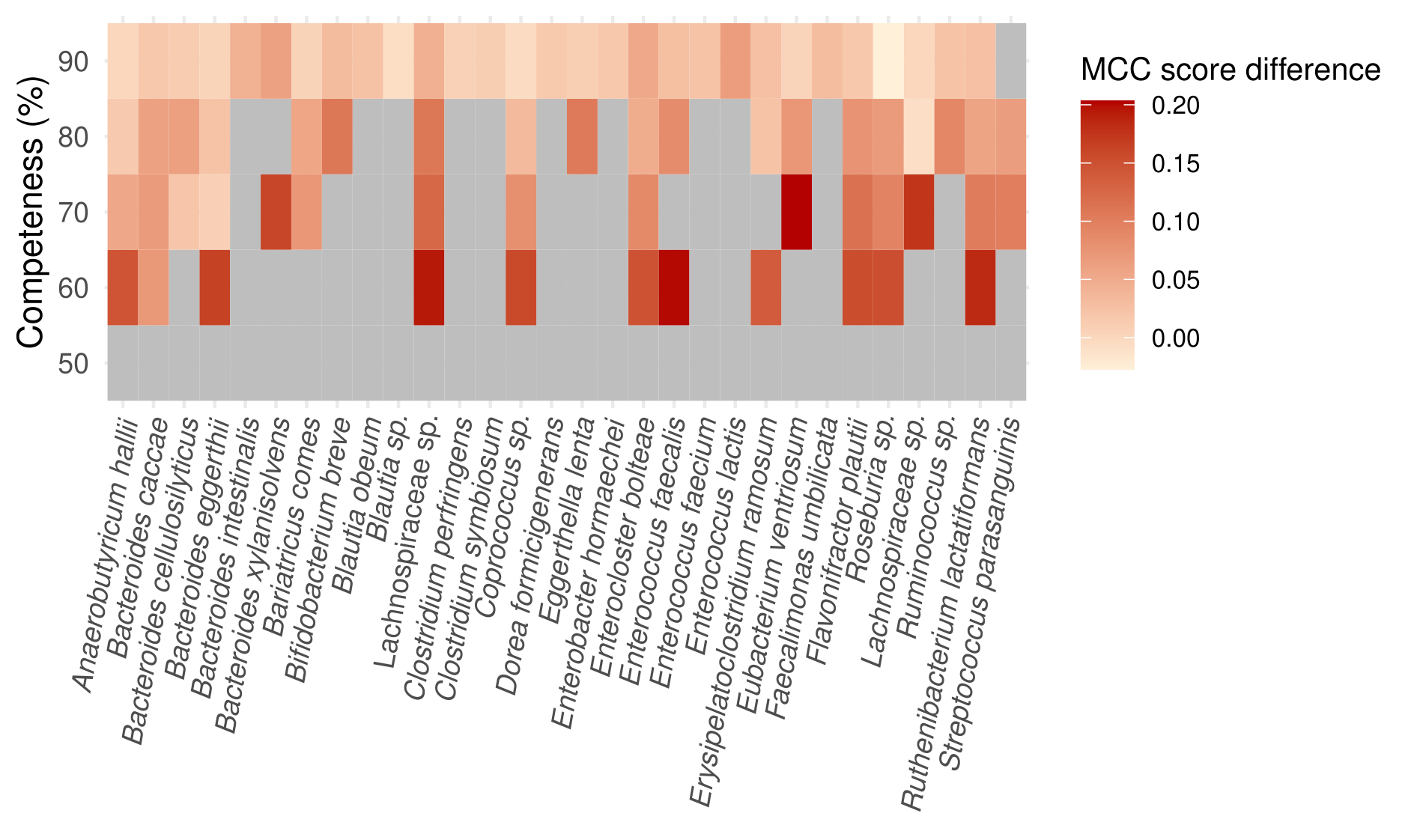


**Fig. S6. Enhancement in structural quality of *pan*-GEMs compared to MAG models.** The heatmap shows the average difference in MCC between each species' *pan*-GEM and the GEM from its most complete MAG. The analysis was conducted at the species level, with MAGs grouped based on their completeness. Threshold values indicate the categories used to partition the MAGs into five groups. Gray boxes denote missing *pan*-GEMs, a condition occurring when the subset contains fewer than 30 available MAGs. All statistics are based on gapfilled models.

### Insights into fermentation capacity predictions

**
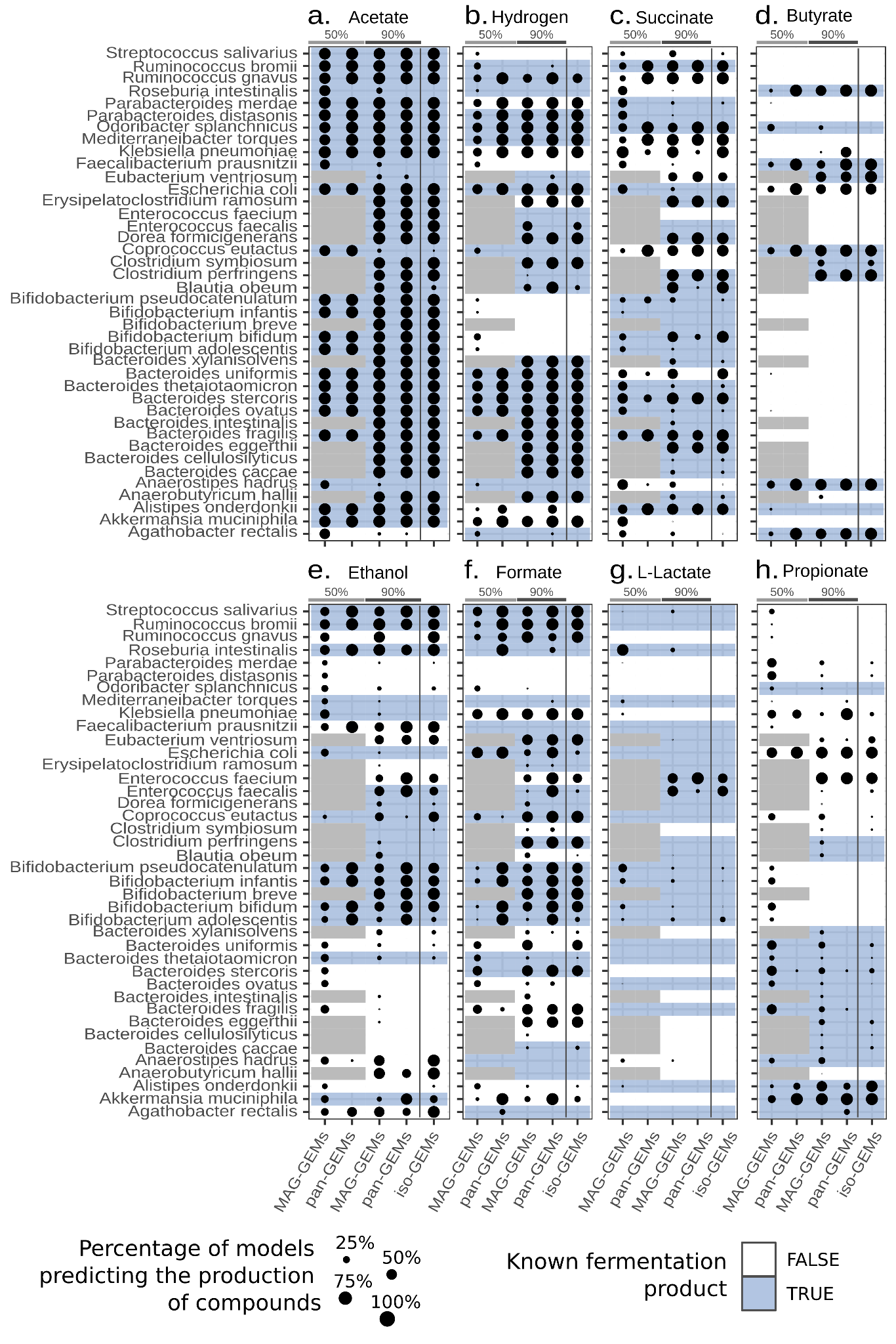
**

**Fig. S7. Percentage of MAG-GEMs and *pan*-GEMs predicting the production of tested compounds.** Results of the fermentation product test under anaerobic growth obtained with MTF flux balance analysis for MAG-GEMs, *pan*-GEMs, and iso-GEMs (last column in each heatmap). Species-level models and MAG-GEMs were derived from MAGs with completeness level between 50 to 60% (first and second column) and 90 to 100% (third and fourth column). Point sizes indicate the percentage of models of a species (row) that were predicted to produce a fermentation product metabolite with a rate higher than 1e-4 mmol∗gDW^−1^. Predicted exports are depicted for acetate (**a**), hydrogen (**b**), succinate (**c**), butyrate (**d**), ethanol (**e**), formate (**f**), L-lactate (**g**), and propionate (**h**). Grey boxes refer to missing *pan*-GEMs occurring when the number of available MAGs in the subsets were below 30. Known “fermentation product-to-organism” combinations that have been reported in the literature or the NJC19 database are highlighted in blue.


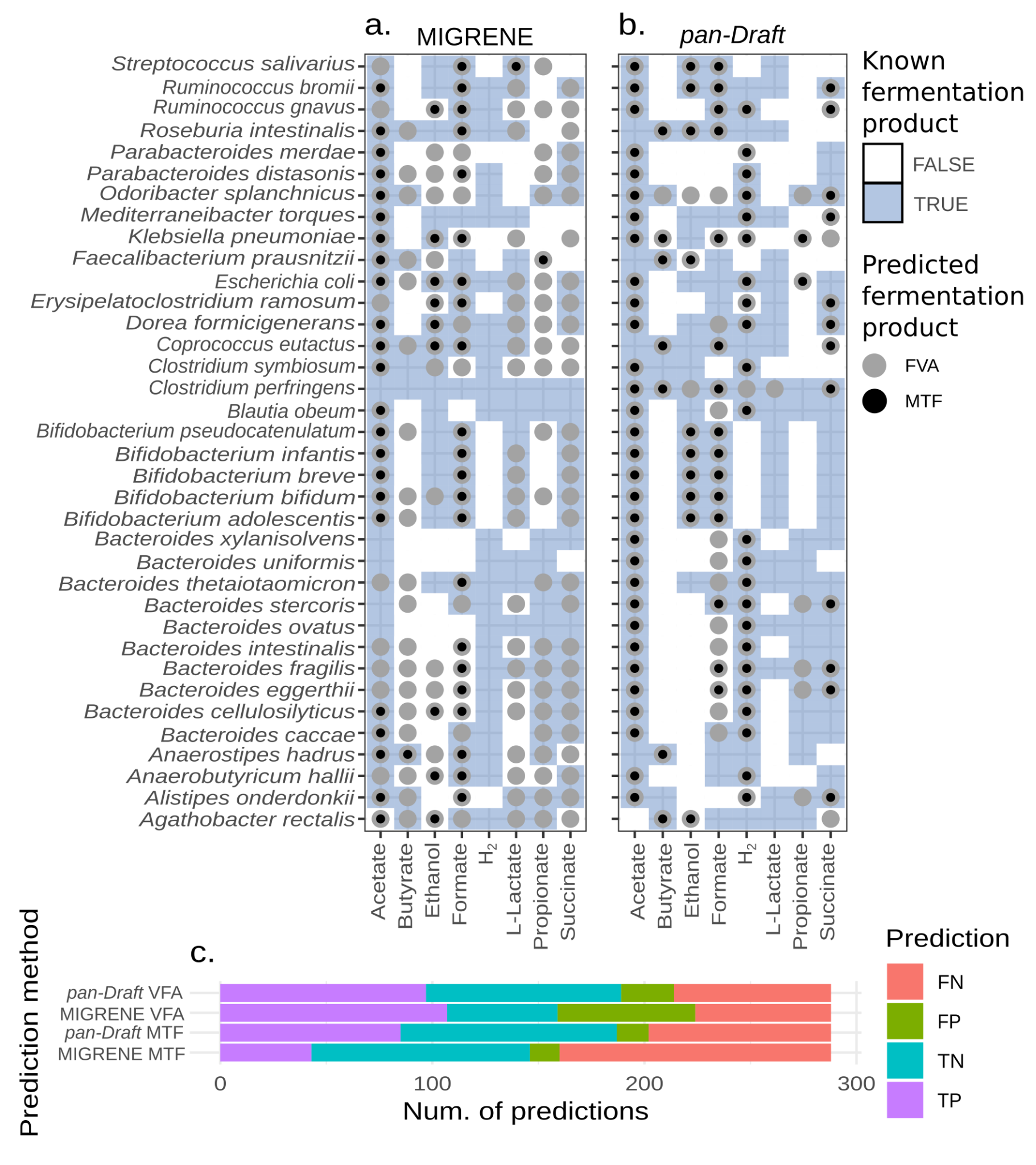


**Fig. S8. Benchmark of *pan-Draft* fermentation product prediction against a state-of-the-art tool.** Results of by-product generation under anaerobic growth for 36 bacteria predicted by MIGRENE (**a**) and *pan-Draft* (**b**). The heatmap background (in blue) indicates known fermentation product metabolites (columns) of the analyzed organisms (rows). The dots represent compounds with a predicted export flux greater than 1e-4 mmol∗gDW^−1^. Predictions were made using two methods: MTF flux balance analysis (in black) and FVA (in gray). The overall prediction performance across all species is summarized depicting the confusion matrix statistics (**c**).

## References

1. Bidkhori G, Shoaie S. MIGRENE: The Toolbox for Microbial and Individualized GEMs, Reactobiome and Community Network Modelling. Metabolites. 2024;14:132.

2. Hyun JC, Monk JM, Palsson BO. Comparative pangenomics: analysis of 12 microbial pathogen pangenomes reveals conserved global structures of genetic and functional diversity. BMC Genomics. 2022;23:7.

3. Tantoso E, Eisenhaber B, Kirsch M, Shitov V, Zhao Z, Eisenhaber F. To kill or to be killed: pangenome analysis of Escherichia coli strains reveals a tailocin specific for pandemic ST131. BMC Biol. 2022;20:146.

4. Shoer S, Reicher L, Pilpel Y, Segal E. Pangenomes of Human Gut Microbiota Uncover Links Between Genetic Diversity and Stress Response. 2024. Preprint at http://biorxiv.org/lookup/doi/10.1101/2024.04.17.589959
