# Supplementary material for: pan-Draft: automated reconstruction of species-representative metabolic models from multiple genomes
Source: Genome Biol. 2024 Oct 25;25:280. doi: 10.1186/s13059-024-03425-1 (PMC11515315; doi:10.1186/s13059-024-03425-1)
Supplement: Supplementary file 3 — Additional file 3. Review history. [file 13059_2024_3425_MOESM3_ESM.docx]

**1^st^ round**

**Reviewer 1**

Please consider the following questions:
-Is the Software likely to be of broad utility? Is it easy to install and use?

Yes, pan-Draft method has been added to the already used gapseq software. I think it can be of broad utility (as explained by the author in the article) with the increase of MAG catalogs, this method expands the quantity of genomes that can be used for metabolic modelling, especially for uncultured organisms.
As a researcher in Bioinformatics and a software developer working on Ubuntu, the software was easy to install and to use. I have only minor comments on the software (listed at the end of the review).

-Does the software represent a significant advance over previously published tools (as demonstrated by direct comparison with available related software)?

The software represents an advance over previously published tools (to my knowledge and as presented in the article). But the article lacks a comparison with methods that it presents itself as limited potential competitor (Microbiome Toolbox and Migrene).

-Please indicate briefly the novel features and/or advantages of the software and/or please reference the relevant publications and which alternatives, if any, it should be compared with.

Pan-Draft introduces a new method that can reconstruct species-level metabolic models from different Metagenome-Assembled Genome (MAGs) by combining models reconstructed through gapseq. In this way, it allows the creation of a metabolic model from sets of incomplete MAGs, which is an improvement compared to the other method presented. It includes a threshold allowing to remove potential contaminant's reaction.

-Are the methods appropriate to the aims of the study, are they well described, and are necessary controls included?

The methods presented are appropriate. They are well described but could required some additions and more definitions. I have listed several comments on this subject.

-Are the conclusions adequately supported by the data shown?

Yes, with the exceptions of few claims and sentences that require some modifications and clarifications.

-Are sufficient details provided to allow replication and comparison with related analyses that may have been performed?

Yes. I replicate easily a small experiment (presented in the part Software review).

-Is the paper of broad interest to others in the field, or of outstanding interest to a broad audience of biologists?

Yes, I think the paper can be of broad interest as incompleteness of MAGs is a recurrent topic and to have the possibility of creation less-biased metabolic model from such data can be very useful (especially with model ready for simulation). But it could require a modification of the paper as it is heavily centered around the validation and could gained from a better description of its result. There is also a need to add definitions of some terms presented as results by the software.

\# Description:

The authors presented a new approach for genome-scale metabolic network reconstruction when dealing with Metagenome-Assembled Genomes (MAGs). MAGs give access to the genomic content of uncultured species but are often incomplete and contaminated. Reconstructing metabolic networks from these incomplete genomes limit the subsequent analysis. This new approach proposed to reconstruct metabolic network from MAGs and cluster them at the species-level to overcome this issue. It is a good addition to the already published and used gapseq method. Thus it is an interesting approach suitable for the Genome Biology journal.
But I have several major comments on the article that I think could make the article difficult to understand for people less accustomed to Bioinformatics. Also, even if the validation is robust to me, I have several comments on it.

\# Major comments:

- (1) There is a lack of description of the results of pan-Draft. The article is heavily focused on validating the approach and its usefulness to solve the issue of incomplete and contaminated MAGs. It is a really good thing but it lacks a presentation of the results made by the method. In the results, the first paragraph 2.1 presents the approach and the datasets used and then the following paragraph 2.2 is directly a validation and how the method works well compared to MAG. For me, between these two paragraphs, it lacks a more descriptive paragraph indicating the direct results of the method.
(1.1) For example: How many metabolic reactions are contained in the pan-GEM model? How many genes are associated with these reactions? What are the repartitions of these reactions between the GEM of the MAGs used to create the pan-GEM (it is explored a little bit after with the MRF threshold)? How many time does it took to reconstruct the MAG GEMs and then use pan-Draft to create the pan-GEMs? In the same vein as the previous question, what is the computing power required to perform such an analysis: personal computer or High Performance Cluster (HPC)?
(1.2) Furthermore, the validation seems robust, but the lack of exploration for the SGBs without reference is impacting the article. Because these are the ones that could benefit form such method as we do not have reference. So I will be interested to see the behavior of the method on them. Is it equivalent to the SGBs with reference genomes or not? What is the impact of the number of MAGs on the prediction, they were made with 30 MAGs but how does the method work with 5, 10 or 20 MAGs?

- (2) There is a lack of comparison to other methods, I have 2 comments on this subject:
(2.1) the authors presented two methods (Microbiome Modeling Toolbox and MIGRENE) that are said to have similar purpose but are limited either to strains contained in AGORA database or in the human gut. As the authors have used a human gut dataset (UHGG), could it be possible to use these methods on some of the SGB that they have tested with pan-Draft to make a benchmark with these tools?
(2.2) The authors use pangenomics terms, so I was wondering if the results could be compared to what is expected from pangenomics analysis. We can expect that core-gene or reaction will be associated with primary metabolism whereas shell or cloud gene/reaction will be associated with more diverse and less conserved functions. To give an example, I was thinking of something similar to the Figure 4 of the article https://www.pnas.org/doi/epdf/10.1073/pnas.1523199113 (showing subsystem distribution according to pan/core reactions). This is linked to my comment on a more descriptive part of of the results, that could help non-Bioinformatics reader to understand the usefulness of the results.

- (3) I have found strange to remove four well know species from this analysis (Escherichia coli, Klebsiella pneumoniae, Clostridium difficile plus another one). I understand the idea but as a validation approach I find it interesting to compare the method to organisms that are highly annotated and known. Especially, what I have found strange is that the authors make a reference to an article showing the benefit of their methods even for high-completed MAG referencing one of E coli (lines 368-382 and reference 39). Technically they could have reproduced this by taking into account E. coli.

- (4) In the paragraph 2.4 (and especially in Figure 5), I do not understand why the isolates were not used in the comparison. Indeed, in the previous paragraph (and Figure 4), the isolates were used in the comparison between pan-GEMs and MAG's GEMs. So I was expecting to also see them in paragraph 2.4 as a ground truth of the species for the fermentation production with FVA and MTF analyses. Is there a specific reason that isolates were not indicated in this paragraph?

- (5) There is a lack of definition for several terms in the Material and Methods section.
(5.1) Multiple terms of pangenomics are used in the article and in the software (such as 'core', 'strictly core', 'shell' or 'cloud'). Defining these terms could help the reading and understanding of the results.
(5.2) In the paragraph 5.5, there is a lack of a definition of how the frequencies of reaction in the pan-GEMs are took into account. For example, if a reaction is present 3 times in a GEMs (due to multiple gene copies), is it taking into account?

- (6) In the Discussion (line 321) the authors claimed that the pan-Draft approach "provides a rapid framework to obtain a holistic perspective". It is right, that in my test (but the author should also include the time run by pan-Draft in their article), pan-Draft is quite fast. But I am quite skeptical of this statement as pan-Draft depends on gapseq which can require up to hours to reconstruct GEM for one organism (it took me 3 hours per MAG on my test dataset, with the 'gapseq doall' command). Then it can take a lot of time and resources to reconstruct a pan-Draft of multiple organisms by running gapseq before.

\# Minor comments:
- Table 1 is confusing, it is difficult to understand what are the SGB that will be processed and analysed. Could another column could be added to show these numbers? Also could the numbers of genomes associated with each number of SGBs could be shown (for example in parenthesis), I think that could help to understand the computational bottleneck of these data, even after filtering steps?
- In Figure 1: in the "Pan-Draft network reconstruction" box, it is written "./gapseq pan-draft" whereas I think it should be "./gapseq pan" (associated with the subcommand of gapseq). Also as it describes both the datasets used and their connections to the workflow, I find that it lacks a box stating that the MAGs were structurally annotated by Pyrodygal.
- Figure 2 is difficult to read. The scatterplot are maybe not the best visualization representation. For the panels a and b, the taxonomy information is not discussed in the text so I think it could be removed. This could ease the reading as it removes the colour. In panels c and d, the colours are not easy to find. Especially the red one (which is discussed in the text). A solution could be to removed the dot of the scatterplot and show only the curve of the fitted generalized additive model for the different phyla. But one scatterplot per taxa could be created and put on a supplemental file to keep these representation and have more complete and readable figures.
- Figure 3: both panels could have the same y-axis scale.
- Lines 41 to 43: "The approach adopted for GEMs reconstruction by some of the most successful tools such as CarveMe, gapseq, and ModelSEED, is based on sequence homology search and gene-protein-reaction rules [6-8]", I am puzzled by this sentence due to several points: (1) by talking of sequence homology search and gene-protein-reaction, there can other methods that can be cited (such as RAVEN, Merlin, AuReMe), (2) due to the importance of gap-filling for CarveMe/gapseq/ModelSEED methods, it should be added to the list of their features and (3) one common element to the three methods the authors cited is that they can reconstruct models ready for simulation. For me, this sentence should ever specify these specific features (gap-filling, generation of model ready for simulation) of these methods or be more general and present other successful methods in GEMs reconstruction.
- Line 133: "The method is applicable to any set of genomes". I have understood that the method has been developed for prokaryotic organisms. But as the method gapseq can be used on eukaryotes (at least its find module), I think it might be helpful to reiterate that the method focuses on prokaryotic genomes. Or maybe am I misunderstanding?
- Lines 141 and 142, as it is the first time that the datasets are presented, it maybe interesting to add the citation to the articles?
- Line 178: I do not understand the association of the sentence with the reference to Table 1. Is there a link that I have missed or is this a typo?
- Lines 189-195: I think there was an exchange between the figure panels in the legend of Figure 2. Indeed, panels (a) and (c) are said to be associated with completeness level whereas on the figure it is the panels (a) and (b). Then the panels (b) and (d) are said to compute the similarity measure whereas it is the panels (c) and (d) that show this on the figure. Finally, on lines 191-192, "On the right panels" refers to the lower panels and not the right ones.
- Line 197: "metabolic model could encompass GEMS", I think the verb include could be clearer in this sentence then encompass.
- Lines 201 and 517: the term core metabolism is used but has not be defined. What is the frequency threshold for a reaction used to define the core metabolism? Because sometimes the term core reflects strictly to genes/reactions occurring in 100% of the considered organisms/networks. But other times, it is associated with extended core with genes/reactions occurring in 90 to 95% of the organisms/networks.
- Lines 291-295: I find the figure "Additional File 1: Fig. S1" difficult to read and interpret. A similar figure was presented in the gapseq article (Figure 3 of https://doi.org/10.1186/s13059-021-02295-1) but as there were less data, the comparison were easier. Here due to the quantity of points, I am not able to say if there are significant differences. And for some metabolites, the models seem to be better than the species-level models. For example, for L-Lactate between panel (a) and (b), it seems to me that the model is a better predictor than the species-level predictor.
- Heterogeneity in the use of "Figure" or "Fig.". As "Fig." is the most use, maybe update the "Figure" in Lines 119, 254, 286 and 513.
- Line 396: "Limnochordia DTU010" is not in italic whereas other name of species or genera are in italic.
- Line 412: the citation [42] corresponds to gapseq article which is already cited as [6].
- Line 474: "In more detail", as there is already the word details used in the previous sentence, I think that the use of "More specifically" could be less redundant.
- Line 497: there is a missing citation for Pyrodigal. I think it is this article: https://joss.theoj.org/papers/10.21105/joss.04296.
- Lines 506-508: The authors state that they are removing oxygen from the media of the UHGG dataset. I suppose that this is due to the fact that human gut is mainly an anaerobic environment, could this be precised here? Furthermore, the authors said in lines 144-146 that around 58% of the metagenomes of the OMD dataset come from epipelagic zone, with aerobic condition, is it possible that some samples from the OMD dataset could come from anoxic waters, then requiring also to remove the oxygen from media?
- Line 551: "species-specific reactome gold standards": even if I understand the use of specific, I find it confusing as the authors are talking about the union of all reactions present the reference genomes of the species, which for me is different from a real "species-specific" set of reactions (which can be viewed as the intersection of the reactions of the species).
- Line 560: there is a missing space in "To determine theMRF parameter".
- Line 569 (and for the whole paragraph 5.6): it is not clear to me about what are the MAGs that were used. I supposed it corresponds to MAGs from bot UHGG and OMD datasets. Could they be specified in the sentence?
- Line 590: "a maximum of 20 different bacterial species (Additional file 2: Table S1)", but when I looked at this file, I count 22 species. Is there two species that were removed?
- Line 640: citation is written as a Systems Biology, whereas the link is associated with BiorXiv.
- Line 698: citation is written as a Systems Biology, whereas the link is associated with BiorXiv.

\# Software review

I have tested pan-Draft in two ways. I have found that gapseq pan-Draft was simple to use and well-documented.
A first test was performed with the toy example present in the gapseq GitHub repository (in the time of the review the most recent commit number was "2d739a5").

A second test was done using a manually selected species ("Desulfovibrio sp910575755") from the MGnify dataset Mouse Gut Genomes v1.0 (https://www.ebi.ac.uk/metagenomics/genome-catalogues/mouse-gut-v1-0). This species had one reference genome from an isolate and 61 MAGs (with completeness ranging from 60 to 98%). I retrieved the genome sequences from MGnify and then use Pyrodigal for the structural annotation of these sequences. Then I ran 'gapseq doall' on the protein sequences to generate the GEMs of the MAGs and the isolate. I used the GEMs of the MAGs as input to gapseq pan-Draft to create a pan-GEM of this species.
After that, I made a simple and little experiment similar to the ones presented in the article. The reactions contained in the GEM of the reference genome were used as reactome gold standard. I computed the F1 score on the presence/absence of reactions between the MAG GEMs and the reactome gold standard and between the pan-GEM and the reactome gold standard (such as in Figure 4). For this species, I found similar F1 scores than the one presented in the article, with the pan-GEM having a better F1 score (0.95) than the median of the MAGs (0.93 with values ranging from 0.59 to 0.99 according to the completeness of the MAGs).

I have several comments/ideas/issues:
- when installing pan-Draft in my environment created for gapseq, it lacks the R package 'tidyr'. Could it be added to the R packages installed with a command line in the documentation of gapseq?
- I used 'gapseq doall' command to create GEMs for MAGs of "Desulfovibrio sp910575755". When I ran "gapseq pan" on the created models, I encountered an issue as it returned me the following error:

"Reading input from specified filename pathway
Error in parse_input_options(pathways.table.path, "-all-Pathways.tbl.gz") :
No files with the specified suffix. Please check the file names.
Execution halted"

After checking, it is due to the lines 114-115 of the script 'gapseq/src/pan-draft.R', that are awaiting files ending with '-all-Pathways.tbl.gz' whereas the files created by 'gapseq doall' end with '-all-Pathways.tbl'.
- Some terms presented in the output of the command line of pan-draft are not presented in the article (such as "shell" or "strict-core"). As I have some knowledge in pangenomics, I understand them. But I think that researchers outside this domain could not know them. So it could be helpful to add definition of these terms in the documentation? Also it could be interesting to feature these terms in the article.
- Except of an error on my side, pan-Draft does not create a draft xml file (compared to 'gapseq doall'). Could it be possible to add the creation of such file (it is easier to use for people in metabolic modeling not fluent with R and the RDS format)?
- For reproducibility, could it be possible to show the version of the database when using "gapseq -v". I was trying to find the version of my sequence database but from what I have understood, it was only possible to find it in the header of some of the result file (such as pathway.tbl). In the same way, could it be possible to have a changelog associated the md5 hash of the sequence DB with its release date, so that user can know if there is a new DB?
- Could it be possible to put the created GEMs (or at least the filtered set of IDs of the experiments) in an open-source data storage such as Zenodo or are they too big?

\# Statistical review comments

The statistical analyses performed in this article correspond to standard analysis performed in the field. The F1 score is regularly used to assess performance. The use of a paired t-test to compare matching MAG's GEM performance and their associated pan-GEMs is also good (but it could be helpful to add a sentence to explain its use in the Material and Methods). I was only wondering for the F1 score computed in Figure 5 if the authors could have used the Matthews Coefficient Correlation instead of the F1 score as they have computed TP/TN/FP and FN. Indeed, the Matthews Coefficient Correlation can be more informative than the F1 score (https://bmcgenomics.biomedcentral.com/articles/10.1186/s12864-019-6413-7).

\# Question

- Pan-Draft was developed around gapseq. But could it be possible that the approach is generalised to work with metabolic networks reconstructed by other methods (such as CarveMe, Pathway Tools, etc)?

**Reviewer 2**

In the manuscript presented, De Bernadini and colleagues present a new tool for generating representative genome-scale metabolic models from multiple metagenomic assembled genomes, leveraging the multiple genomes to identify the core features of a species despite contamination and incompleteness. Such a tool would be beneficial to the microbiology community to more confidently reconstruct the metabolic networks of poorly understood, not yet isolated bacteria, which still compose a large proportion of sequenced taxa. However, we find there to be major issues with respect to the specific motivation and goals behind the design of the tool and the validation of its success at achieving them.
The manuscript presents the fact that such an approach limits the understanding of sub-populations as both a goal (section 2.3) and as a limitation (section 3.3). Similarly, the approach is said to yield a reaction catalog "summarizing strain-level metabolic diversity," and yet the approach by design does not disentangle strain-level differences, in contrast to other pan-genome methods. Additionally, several lines refer to the "solidity" of the model and its results, which does not refer to any specific, measurable goals. Thus, the overall motivation is not presented effectively, making it difficult for the reader to judge the success of the method based on the evidence provided. Lastly, when quantitative methods are used, the written methods lack details and rely heavily on the F1 score, which we show below can produce erroneous results in this context.

Major Comments
The grammar and flow should be thoroughly reviewed, especially the use of commas. While many examples are pointed out below, such issues exist throughout the text.

Fig 5e: I am unsure that these results are so meaningful. If the calculation below is correct, one could get an average F1 score of 0.7 by "predicting" that all metabolites are produced for all species. If we take the data from Additional File 2: Table S1 and "predict" that all metabolites are produced, then each "1" in this data set will be a true positive, each "0" a false positive, and there will be no true or false negatives, as no negative "predictions" were made. This yields the following table, with F1 scores ( 2*TP/(2*TP + FP + FN) ) appended:

[see attached file for the table]

The average F1 score here is 0.701 - higher than the means shown for either the GEMs or the pan-GEMs, with similar minimum and maximum values. Some of this could be an artifact of the metric chosen - the Matthews Correlation Coefficient would give a flat 0 for all of these "predictions," and may be a better choice to demonstrate the benefits of your models. But as it stands, this main validating result is not convincing.
This also raises concerns about the F1 score as a measure of success elsewhere in the manuscript. In particular, as TN is not a variable in the F1 formula, the TN value could change arbitrarily without altering the score. As identifying negatives from non-redundant contamination and rare groups is one of the stated goals, it seems wrong that the metric used does not take this into account.

Minor Comments
Line 27: It is unclear what the authors refer to by "challenges of individual genomes."
Line 77: The acronym MIMAG should be left expanded as it is never used again in the manuscript.
There are several points where "GEMs" is pluralized but perhaps should not be, E.G. lines 41, 57
Lines 54-56: It is unclear how network topology is an example of "additional contextual information" that would be relevant to MAGs in particular over whole genomes.
Line 59: Consider referencing the specific errors from within the citation to provide a more concrete example to the reader.
Lines 63-66: It is unclear how this sentence helps your point. If MAG-derived models correlate well with more traditional models, that makes it sound as though there is less of a need for this new method.
Lines 70-80: I do not see how the problem that "the retrieved best MAGs within a SGB remain susceptible to biases" derives from the proceeding addressed point "many SGBs encompass only metagenomic sequences".
Line 74: Is there any estimation on how many SGBs encompass only metagenomic sequences?
Line 74: Delete the comma after "that"
Line 81: Change to "In recent years,"
Lines 90-92: Wording is unnecessarily complex: Please consider rewording to something like "Using this approach, a model of the opportunistic human fungal pathogen Aspergillus fumigatus was developed from 252 isolates and MAGs to understand the metabolic background of infection versatility."
Line 109: As far as I understand it, a SGB contains sequences grouped based on similarity, but that does not ensure the SGB is monophyletic.
Line 113: Is the solid core network referring to a metabolic network? Consider replacing the word "solid" to more specifically describe how it is intended to describe the term "core network."
Lines 103-115: As pan-Draft relies on gapseq, a brief description of gapseq might be necessary in the introduction. Also, it is unclear to me how pan-Draft differs from available tools to determine core pan-reactome and accessory reactions. If it differs significantly, I suggest comparing it to available tools, such as the Anvi'o anvi-estimate-metabolism function.
Lines 160-162: Why are the criteria for inclusion different for the different datasets?
Lines 162-163: It may be helpful to clarify why you chose the single species you did, and how that avoids the bias in question.
Lines 164-5: It is unclear why these major organisms are being excluded. The pipeline is aimed at less understood species, but would these not be useful for benchmarking your results?
Lines 168-169: It is unclear to me how completeness and contamination was assessed for MAGs in SGBs without reference genomes. As I understand it, a reference is needed to determine both metrics, to know what should or should not be found.
Line 170: The results of this section and the relevant discussion need to be rephrased to more directly answer the question proposed by the section title.
Line 176: Report the percentage of SGBs that fall below 90% completeness.
Lines 177-178: Table 1 does not show information regarding gaps or reactions. Also, this is only true if the missing genes in MAGs tend to be metabolic genes included in GEMs.
Line 181: It would be helpful to quickly mention here that the F1 score is being calculated on reaction presence/absence.
Line 182: The Methods section does not mention how the additive model for statistical purposes was performed.
Line 186: What is "Firmicutes A"?
Lines 274-5: It would help clarify the goals to specify at this point what you mean by "fermentation capabilities," E.G. if you mean the amount produced, the set of products that can be made, etc.
Lines 287-290: It is unclear how this accuracy calculation is being done, especially for the FVA. Is a product considered a model positive if it is produced anywhere in the FVA results? Or if it is always produced? Was the FVA performed at or near the maximum growth rate?
Lines 274-301: The authors must show TP and TN values, rather than only specifying the trends observed (e.g. minimum, maximum, median, etc).
Lines 313-314: How was this 14.6% alignment rate calculated?
Lines 318-320: Using a MRF of 7% to reconstruct species-level GEMs seems to contradict the goal to "assess which reactions are persistent constituents of the metabolism of a taxon" - a reaction being present in only 7% of genomes does not seem to be part of the core set.
Line 400: Change to "low rank taxonomy" or "species level."
Line 445: What is "solidity" in this context?
Line 599: Change to 1e-4 without an exponent. Also correct superscript format in "mmol∗gDW−1"
Lines 559-567: "Minimum reaction frequency" is not defined.
Line 560: Correct "To determine theMRF necessary."
Line 568: Consider rephrasing this header to something that more specifically states what is being found, E.G. "assessing MAGs completeness in quality of species-level GEMs."

Figures
Figure 1: It is unclear what is meant by the red box around reaction prediction, pathway prediction, and draft network reconstruction.

Fig 4b: The F1 score difference legend should be placed next to the heatmap. Currently it appears to be part of 4a.

**Authors’ response**

Reviewer #1

-Is the Software likely to be of broad utility? Is it easy to install and use?

Yes, pan-Draft method has been added to the already used gapseq software. I think it can be of broad utility (as explained by the author in the article) with the increase of MAG catalogs, this method expands the quantity of genomes that can be used for metabolic modelling, especially for uncultured organisms.

As a researcher in Bioinformatics and a software developer working on Ubuntu, the software was easy to install and to use. I have only minor comments on the software (listed at the end of the review).

*The authors express their gratitude to the reviewer for his/her insightful evaluation of the research and appreciate the valuable feedback provided.*

-Does the software represent a significant advance over previously published tools (as demonstrated by direct comparison with available related software)?

The software represents an advance over previously published tools (to my knowledge and as presented in the article). But the article lacks a comparison with methods that it presents itself as limited potential competitor (Microbiome Toolbox and Migrene).

*In response to the reviewer's critique, we have undertaken substantive revisions to address both general and specific comments.*

-Please indicate briefly the novel features and/or advantages of the software and/or please reference the relevant publications and which alternatives, if any, it should be compared with.

Pan-Draft introduces a new method that can reconstruct species-level metabolic models from different Metagenome-Assembled Genome (MAGs) by combining models reconstructed through gapseq. In this way, it allows the creation of a metabolic model from sets of incomplete MAGs, which is an improvement compared to the other method presented. It includes a threshold allowing to remove potential contaminant's reaction.

-Are the methods appropriate to the aims of the study, are they well described, and are necessary controls included?

The methods presented are appropriate. They are well described but could require some additions and more definitions. I have listed several comments on this subject.

*Details of the undertaken revisions are reported below.*

-Are the conclusions adequately supported by the data shown?

Yes, with the exceptions of few claims and sentences that require some modifications and clarifications.

-Are sufficient details provided to allow replication and comparison with related analyses that may have been performed?

Yes. I replicate easily a small experiment (presented in the part Software review).

-Is the paper of broad interest to others in the field, or of outstanding interest to a broad audience of biologists?

Yes, I think the paper can be of broad interest as incompleteness of MAGs is a recurrent topic and to have the possibility of creation less-biased metabolic model from such data can be very useful (especially with model ready for simulation). But it could require a modification of the paper as it is heavily centered around the validation and could gained from a better description of its result. There is also a need to add definitions of some terms presented as results by the software.

*The authors sincerely appreciate the reviewer's positive evaluation and revised the manuscript accordingly. The introduction was refined and previously presented information was clarified to better substantiate our findings.*

\# Description:

The authors presented a new approach for genome-scale metabolic network reconstruction when dealing with Metagenome-Assembled Genomes (MAGs). MAGs give access to the genomic content of uncultured species but are often incomplete and contaminated. Reconstructing metabolic networks from these incomplete genomes limit the subsequent analysis. This new approach proposed to reconstruct metabolic network from MAGs and cluster them at the species-level to overcome this issue. It is a good addition to the already published and used gapseq method. Thus, it is an interesting approach suitable for the Genome Biology journal.

But I have several major comments on the article that I think could make the article difficult to understand for people less accustomed to Bioinformatics. Also, even if the validation is robust to me, I have several comments on it.

*The authors are grateful to the reviewer for the insightful evaluation of panDraft and the valuable feedback, which has significantly enhanced the manuscript's quality. In response to these comments, we have made substantial revisions to address both general and specific points. The details of these changes are outlined below.*

\# Major comments:

**1.** There is a lack of description of the results of pan-Draft. The article is heavily focused on validating the approach and its usefulness to solve the issue of incomplete and contaminated MAGs. It is a really good thing but it lacks a presentation of the results made by the method. In the results, the first paragraph 2.1 presents the approach and the datasets used and then the following paragraph 2.2 is directly a validation and how the method works well compared to MAG. For me, between these two paragraphs, it lacks a more descriptive paragraph indicating the direct results of the method. For example: How many metabolic reactions are contained in the pan-GEM model? How many genes are associated with these reactions? What are the repartitions of these reactions between the GEM of the MAGs used to create the pan-GEM (it is explored a little bit after with the MRF threshold)? How many times does it took to reconstruct the MAG GEMs and then use pan-Draft to create the pan-GEMs? In the same vein as the previous question, what is the computing power required to perform such an analysis: personal computer or High-Performance Cluster (HPC)?

*The authors appreciate the reviewer's suggestion to add a descriptive paragraph to the results section, which has contributed to improving the quality of the revised manuscript. For best text consistency, we split former section 2.3 into two parts and introduced the new descriptive paragraph between them. Thus, the new section titles now read: “2.3. Selection of species pan-reactome”, “2.4. Properties of species-level metabolic models”, and “2.5. Species-level metabolic models show improved network structure” and added a supplementary figure S3. The new section 2.4 can be found on page 15, lines 310-335 of the updated highlighted version of the manuscript.*

“2.3. Selection of species pan-reactome

[...]

2.4. Properties of species-level metabolic models

Having established an optimal pan-reactome selection criterion, we set out to characterize the generated *pan*-GEMs. On average, they possessed 3.1±2.1% more metabolites and 4.0±2.7% more reactions than their counterparts obtained from reference genomes (Fig. 4a-c). Thus, as also visible in Fig. 3, they largely reflected the corresponding iso-GEM in terms of metabolic network components. In contrast, MAG-based GEMs of origin contained up to 64% fewer metabolites and 76% fewer reactions.

The most represented functional super-groups of metabolic reactions pertained to biosynthesis and degradation (Fig. 4d). In particular, core metabolism had numerous reactions for the biosynthesis of lipids, nucleotides, secondary metabolites, and cofactors and for the degradation of carbohydrates (Additional file 1: Fig. S3). Both amino acid biosynthesis and degradation were highly represented pathways. Most of these biomolecules are involved in primary metabolism and have been previously linked with core genome functions by pan-genome analysis [37]. Shell and cloud reactions generally followed the same trends of core reactions, with some exceptions where cloud reactions were over-represented as compared to the core: biosynthesis of storage compounds and polyamines and degradation of fatty acids and lipids, aromatic compounds, and amino acids.”

**Fig. 4. Structural and metabolic features of *pan*-GEMs.** The upper boxplots summarize the number of metabolites (a), the number of reactions (b), and the number of reactions with associated genetic information (c) of MAG-GEMs and *pan*-GEMs normalized by the median values of iso-GEMs. Red dots indicate the statistics for the *pan*-GEMs reconstructed with all the MAGs of an SGB, either in the UHGG (red boxes) and OMD dataset (light blue boxes). The lower boxplot shows the functional classification of reactions grouped into pan-reactome categories by MetaCyc pathways ontology (d). Core, shell, and cloud consist of all reactions with a frequency higher than 95%, between 5% and 95%, and below 5%, respectively, in all the GEMs of any SGB.

**Fig. S3. Pan-reactomic features of *pan*-GEMs.** Classification of reactions grouped into pan-reactome categories by MetaCyc pathways ontology, focusing on the second layer of the metabolic pathways hierarchy under the “Degradation” and “Biosynthesis” classes. Core, shell, and cloud consist of all reactions with a frequency higher than 95%, between 5% and 95%, and below 5%, respectively, in all the GEMs of any SGB.

*The time required to address the reconstruction of pan-GEMs is heavily dependent on the type of analysis undertaken and on the number of MAGs used for SGB. The time used on average to generate a GEM with up to 2 threads is around 2h. Table S1 (in Additional file 1) shows the computational cost of each step averaged over 15,000 model reconstructions, whereas Fig. S1* *shows how the computational cost of pan-Draft scales proportionally to the number of models processed. For this last step the longest run required a single core to process over 4,500 GEMs in ~23 min. These considerations have been recapitulated in the new version of Results section, Page 6, lines 156-160.*

“The computational cost of *pan-Draft* is limited to a few minutes, making it easily applicable to pre-existing GEM collections (Additional file 1: Fig. S1). Nevertheless, the reconstruction of large GEM catalogs remains a demanding process, which limits the use of this method on personal computers for sequence collections including hundreds of MAGs (Additional file 1: Table S1).”

*We also want to underline that improving the speed of gapseq is a priority for the curator of the tool, and efforts will be made to enhance this aspect. An extended explanation of the computational costs is presented in Additional File 1, page 2.*

“The time required for reconstructing *pan*-GEMs varies significantly depending on the number of MAGs involved in species-level model generation. On average, generating a GEM with up to two threads requires about two hours, with the most computationally expensive step being the identification of metabolic pathways (Table S1).

Within this framework, *pan-Draft* had a marginal cost, demonstrating to scale proportionally with the number of models processed (Additional File 1: Fig. S1). Indeed, it required approximately 23 minutes to process more than 4,500 GEMs with a single core. Given the low memory requirements (<2 GB) and the ability to use a single thread per GEM, we believe the process can be efficiently parallelized. This is especially true for reconstructing *pan*-GEMs of environmental communities, which typically include a limited subset of SGBs with more than 30 MAGs, making them manageable on a small workstation. However, generating *pan*-GEMs for model species, such as *Escherichia coli*, which count over 2,000 MAGs in the UHGG database, will likely necessitate high-performance computing. When applying *pan*-Draft on extensively studied species it may be computationally more efficient to select a subsample of the total available MAGs.”

**Table S1. Computational cost of the reconstruction steps*.*** Statistics were estimated for the four *gapseq* modules necessary to generate functional GEMs and averaging results over 15,000 model reconstructions.

| ***Reconstruction step*** | ***Average time*** *(hh:mm:ss)* | ***Standard deviation*** *(hh:mm:ss)* | ***Computed on n model reconstructions*** | ***Num. of used cores*** |
| --- | --- | --- | --- | --- |
| ***gapseq find*** | *01:58:20* | *00:24:40* | *15871* | *2* |
| ***gapseq find_transport*** | *00:06:38* | *00:02:55* | *15924* | *1* |
| ***gapseq draft*** | *00:05:32* | *00:01:59* | *15368* | *1* |
| ***gapseq fill*** | *00:04:30* | *00:00:35* | *14258* | *2* |

**Fig S1. Computational cost of *pan-Draft*.** Relationship between computing time (in seconds) and number of processed GEMs*.*

**1.2** Furthermore, the validation seems robust, but the lack of exploration for the SGBs without reference is impacting the article. Because these are the ones that could benefit from such method as we do not have reference. So I will be interested to see the behavior of the method on them. Is it equivalent to the SGBs with reference genomes or not? What is the impact of the number of MAGs on the prediction, they were made with 30 MAGs but how does the method work with 5, 10 or 20 MAGs?

*We appreciate the reviewer's feedback. However, we believe that the generation of pan-GEMs for SGBs without a reference genome would not contribute to evaluating the performance of pan-Draft. The manuscript aims to show the robustness and validity of the pan-reactome approach both in terms of structural and prediction capacity of the end model. To do so it requires a ground truth, such as reference genomes and well-defined fermentation products. Without this information, it would be difficult to benchmark the performance of the tool. Moreover, we believe that the application of pan-Draft on SGBs with a reference genome is expected to produce analogous results for SGBs without reference since no universal genetic feature is known to distinguish these two classes. Also, we obtained consistent results across distantly related taxonomic groups - and therefore for SGBs with different genetic features - further suggesting that pan-Draft would perform similarly on uncultivated species.*

*Regarding the impact of the number of MAGs on the performance of pan-Draft, this is certainly an interesting point and new experiments were performed to address it. We studied the performance of pan-Draft using sample sizes ranging from two to one thousand MAGs through random subsampling repeated ten times. Our findings indicate that a structural improvement can be achieved with as few as two MAGs, with incremental improvements observed as the number of MAGs increases (see figure below). The new results were included in the updated version of the manuscript in the “2.3 Selection of species pan-reactome” section, page 12, lines 269-294 as follows:*

“Overall, results suggest that a threshold within the range of 5-10% allows the reconstruction of solid *pan*-GEMs for SGBs across different and distant taxonomic groups.

Further validation of the identified optimal threshold was performed by testing MRFs ranging from 0 to 100% on MAG subsamples of variable size. Specifically, MAGs were randomly sampled to form batches of *n* = 2, 5, 10, 15, 30, 60, 100, and 1000 genomes, iterating ten times for each SGB, and the obtained MCCs were averaged over the iterations for a robust characterization. The results confirmed that the optimal MRF lies between 5 and 10% for batches including tens to one thousand MAGs and suggested that it roughly approaches 1/*n* with a decreasing batch size below 15 MAGs (Fig. 3c). This was in part expected given that, with few MAGs, unique differences between genomes become proportionally more frequent and, at the same time, contaminant and accessory reactions get less distinguishable based on their frequency. With *n* MAGs, these reactions typically have a frequency of 1/*n* and the mean optimal MRF lies just below this value, essentially including all the reactions in at least one MAG-GEM (Fig. 3c). As a result, most *pan*-GEMs reconstructed from fewer than 10 MAGs incorporate the union of all reactions in the associated MAG-GEMs. In contrast, when the number of available MAGs is 15 or above, a low MRF threshold is essential to filter out (rarer) contaminant reactions and retain accessory ones.

Such a transition can be further appreciated when observing the effect of five different MRF thresholds on the quality of *pan*-GEMs reconstructed with an increasing number of MAGs (Fig. 3d). The results clearly show a drastic model improvement using few MAGs (2-5) as compared to a single MAG, with the MCC nearing its maximum at 30 MAGs and showing marginal improvements using larger batches. In the extreme case of employing a 0% threshold - i.e. including all the reactions present in any available MAG - the performance is the best achievable one at low MAG numbers, whereas it yields a MCC drop of 0.02±0.02 using 30 MAGs and 0.17±0.06 using 1000 MAGs. Thus, while this permissive approach can be safely used when having genome sequences from isolated microorganisms [34,36], it would lead to a false positive increase of 65.78±41.11 and 424.73±147.63 reactions with 30 and 1000 MAGs, respectively.”

**“Fig. 3. Minimum reaction frequency ~~(MRF)~~ threshold definition for draft *pan*-GEM reconstruction.** Quality of the models generated at the species level with a selected MRF ranging from 0 to 100%. The gray and dark vertical dashed lines highlight the MRF maximizing the MCC ~~F1 score~~ for each SGB and across all the species, respectively. ~~in the UHGG (a) and the OMD (b) dataset, whereas~~. The black solid line depicts the average MCC ~~F1 score~~ across species in the UHGG (a) and the OMD (b) dataset. Boxplots show the relationship between the optimal MRF and the number of MAGs used to reconstruct the draft *pan*-GEM (c), as well as the effect of varying MRF on the quality of the *pan*-GEMs (d). Both boxplots show the distribution across all analyzed SGBs. The results are the median (c) and average (d) of 10 iterations per SGB with random selection of the initial MAGs. The colors in (c) show the median number of MAGs in each SGB corresponding to the selected MRF threshold, computed as: number of MAGs in the subset times the MRF.”

*The Methods section was updated accordingly to include information regarding these new analyses, page 35 lines 759-766 as follows:*

“Additionally, to validate whether the identified threshold is reliable using a varying number of genomes, MAGs were randomly sampled to form batches of 2, 5, 10, 15, 30, 60, 100, and 1000 sequences, iterating ten times for each SGB. The optimal MRF for each SGB was determined by computing the median across iterations of the MRF that maximizes the MCC in each batch. The minimum number of MAGs per SGB used as a threshold to include the reaction in the *pan*-GEM was computed as the number of MAGs in the batch multiplied by the MRF. The effect of varying MRF on the quality of *pan*-GEMs was determined by averaging the MCC across the 10 iterations of each SGB.”

**2.** There is a lack of comparison to other methods, I have 2 comments on this subject:

**2.1** The authors presented two methods (Microbiome Modeling Toolbox and MIGRENE) that are said to have similar purposes but are limited either to strains contained in AGORA database or in the human gut. As the authors have used a human gut dataset (UHGG), could it be possible to use these methods on some of the SGB that they have tested with pan-Draft to make a benchmark with these tools?

*We appreciate the reviewer’s suggestion. As detailed in the following, the Microbiome Modeling Toolbox and MIGRENE deserve separate discussion. Regarding the former, its assumptions and goals are different from those of pan-Draft, as that is based on whole-genome sequences from microbial isolates, while pan-Draft assumes that only MAGs are available. This was clarified in the new version of the manuscript, page 4 lines 111-116, as follows:*

“Recently, new tools were developed to use microbial genomes ~~MAGs~~ in order to reconstruct species-level pan-models independently from manual curation [34,35], such as the “createPanModels” software from the Microbiome Modeling Toolbox 2.0, which is capable of reconstructing pan-models at different taxonomic levels. Unfortunately, this tool is limited to the strains present in the ~~curated databases, such as~~ AGORA collection, which have complete whole-genome sequences [36].”

*The Microbiome Modeling Toolbox thus builds pan-GEMs by considering all the unique reactions present in at least one genome classified at the desired taxonomic level. In our application scenario, this would be equivalent to setting a MRF threshold of 0%. As it can be seen in Fig. 3d, the MCC corresponding to pan-GEMs generated using 30 MAGs would be 93.2±3.2 with an increase of false positives of 65.8±41.1 as compared to the optimal MRF threshold. This consideration was integrated in the new version of the manuscript at lines 288-294, page 13:*

“In the extreme case of employing a 0% threshold - i.e. including all the reactions present in any available MAG - the performance is the best achievable one at low MAG numbers, whereas it yields a MCC drop of 0.02±0.02 using 30 MAGs and 0.17±0.06 using 1000 MAGs. Thus, while this permissive approach can be safely used when having genome sequences from isolated microorganisms [34,36], it would lead to a false positive increase of 65.78±41.11 and 424.73±147.63 reactions with 30 and 1000 MAGs, respectively."

*As regards MIGRENE, new fermentation tests have been performed to benchmark pan-Draft for models matching the analysed gut microbe species. The taxonomic classification allowed us to identify the matching GEMs resulting in 36 species (Additional file 1: Fig. S3). The fermentation capacity was assessed for eight by-products (as in Fig. 6) using pan-GEMs generated by randomly sampling 30 MAGs with a completeness between 90-100% from each SGB. Compared to MIGRENE, pan-Draft demonstrated overall better prediction capacity using both FVA and MTF flux balance analysis, with consistently more TP and TN (Fig. S8c). Specifically, pan-Draft was accurate in predicting the secretion of small molecules such as acetate, butyrate, and ethanol, whereas MIGRENE models resulted in more TP when assessing the potential capacity of the species to secrete succinate, propionate and lactate (Fig. S8a-b). Despite the lower sensitivity of pan-Draft for these last by-products during FVA, the MTF flux balance analysis showed overall higher sensitivity compared to MIGRENE. We invite the reviewer to check the new version of the manuscript to read about the added information, page 20 lines 434-448 as follows:*

“Finally, a comparison against GEMs from the MIGRENE collection was performed to determine whether *pan*-GEMs reached state-of-the-art performances. This test involved *pan*-GEMs from 36 SGBs with species names matching those in the MIGRENE collection and assessing the same fermentation products as before (Additional File 2: Table S3). *pan*-GEMs demonstrated superior performance, showing consistently more correct positive and negative predictions for both FVA and MTF analysis (Additional File 1: Fig. S8c). Specifically, *pan*-GEMs appeared to predict well the secretion of small molecules such as acetate, butyrate, and ethanol but showed low sensitivity for succinate, propionate, and lactate (Additional File 1: Fig. S8b). In contrast, MIGRENE’s models estimated more TPs for these latter metabolites but produced numerous erroneous estimations for hydrogen and butyrate (Additional File 1: Fig. S8a). Although *pan-Draft* showed lower sensitivity for a subset of by-products in FVA, the net MCC improvement was 0.20 and 0.18 for FVA and MTF, respectively. Overall, these results ~~This~~ highlight~~s~~ the capacity of *pan-Draft* to enhance the prediction accuracy of the main fermentation products of representative bacteria of the human gut microbiome during anaerobic growth ~~(Additional File~~ [~~2~~](https://genomebiology.biomedcentral.com/articles/10.1186/s13059-021-02295-1#MOESM2)~~: Table S2)~~.”

*Supplementary methods have been added to specify the differences from the analysis performed in Fig. 6, Additional file 1 - page 2, as follows:*

“1.2 Comparison of fermentation product predictions with a state-of-the-art tool

*pan-Draft* was benchmarked against models in the MIGRENE collection using a strategy similar to the one described in the main text. Species were selected for the analysis only when matching pairs could be identified between the MIGRENE collection and the SGBs analyzed here [1]. Species were matched based on their taxonomy at the species level. This resulted in assessing the production of eight anoxic fermentation products for 36 bacterial species (Additional File 2: Table S3). The fermentation capacity for *pan*-GEMs was assessed by randomly sampling 30 MAGs with a completeness between 90 and 100% from each SGB. A complex growth medium (available in the GitHub repository at dat/media/FT.csv) was employed for both MTF and FVA, and simulations were performed in R (v.4.3.1) using sybil (v.2.2.0) and the CPLEX solver (v.22.11). Like in the tests described in the main text, every metabolite with an outflow flux higher than 1e-4 mmol∗gDW^−1^ was counted as a positive prediction.”

**Fig. S8. Benchmark of pan-Draft fermentation product prediction against a state-of-the-art tool.** Results of by-product generation under anaerobic growth for 36 bacteria predicted by MIGRENE (a) and *pan-Draft* (b). The heatmap background (in blue) indicates known fermentation product metabolites (columns) of the analyzed organisms (rows). The dots represent compounds with a predicted export flux greater than 1e-4 mmol∗gDW^−1^. Predictions were made using two methods: MTF flux balance analysis (in black) and FVA (in gray). The overall prediction performance across all species is summarized depicting the confusion matrix statistics (c).

**2.2** The authors use pangenomics terms, so I was wondering if the results could be compared to what is expected from pangenomics analysis. We can expect that core-gene or reaction will be associated with primary metabolism whereas shell or cloud gene/reaction will be associated with more diverse and less conserved functions. To give an example, I was thinking of something similar to the Figure 4 of the article <https://www.pnas.org/doi/epdf/10.1073/pnas.1523199113> (showing subsystem distribution according to pan/core reactions). This is linked to my comment on a more descriptive part of the results, that could help non-Bioinformatics readers to understand the usefulness of the results.

*We appreciate the suggestion. To get a general overview of the core, shell and cloud reactions, we considered MetaCyc annotations and made two additional figures. The first showing the distribution of reactions based on top ontology level (Fig. 4d) and the second focusing on “biosynthesis” and “degradation” pathways (Fig. S3), which are the most populated classes of the second level. The following paragraph describes the results, which are in agreement with other pan-genomics studies, page 16, lines 326-335:*

“The most represented functional super-groups of metabolic reactions pertained to biosynthesis and degradation (Fig. 4d). In particular, core metabolism had numerous reactions for the biosynthesis of lipids, nucleotides, secondary metabolites, and cofactors and for the degradation of carbohydrates (Additional file 1: Fig. S3). Both amino acid biosynthesis and degradation were highly represented pathways. Most of these biomolecules are involved in primary metabolism and have been previously linked with core genome functions by pan-genome analysis [37]. Shell and cloud reactions generally followed the same trends of core reactions, with some exceptions where cloud reactions were over-represented as compared to the core: biosynthesis of storage compounds and polyamines and degradation of fatty acids and lipids, aromatic compounds, and amino acids.”

**3.** I have found it strange to remove four well known species from this analysis (E*scherichia coli, Klebsiella pneumoniae, Clostridium difficile* plus another one). I understand the idea but as a validation approach I find it interesting to compare the method to organisms that are highly annotated and known. Especially, what I have found strange is that the authors make a reference to an article showing the benefit of their methods even for high-completed MAG referencing one of *E coli* (lines 368-382 and reference 39). Technically they could have reproduced this by taking into account *E. coli*.

*We thank the reviewer for the insightful comment. We acknowledge that there are numerous verifications and comparisons that can be performed to validate our method. Our initial decision to exclude these species was based on the aim to explore the robustness and applicability of our method to a broader range of less-studied microbial genomes. We believed that this approach would provide a more comprehensive assessment of the method’s performance across diverse genomic contexts. That being said, we recognize the validity of your suggestion to include these highly annotated organisms. To comply with your request and to enhance the rigour of our validation process, we have now included all species passing the initial filtering threshold imposed on the minimum number of MAGs and reference genome. Therefore, no extra thresholds on the maximum number of references or SBG per genus were imposed. We have updated the manuscript to incorporate the novel filtering criteria as follows:*

*“5.1 Reference genome and metagenome databases” section, page 31, lines 653-660 as follows:*

“For the OMD dataset, species were included in the analyses if they had at least one reference genome. In contrast, for species in the human gut microbiome, a minimum of 10 genomes was required. ~~To reduce the computational load and focus on phylogenetically distant taxa a single species per genus was selected; if multiple species passed the imposed threshold, only the species with the highest number of available genomes was retained.~~ Again, a minimum number of 30 MAGs per species was required for SGBs to be selected in both datasets~~, excluding those with over 150 reference genomes to reduce the computational load~~.”

*“2.1 Implementation of pan-Draft and dataset structure” section, page 9, lines 200-204 as follows:*

~~Finally, to avoid biases due to an over-representation of specific metabolic groups, a single species per genus was retained. Four species with more than 150 reference genomes were excluded from the analysis being well-known model organisms such as Escherichia coli, or pathogens such as Klebsiella pneumoniae or Clostridium difficile.~~

*We invite the reviewer to review the updated results, which have impacted the "Results” and “Discussion" sections related to Figures 3-6, as well as the section "2. Additional results" in Additional File 1.*

**4.** In the paragraph 2.4 (and especially in Figure 5), I do not understand why the isolates were not used in the comparison. Indeed, in the previous paragraph (and Figure 4), the isolates were used in the comparison between pan-GEMs and MAG's GEMs. So I was expecting to also see them in paragraph 2.4 as a ground truth of the species for the fermentation production with FVA and MTF analyses. Is there a specific reason that isolates were not indicated in this paragraph?

*We thank the reviewer for noticing this. There was no specific reason why the iso-GEMs (i.e., the models for isolates) were excluded. On the contrary, we agree that including the iso-GEMs can be of potential interest for the analysis. We have therefore updated the figure and results description on page 20, lines 413-423 as follows:*

“According to the minimize-total-flux (MTF) analysis and flux variability analysis (FVA), the *pan*-GEMs consistently outperformed ~~standard~~ the GEMs of individual MAGs with completeness levels between 50 and 90% (Fig. 6a, 6b). ~~across all completeness levels (Figure 5). The evaluation involved quantifying the percentage of correctly and incorrectly estimated end-products based on the total number of predictions performed for all the models of a species. The average percentage of true positive (TP) and true negative (TN) metabolites across species demonstrated consistently higher values (Fig. 5a, 5b) in pan-MAGs compared to the models of individual MAGs.~~ In comparison to high quality MAG-GEMs (i.e. completeness levels >90%), the number of correct positive predictions by *pan*-GEMs showed a small decline, but the values remained comparable to those of iso-GEMs.

[...]

Statistically significant differences in MCC ~~F1 score~~ were observed for all MTF and FVA simulations ~~(paired t-Test,~~ *~~P~~*~~<0.05) and for models within the 70 to -80% completeness set, tested via FVA~~ (paired t-test, *P*<0.05 - Additional File [2](https://genomebiology.biomedcentral.com/articles/10.1186/s13059-021-02295-1#MOESM2): Table S4). On the contrary, the differences were not significant for iso-GEMs compared to the other two groups of models sourced by genomes with completeness above 90%.”

**Fig. ~~5~~6. Improvement in fermentation product prediction by the *pan*-GEMs in comparison to ~~the~~ MAG- and iso-GEMs ~~model~~.** Results of the fermentation product test under anaerobic growth obtained with FVA (circles) and MTF flux balance analysis (triangular). Data summarized at the species level (grey symbols) show in percentage the number of TP (**a**), TN (**b**), FP (**c**), and FN (**d**) obtained across all genomes within a species. Similarly, the overall performance is summarized with the MCC ~~F1 score~~ (**e**). Colored dots and error bars show the ~~mean~~ median, ~~and~~ 1^st^ and 3^rd^ quartile ~~standard deviation~~ of all species within the subset obtained according to the MAG completeness level. The number of bacterial organisms tested for each completeness level subset is reported (n. sp.). Statistically significant differences in the MCC distribution, as determined by a paired t-test, are indicated as follows: * P<0.05, ** P<0.01.

**5.** There is a lack of definition for several terms in the Material and Methods section.

**5.1** Multiple terms of pangenomics are used in the article and in the software (such as 'core', 'strictly core', 'shell' or 'cloud'). Defining these terms could help the reading and understanding of the results.

*We thank the reviewer for noticing this. Modifications were implemented into the pan-draft.R script to specify the meaning of the terms: 'core', 'strictly core', 'shell', and 'cloud' present in the output file "pan-reactome_stat.tsv". The updated output format is as follows:*

*nGenome	total_RXN	strict_core (rxn freq. 100%)	core (rxn freq. >95%) shell (rxn freq. 5-95%)	cloud (rxn freq. <5%)*

*1380	2283	149	538	950	646*

*We also included an extended definition of these terms in Additional File 1 since the use of these terms was heavily reduced in the updated version of the manuscript. Their definition can be found at page 1:*

“To facilitate the understanding of pangenomic analysis, the genes in explored sequences are usually classified based on their frequency. The defined categories aim to clarify the distribution and significance of genes and biological functions within a pan-genome, providing insights into both the conserved and variable aspects of a species’ genome. Here, we similarly define key terms to present the features of species-level genome bin (SGB) metabolism, which are used to describe the output of *pan-Draft* and correspond to metabolic reactions in a *pan*-GEM. The terms correspond to the following categories:

- Strict core: this category includes reactions with a frequency of 100%, meaning these reactions are present in all the genomes analyzed.
- Core: this category includes reactions with a frequency above 95%, meaning that these reactions are present in most genomes. It includes reactions that are widespread and relevant for a species' primary metabolic processes. This category includes reactions that could be absent from the strict core category due to the incompleteness of MAGs.
- Shell: this category includes reactions with a frequency between 5% and 95%. These reactions are not universally present across the genomes and may represent functions that are beneficial but not strictly essential.
- Cloud: this category includes reactions with a frequency below 5%, meaning that these reactions are present in only a small subset of genomes. It includes reactions that are often associated with specialized functions, such as environmental adaptation or resistance to specific conditions.”

*Additionally, the term 'core' had been used inappropriately in the previous version of the manuscript and, therefore, it has been substituted. Please refer to our response to the minor comment from the second reviewer titled "28. Lines 318-320" for details on how the term was modified. Here is an example, page 11 lines 247-250:*

“Moreover, correctly predicted reactions (i.e. those actually belonging to ~~the~~ a species pan-reactome) that are present only within small sub-populations ~~are~~ should be preferentially filtered out to generate a draft representative of ~~the~~ a species ~~core~~ metabolism ~~of a species~~.”

**5.2** In the paragraph 5.5, there is a lack of a definition of how the frequencies of reaction in the pan-GEMs are taken into account. For example, if a reaction is present 3 times in a GEMs (due to multiple gene copies), is it taking into account?

*We thank the reviewer for the relevant question. No, the role of redundant genes in a genome is not taken into account since duplicated reactions associated with alternative genes in a GEM are represented by a single reaction. Nevertheless, it is an interesting suggestion for future development of the gapseq software which will be taken into consideration. For clarity reasons we specify this point in the updated version of the manuscript in the section “5.3 pan-Draft metabolic model reconstruction approach”, page 32, lines 684-689 as follows:*

“The aim of this step is to assess which reactions share higher frequency among the models in order to identify the central ~~core~~ metabolism of a species and exclude the rarest functions. Specifically, ~~the~~ reaction presence and absence ~~of reaction and metabolite IDs~~ in/from each of the models are summarized in a binary matrix. It is important to notice that multiple copies of a gene generate a unique identifier in the GEMs, therefore the encoded reaction is counted one single time per model.”

**6.** In the Discussion (line 321) the authors claimed that the pan-Draft approach "provides a rapid framework to obtain a holistic perspective". It is right, that in my test (but the author should also include the time run by pan-Draft in their article), pan-Draft is quite fast. But I am quite skeptical of this statement as pan-Draft depends on gapseq which can require up to hours to reconstruct GEM for one organism (it took me 3 hours per MAG on my test dataset, with the 'gapseq doall' command). Then it can take a lot of time and resources to reconstruct a pan-Draft of multiple organisms by running gapseq before.

*We thank the reviewer for the requested clarification. Indeed, the time required for the generation of the original GEMs is the most computational demanding step of the process. Nevertheless, the generation of the GEMs can be highly parallelized. For instance, using a computer with 30 cores, it is possible to generate up to 180 GEMs within 24 hours. Please refer to the comment 1.1 for a more detailed explanation including newly created Table and Figure. The sentence has been adjusted as follows to remove the unnecessary emphasis on the speed in the updated version of the manuscript, page 23, lines 466-470 as follows:*

“For this reason, the present study introduce~~s~~d *pan-Draft*, a new tool ~~for the automatic reconstruction of draft metabolic models at the species level~~ that integrates an automatic genome-scale model reconstruction pipeline and provides a framework to obtain a holistic perspective on both the potential and variability of unculturable species metabolism.”

\# Minor comments:

**1.** Table 1 is confusing, it is difficult to understand what are the SGB that will be processed and analysed. Could another column be added to show these numbers? Also could the numbers of genomes associated with each number of SGBs be shown (for example in parenthesis), I think that could help to understand the computational bottleneck of these data, even after filtering steps?

*As suggested, information regarding the number of genomes associated with each SGBs were added to Table 1. We agree with the reviewer that this could help clarify the bottleneck of current datasets. Also, during the revision an error affecting most of the table has been identified and corrected. Regarding the number of SGBs analysed in the manuscript, they are 75 and 9 for the UHGG and OMD database, respectively. These two values were marked in Table 1 using “*” in the updated version of the manuscript, page 8, lines 190-194 as follows:*

“**Table 1. Summary of the OMD and UHGG catalog~~s~~ characteristics.** A minimum of ~~1~~ one and ~~10~~ ten isolate/s ~~was~~ were used to filter SGBs with (w/) reference genome/s in the OMD and UHGG catalogs, respectively. In each cell is reported the number of SGBs and the corresponding number of associated genomes. The set of SGBs used in this work is highlighted by an asterisk.

| **Dataset** | **Total genomes** | **Total SGBs** | | **w/ non-redundant sequences, and reference genome/s** | **w/o reference genomes** | **w/o reference genomes, and best MAG completeness level <90%** |
| --- | --- | --- | --- | --- | --- | --- |
| UHGG | 289,232 | 4,744 | ≥30 MAGs | 75*  66345 | 375  56782 | 28  3065 |
|  |  |  | ≥20 MAGs | 75  66345 | 496  59695 | 41  3373 |
|  |  |  | ≥10 MAGs | 78  66453 | 802  63847 | 76  3832 |
| OMD | 34,815 | 8,308 | ≥30 MAGs | 9*  488 | 126  6395 | 59  3089 |
|  |  |  | ≥20 MAGs | 14  611 | 249  9317 | 116  4456 |
|  |  |  | ≥10 MAGs | 25  777 | 611  14181 | 296  6909 |

**2.** In Figure 1: in the "Pan-Draft network reconstruction" box, it is written "./gapseq pan-draft" whereas I think it should be "./gapseq pan" (associated with the subcommand of gapseq). Also as it describes both the datasets used and their connections to the workflow, I find that it lacks a box stating that the MAGs were structurally annotated by Prodygal.

*We thank the reviewer for the comment. Modifications have been implemented as suggested. Please find below an updated version of Figure 1.*

**Fig. 1. Flowchart showing the dataset filtering steps and the pan-Draft workflow.** Set of filtering criteria implemented on the UHGG and OMD dataset to select SGBs with non-redundant genomic information for pan-Draft validation. A minimum threshold of 30 MAGs was imposed to reconstruct a set of draft models through *gapseq*. *pan-Draft* is integrated within the reconstruction pipeline by processing the draft models and the ~~correlated~~ genetic evidence of reactions ~~(highlighted in red) to generate a pan-GEM~~. The information used for each genome of a SGB to generate the *pan*-GEM is highlighted by the red box.

**3.** Figure 2 is difficult to read. The scatterplot are maybe not the best visualization representation. For the panels a and b, the taxonomy information is not discussed in the text so I think it could be removed. This could ease the reading as it removes the colour. In panels c and d, the colours are not easy to find. Especially the red one (which is discussed in the text). A solution could be to removed the dot of the scatterplot and show only the curve of the fitted generalized additive model for the different phyla. But one scatterplot per taxa could be created and put on a supplemental file to keep these representation and have more complete and readable figures.

*We appreciate the reviewer's comments. We have made the suggested modifications to Figure 2 and split the scatterplot per taxa which can now be found in the supplementary materials as Fig. S2* *(Additional File* *1). The new versions of the figures are also provided below together with the corresponding caption in the updated version of the manuscript, page 11, lines 235-242:*

“**Fig. 2. Potential improvement of the species-level metabolic model reconstruction approach.** Completeness level of the representative MAGs for SGBs with no reference genome and at least 30 MAGs in the corresponding database (**a**, **b**). ~~The number of species (s) and MAGs (m) per phylum are reported in the legends on the left.~~ On the lower ~~right~~ panels, a generalized additive model was fitted to the similarity measure of the gapfilled models reconstructed for ~~49~~ 84 SGBs with varying completeness level compared with the species-specific reactome gold standard (c, d). ~~A generalized additive model was fitted to the data to highlight the positive trend.~~ Data regarding the UHGG and the OMD databases are presented in panels (a, c) and (b, d), respectively.”

“**Fig. S2. Comparative analysis of gapfilled metabolic models across phyla for different MAG completeness levels.** Panels depict the Matthews correlation coefficient (MCC) measuring structural similarity between gapfilled GEMs for 84 SGBs and their species-specific reactome gold standard. Results are shown for SGB in both the UHGG and OMD dataset including Cyanobacteria (**a**), Proteobacteria (**b**)**,** Actinobacteriota (**c**), Firmicutes A (**d**), Bacteroidota (**e**), Firmicutes (**f**), and Verrucomicrobiota (**g**).”

**4.** Figure 3: both panels could have the **same y-axis scale**.

*We thank the reviewer for the comment. Modifications have been implemented as suggested. Find below an updated version of the relevant section of Figure 3.*

**5.** Lines 41 to 43: "The approach adopted for GEMs reconstruction by some of the most successful tools such as CarveMe, gapseq, and ModelSEED, is based on sequence homology search and gene-protein-reaction rules [6-8]", I am puzzled by this sentence due to several points: (1) by talking of sequence homology search and gene-protein-reaction, there can other methods that can be cited (such as RAVEN, Merlin, AuReMe), (2) due to the importance of gap-filling for CarveMe/gapseq/ModelSEED methods, it should be added to the list of their features and (3) one common element to the three methods the authors cited is that they can reconstruct models ready for simulation. For me, this sentence should ever specify these specific features (gap-filling, generation of model ready for simulation) of these methods or be more general and present other successful methods in GEMs reconstruction.

*We thank the reviewer for the comment. Indeed, there exist other tools for the automatic reconstruction of GEMs. The aim of this paragraph was to present some representative software and the features relevant to the reconstruction of pan-GEMs from incomplete and contaminated MAGs. Since the approach implemented in pan-Draft is based on comparison of genetic evidence across genomes, we believe it is not strictly needed to introduce here the concept of gapfilling and model ready-to-use. Therefore, for completeness, other tools have been cited as suggested in the updated version of the manuscript, page 1-2, lines 42-44 as follows:*

“The approach adopted for GEMs reconstruction by some of the most successful tools, such as CarveMe, gapseq, RAVEN, Merlin, AuReMe, and ModelSEED, is based on sequence homology search and the definition of gene-protein-reaction rules [6-11]. “

**6.** Line 133: "The method is applicable to any set of genomes". I have understood that the method has been developed for prokaryotic organisms. But as the method gapseq can be used on eukaryotes (at least its find module), I think it might be helpful to reiterate that the method focuses on prokaryotic genomes. Or maybe am I misunderstanding?

*We thank the reviewer for the comment. Indeed, the tool has been developed for prokaryotes, as no eukaryotes were included in the validation set. The sentence has been modified accordingly in the updated version of the manuscript, page 6, lines 153-154 as follows:*

“The method is applicable to any set of prokaryotic genomes, whether they include isolates or MAGs only.”

**7.** Lines 141 and 142, as it is the first time that the datasets are presented, it may be interesting to add the citation to the articles?

*We thank the reviewer for the comment. The references to the databases have been added in the updated version of the manuscript, page 7, lines 174-177 as follows:*

“The UHGG dataset represents a host-~~specific~~associated microbial ~~context~~ domain ~~derived from~~ populating either a strictly anaerobic or microaerobic environment [25]. The second dataset, the Ocean Microbiomics Database (OMD, v1.1), collects 34,815 genomes clustered into 8,308 SGBs [26].”

**8.** Line 178: I do not understand the association of the sentence with the reference to Table 1. Is there a link that I have missed or is this a typo?

*We thank the reviewer for noticing the inconsistency. The reference to Table 1 is indeed related to the results commented in the previous sentence in the updated version of the manuscript, page 10, lines 218-221 as follows:*

“Specifically, OMD had 59 SGBs (0.7% of the total) with maximal MAG completeness levels below 90%, whereas UHGG had ~~38~~ 28 (0.6% of the total - Table 1). Traditional de novo draft GEM reconstruction is expected to yield highly gapped models in these cases ~~(Table 1)~~.”

**9.** Lines 189-195: I think there was an exchange between the figure panels in the legend of Figure 2. Indeed, panels (a) and (c) are said to be associated with completeness level whereas on the figure it is the panels (a) and (b). Then the panels (b) and (d) are said to compute the similarity measure whereas it is the panels (c) and (d) that show this on the figure. Finally, on lines 191-192, "On the right panels" refers to the lower panels and not the right ones.

*We thank the reviewer for noticing the error. The caption of the figure has been corrected. Please refer to minor comment n. 3 for the updated version of the figure and caption.*

**10.** Line 197: "metabolic model could encompass GEMS", I think the verb include could be clearer in this sentence then encompass.

*We thank the reviewer for the comment. The sentence was modified to improve clarity in the updated version of the manuscript, page 11, line 245 as follows:*

“Generating species-level metabolic models could ~~encompass~~ require combining draft

GEMs reconstructed from contaminated MAGs.”

**11.** Lines 201 and 517: the term core metabolism is used but has not be defined. What is the frequency threshold for a reaction used to define the core metabolism? Because sometimes the term core reflects strictly to genes/reactions occurring in 100% of the considered organisms/networks. But other times, it is associated with extended core with genes/reactions occurring in 90 to 95% of the organisms/networks.

*We thank the reviewer for the question. A definition of "core" can be found in additional file 1 under the section: “Definition of pan-reactome terms”. However, we realised that the term "core" is commonly used by the scientific community to represent a proportion of the reactome that does not reflect what a pan-GEM aims to represent. To avoid any confusion, we have substituted the term "core" with more precise adjectives. Please refer to our response to the minor comment from the second reviewer titled “28. Lines 318-320” for details on how the term was modified. Here is an example, page 5, lines 132-134:*

“~~The~~ Cross-comparison of draft models belonging to ~~the~~ a same taxon facilitates the reconstruction of a ~~solid “core”~~ network representative of the majority of the metabolic variability within the cluster.”

**12.** Lines 291-295: I find the figure "Additional File 1: Fig. S1" difficult to read and interpret. A similar figure was presented in the gapseq article (Figure 3 of <https://doi.org/10.1186/s13059-021-02295-1>) but as there were less data, the comparison were easier. Here due to the quantity of points, I am not able to say if there are significant differences. And for some metabolites, the models seem to be better than the species-level models. For example, for L-Lactate between panel (a) and (b), it seems to me that the model is a better predictor than the species-level predictor.

*We thank the reviewer for the valuable comment. Indeed, the level of complexity in Fig. S1 is higher compared to what has been previously shown by J. Zimmermann et al., 2021. The aim of this figure is to provide insight into why the pan-GEMs appeared to perform better than standard models (Fig. 6). In this context, we do not expect the pan-GEMs to constantly outperform the standard models but rather to provide a general improvement. Specifically, the correct predictions of L-Lactate by standard models showed a very low frequency (shown by the size of the dots), indicating that only a small proportion of MAGs encode the required genes for a complete lactic fermentation. In the represented test, the pan-GEMs were generated using only 30 MAGs, so it is likely that MAGs including those essential genes were excluded from the reconstruction subset. Also, despite being low, the MRF threshold imposed by pan-Draft could have discarded low-frequency but essential genes. To improve clarity the figures have been re-drawn to highlight the differences between models for single metabolites prediction. The updated version is included in the supplementary materials (Additional file 1: Fig. S7).*

**Fig. S7. Percentage of MAG-GEMs and *pan*-GEMs predicting the production of tested compounds.** Results of the fermentation product test under anaerobic growth obtained with MTF flux balance analysis for MAG-GEMs, *pan*-GEMs, and iso-GEMs (last column in each heatmap). Species-level models and MAG-GEMs were derived from MAGs with completeness level between 50 to 60% (first and second column) and 90 to 100% (third and fourth column). Point sizes indicate the percentage of models of a species (row) that were predicted to produce a fermentation product metabolite with a rate higher than 1e-4 mmol∗gDW^−1^. Predicted exports are depicted for acetate (**a**), hydrogen (**b**), succinate (**c**), butyrate (**d**), ethanol (**e**), formate (**f**), L-lactate (**g**), and propionate (**h**). Grey boxes refer to missing *pan*-GEMs occurring when the number of available MAGs in the subsets were below 30. Known “fermentation product-to-organism” combinations that have been reported in the literature or the NJC19 database are highlighted in blue.

**13.** Heterogeneity in the use of "Figure" or "Fig.". As "Fig." is the most use, maybe update the "Figure" in Lines 119, 254, 286 and 513.

*We thank the reviewer for noticing it. The suggested modifications were implemented in the updated version of the manuscript:*

*page 6, line 145 as follows:*

"[...] ~~b~~By processing the output of the find, draft and find-transport modules ~~(Figure 1).~~”

*page 18, line 370 as follows:*

"[...] 75~~33~~ SGBs having at least 30 MAGs per completeness interval (~~Figure~~ Fig. ~~4~~5b, Additional file 1: Fig. S4).”

*page 20, lines 414-416 as follows:*

"[...] *pan*-GEMs consistently outperformed ~~standard~~ the GEMs of individual MAGs with completeness levels between 50 and 90% (Fig. 6a, 6b). ~~across all completeness levels (Figure 5)~~.”

“~~Figure~~ Fig. 1 provides an overview of how the pan-Draft module [...]”

**14.** Line 396: "Limnochordia DTU010" is not in italic whereas other name of species or genera are in italic.

*We thank the reviewer for the comment. "Limnochordia" is a* [*class*](https://www.ncbi.nlm.nih.gov/Taxonomy/Browser/wwwtax.cgi?id=1676648) *name, and according to standard taxonomic conventions we italicized only the genus and the species names.*

**15.** Line 412: the citation [42] corresponds to gapseq article which is already cited as [6].

*We thank the reviewer for noticing it. The duplicated reference has been removed.*

[~~42. Zimmermann J, Kaleta C, Waschina S. gapseq: informed prediction of bacterial metabolic pathways and reconstruction of accurate metabolic models. Genome Biol. 2021;22:81.~~](https://www.zotero.org/google-docs/?broken=sQet3W)

**16.** Line 474: "In more detail", as there is already the word details used in the previous sentence, I think that the use of "More specifically" could be less redundant.

*We thank the reviewer for the suggestion. Modified as suggested in the updated version of the manuscript, page 30, line 634 as follows:*

“More specifically ~~In more detail~~, MAGs were initially filtered based on the study and the sample ID.”

**17.** Line 497: there is a missing citation for Pyrodigal. I think it is this article: <https://joss.theoj.org/papers/10.21105/joss.04296>.

*We thank the reviewer for noticing it. The suggested reference has been added in the updated version of the manuscript, page 31, line 6632 as follows:*

 “(i) prediction of proteins encoded in the query genome using pyrodigal (v.2.2.0) [57];”

**18.** Lines 506-508: The authors state that they are removing oxygen from the media of the UHGG dataset. I suppose that this is due to the fact that human gut is mainly an anaerobic environment, could this be precised here? Furthermore, the authors said in lines 144-146 that around 58% of the metagenomes of the OMD dataset come from epipelagic zone, with aerobic condition, is it possible that some samples from the OMD dataset could come from anoxic waters, then requiring also to remove the oxygen from media?

*We thank the reviewer for the comment. Indeed, we chose to remove oxygen from the media because the microbiome colonizing the lumen of the human gut is adapted to extremely low concentrations of oxygen. The logic behind this decision is now included in the sentence in the updated version of the manuscript, pages 31-32, lines 673-694 as follows:*

“Oxygen availability was excluded from the predicted media for MAGs belonging to the UHGG dataset (-c: "cpd00007:0") since species colonizing the lumen of the human gut are adapted to extremely low concentrations of oxygen [58].”

*Regarding the OMD dataset, no strict aerobic or anaerobic conditions were imposed for two reasons. First, most of the samples (58%) derived from the epipelagic zone, which is usually considered rich in oxygen. Therefore, highly represented SGBs, thus species with more than 30 MAGs across samples, are more likely to be aerobic. Second, “gapseq medium” can estimate the need for oxygen of a microorganism based on the presence and absence of specific pathways. For these reasons no strict oxygen limitations were imposed on the OMD SGBs.*

**19.** Line 551: "species-specific reactome gold standards": even if I understand the use of specific, I find it confusing as the authors are talking about the union of all reactions present the reference genomes of the species, which for me is different from a real "species-specific" set of reactions (which can be viewed as the intersection of the reactions of the species).

*We thank the reviewer for the comment. The term "species-specific" indicates that the reactome gold standards represent all reactions found within reference genomes of a species rather than unique reactions specific to that species alone. For clarity all sentences including "species-specific reactome gold standards" were modified. Here an example, page 34, line 727 as follows:*

"species-~~specific~~representative reactome gold standards."

**20.** Line 560: there is a missing space in "To determine theMRF parameter".

*We thank the reviewer for noticing it. The typo was corrected.*

"To determine the MRF necessary [...]"

**21.** Line 569 (and for the whole paragraph 5.6): it is not clear to me about what are the MAGs that were used. I supposed it corresponds to MAGs from both UHGG and OMD datasets. Could they be specified in the sentence?

*We thank the reviewer for required clarification. Unfortunately, no MAGs from the OMD datasets have more than 30 MAGs in more than one single completeness-level subset. Therefore, only species from the UHGG database were included in this and later analysis. To improve clarity the sentence was modified in the updated version of the manuscript, page 36, lines 770-771 as follows:*

“MAGs from the UHGG database were categorized into groups based on five completeness levels, each set at intervals of 10% points from 50% up to 100% (Fig. ~~4~~5).

**22.** Line 590: "a maximum of 20 different bacterial species (Additional file 2: Table S1)", but when I looked at this file, I counted 22 species. Is there two species that were removed?

*We thank the reviewer for the comment. Indeed, two species have been removed because the number of available MAGs in the different subset exceeded 30 MAGs only in the 90 to 100% completeness subset. The table has now been updated (Additional file 2: Table S3), it contains 41 SGBs with caracterization of the fermentation products and more than 30 MAGs in at least one completeness level subset to generate a pan-GEMs. This table was used to plot the updated version of Fig. 6 and the sentence has been clarified in the updated version of the manuscript, pages 36-37, lines 790-792 as follows. Also, in Fig. 6 the maximum value is 40 because within the 90 to 100% completeness level subset a species didn’t have enough MAGs to generate corresponding pan-GEMs.*

“The analysis encompassed ~~8~~ eight common anoxic fermentation products for a maximum of ~~20~~ 40 different bacterial species for each completeness level (Additional file 2: Table ~~S1~~ S3).”

**23.** Line 640: citation is written as a Systems Biology, whereas the link is associated with BiorXiv.

*We thank the reviewer for noticing it. ‘*Systems Biology’ indicated the subject area in *the* [*biorxiv*](https://www.zotero.org/google-docs/?broken=RpJOif) *webs*ite. *To align with the editorial policy the reference was corrected as follows.*

[Faria JP, Liu F, Edirisinghe JN, Gupta N, Seaver SMD, Freiburger AP, et al. ModelSEED v2: High-throughput genome-scale metabolic model reconstruction with enhanced energy biosynthesis pathway prediction ~~[Internet]~~. ~~Systems Biology; 2023 Oct. Available from~~ Preprint at](https://www.zotero.org/google-docs/?broken=hgCMNt)[:](about:blank) <http://biorxiv.org/lookup/doi/10.1101/2023.10.04.556561> ([2023](https://www.zotero.org/google-docs/?broken=cqP5Fh)).

**24.** Line 698: citation is written as a Systems Biology, whereas the link is associated with BiorXiv.

*We thank the reviewer for noticing it. We have modified the previous citation using the new version of the paper recently published in* [*Metabolites*](https://www.zotero.org/google-docs/?broken=wWmUuf)*.*

[Bidkhori G, Shoaie S. MIGRENE: The Toolbox for Microbial and Individualized GEMs, Reactobiome and Community Network Modelling. Metabolites. 2024;14:132.](https://www.zotero.org/google-docs/?broken=W6KKxO)

\# Software review

I have tested pan-Draft in two ways. I have found that gapseq pan-Draft was simple to use and well-documented.

A first test was performed with the toy example present in the gapseq GitHub repository (in the time of the review the most recent commit number was "2d739a5").

A second test was done using a manually selected species ("Desulfovibrio sp910575755") from the MGnify dataset Mouse Gut Genomes v1.0 (<https://www.ebi.ac.uk/metagenomics/genome-catalogues/mouse-gut-v1-0>). This species had one reference genome from an isolate and 61 MAGs (with completeness ranging from 60 to 98%). I retrieved the genome sequences from MGnify and then use Pyrodigal for the structural annotation of these sequences. Then I ran 'gapseq doall' on the protein sequences to generate the GEMs of the MAGs and the isolate. I used the GEMs of the MAGs as input to gapseq pan-Draft to create a pan-GEM of this species.

After that, I made a simple and little experiment similar to the ones presented in the article. The reactions contained in the GEM of the reference genome were used as reactome gold standard. I computed the F1 score on the presence/absence of reactions between the MAG GEMs and the reactome gold standard and between the pan-GEM and the reactome gold standard (such as in Figure 4). For this species, I found similar F1 scores than the one presented in the article, with the pan-GEM having a better F1 score (0.95) than the median of the MAGs (0.93 with values ranging from 0.59 to 0.99 according to the completeness of the MAGs). I have several comments/ideas/issues:

**1.** when installing pan-Draft in my environment created for gapseq, it lacks the R package 'tidyr'. Could it be added to the R packages installed with a command line in the documentation of gapseq?

*We thank the reviewer for their suggestions, which have led to improvements in the software. The 'r-tidyr' dependency has been removed from the list of dependencies in the script “pan-draft.R”. This dependency was a remnant from previous testing of the software and is no longer used in the current version of pan-Draft.*

**2.** I used 'gapseq doall' command to create GEMs for MAGs of "Desulfovibrio sp910575755". When I ran "gapseq pan" on the created models, I encountered an issue as it returned me the following error:

"Reading input from specified filename pathway

Error in parse_input_options(pathways.

table.path, "-all-Pathways.tbl.gz") :

 No files with the specified suffix. Please check the file names.

Execution halted"

After checking, it is due to the lines 114-115 of the script 'gapseq/src/pan-draft.R', that are awaiting files ending with '-all-Pathways.tbl.gz' whereas the files created by 'gapseq doall' end with '-all-Pathways.tbl'.

*We thank the reviewer for noticing it. Since the standard output of ‘gapseq doall’ command are not compressed the ‘pan-draft.R’ script has been modified to accept in input unzip file in the format -all-Pathways.tbl. Also, files of the pan-Draft “./toy” example have been unzip.*

**3.** Some terms presented in the output of the command line of pan-draft are not presented in the article (such as "shell" or "strict-core"). As I have some knowledge in pangenomics, I understand them. But I think that researchers outside this domain could not know them. So it could be helpful to add definition of these terms in the documentation? Also it could be interesting to feature these terms in the article.

*We thank the reviewer for the comment. As suggested in the major comment 5.1, modifications were implemented to specify the meaning of the terms: 'core', 'strictly core', 'shell', and 'cloud' in the output file "pan-reactome_stat.tsv" and in the additional file 1 under the section: “Definition of pan-reactome terms”. The updated output of ‘pan-draft.R’ script now looks like this:*

*nGenome	total_RXN	strict_core (rxn freq. 100%)	core (rxn freq. >95%) shell (rxn freq. 5-95%)	cloud (rxn freq. <5%)*

*1380	2283	149	538	950	646*

*Please check the Additional File 1 or the answer to major comment 5.1 for a more extended definition of these terms.*

**4.** Except of an error on my side, pan-Draft does not create a draft xml file (compared to 'gapseq doall'). Could it be possible to add the creation of such file (it is easier to use for people in metabolic modeling not fluent with R and the RDS format)?

*We thank the reviewer for the comment. According to the suggestion the ‘pan-draft.R’ script has been updated to generate draft pan-GEMs in RDS and SBML (i.e. .xml) format. We invite the reviewer to test the updated toy example running the following command.*

*“./gapseq pan -m toy/M*-draft.RDS -c toy/M*-rxnWeights.RDS -g toy/M*-rxnXgenes.RDS -w toy/M*-all-Pathways.tbl”*

**5.** For reproducibility, could it be possible to show the version of the database when using "gapseq -v". I was trying to find the version of my sequence database but from what I have understood, it was only possible to find it in the header of some of the result file (such as pathway.tbl). In the same way, could it be possible to have a changelog associated the md5 hash of the sequence DB with its release date, so that user can know if there is a new DB?

*We thank the reviewer for the comment. We have modified the output of `gapseq -v` as suggested by the reviewer. The output looks now like this:*

 gapseq version: 1.2 da69219

 Sequence DB md5sum: 17e92c9 (2023-12-12, Bacteria)

 Sequence DB md5sum: 8bb9575 (2023-12-12, Archaea)

*Thank you also for suggesting a changelog for the sequence database. This has been included in the new release of gapseq along with other major improvements (i.e., less dependencies on other R-package such as ‘sybil’,’CHNOSZ’).*

**6.** Could it be possible to put the created GEMs (or at least the filtered set of IDs of the experiments) in an open-source data storage such as Zenodo or are they too big?

*We thank the reviewer for the comment. The GEMs of all the analysed isolates and MAGs, together with the pan-GEMs reconstructed using all available MAGs per SGB have been uploaded to* <https://doi.org/10.5281/zenodo.12806931>. *Moreover, a list of the reference genome of each SGB passing the filtering process has been added in Additional file 2 - Table S1 and all the corresponding IDs of genomes in the filtered SGBs were added in Table S2. The “Availability of data and materials” has been modified to indicate this information, page 38, lines 825-829 as follows:*

“The GEMs generated for all the analysed isolates and MAGs, together with the *pan*-GEMs reconstructed using all available MAGs in a SGB, have been made available at <https://doi.org/10.5281/zenodo.12806931>. The provided models are in SBML format and have been gapfilled on the predicted minimal medium (./gapseq medium).”

\# Statistical review comments

The statistical analyses performed in this article correspond to standard analysis performed in the field. The F1 score is regularly used to assess performance. The use of a paired t-test to compare matching MAG's GEM performance and their associated pan-GEMs is also good (but it could be helpful to add a sentence to explain its use in the Material and Methods). I was only wondering for the F1 score computed in Figure 5 if the authors could have used the Matthews Coefficient Correlation instead of the F1 score as they have computed TP/TN/FP and FN. Indeed, the Matthews Coefficient Correlation can be more informative than the F1 score (<https://bmcgenomics.biomedcentral.com/articles/10.1186/s12864-019-6413-7>).

*We thank the reviewer for the comment. As explained in the response to the major comment of the second reviewer, we implemented the suggested MCC metric to quantify the structural and functional similarity between pan-GEMs and the other GEMs. The MCC was used in the new version of Fig. 2-3, 5-6. Also, Material and Methods were modified to clarify how the t-test was implemented in the updated version of the manuscript, pages 37-38, lines 814-817 as follows:*

“The degree of improvement between species-level models and models derived from individual MAGs was ~~tested~~ assessed with a paired t-test (R lib. stats, v.4.0.3). The test was implemented by comparing the MCC performance obtained by MAG-GEMs and the corresponding pan-GEMs. This was repeated independently for the MTF and the FVA results and for each MAG completeness level.”

\# Question

- Pan-Draft was developed around gapseq. But could it be possible that the approach is generalised to work with metabolic networks reconstructed by other methods (such as CarveMe, Pathway Tools, etc)?

*The logic behind pan-Draft is certainly generalizable to other tools that are based on similarity search such as* CarveMe *and* Pathway Tools. *However, implementing this approach would require significant modifications to the software and the creation of custom functions to handle the output. This is particularly challenging due to the differences between various tools. For example, CarveMe differs from gapseq in several key aspects, including the programming language used (Python instead of R) and the reaction nomenclature system employed (BiGG instead of ModelSeed). As other readers might have the same question, we have explicitly mentioned the generalizability of the approach in the manuscript on page 29, lines 617-619:*

“Furthermore, the tool provides a foundation for versatile pan-reactome analysis at different taxonomic levels and its logic lends itself to being generalized to other GEM reconstruction tools.”

Reviewer #2

In the manuscript presented, De Bernadini and colleagues present a new tool for generating representative genome-scale metabolic models from multiple metagenomic assembled genomes, leveraging the multiple genomes to identify the core features of a species despite contamination and incompleteness. Such a tool would be beneficial to the microbiology community to more confidently reconstruct the metabolic networks of poorly understood, not yet isolated bacteria, which still compose a large proportion of sequenced taxa. However, we find there to be major issues with respect to the specific motivation and goals behind the design of the tool and the validation of its success at achieving them.

The manuscript presents the fact that such an approach limits the understanding of sub-populations as both a goal (section 2.3) and as a limitation (section 3.3). Similarly, the approach is said to yield a reaction catalog "summarizing strain-level metabolic diversity," and yet the approach by design does not disentangle strain-level differences, in contrast to other pan-genome methods. Additionally, several lines refer to the "solidity" of the model and its results, which does not refer to any specific, measurable goals. Thus, the overall motivation is not presented effectively, making it difficult for the reader to judge the success of the method based on the evidence provided. Lastly, when quantitative methods are used, the written methods lack details and rely heavily on the F1 score, which we show below can produce erroneous results in this context.

*The authors thank the reviewer for the insightful evaluation and valuable feedback. In response, we have made substantial revisions to address both the general and specific comments provided.*

Major Comments

**1.** The grammar and flow should be thoroughly reviewed, especially the use of commas. While many examples are pointed out below, such issues exist throughout the text.

*We are thankful for the feedback. We have thoroughly reviewed the grammar and flow of the manuscript, especially the use of commas. As it can be seen in the following answers and in the marked-up version of the manuscript, the text has been refined and grammar and punctuation have been carefully checked throughout the manuscript. We provide a couple of examples below.*

*Page 1, lines 37-42:*

“Through GEM application, recent studies have solved many open questions that ~~which~~ were previously impossible to tackle~~; for~~. For instance, they disclosed microbial dynamics based on amino acid auxotrophies in a complex microbiome [1], identified disease-specific marker molecules in the human gut [2], and ~~demonstrated~~ proposed ecological roles and industrial ~~roles~~ applications of species that cannot be isolated [3–5].”

*Pages 23-24, lines 480-484:*

“[...] *pan-Draft* does not require context-specific reference models or an extended gene catalog~~,~~ and leverages gene-protein-reaction annotation rules of multiple datasets (i.e. MetaCyc [39], KEGG [40], and ModelSEED [41]). This allows an easier application of ~~pan-based~~ metabolic network reconstructions outside the scope of highly studied environments~~,~~ such as the human gut microbiome.”

**2.** Fig 5e: I am unsure that these results are so meaningful. If the calculation below is correct, one could get an average F1 score of 0.7 by "predicting" that all metabolites are produced for all species. If we take the data from Additional File 2: Table S1 and "predict" that all metabolites are produced, then each "1" in this data set will be a true positive, each "0" a false positive, and there will be no true or false negatives, as no negative "predictions" were made. This yields the following table, with F1 scores ( 2*TP/(2*TP + FP + FN)) appended: [see attached file for the table]

The average F1 score here is 0.701 - higher than the means shown for either the GEMs or the pan-GEMs, with similar minimum and maximum values. Some of this could be an artifact of the metric chosen - the Matthews Correlation Coefficient would give a flat 0 for all of these "predictions," and may be a better choice to demonstrate the benefits of your models. But as it stands, this main validating result is not convincing.

This also raises concerns about the F1 score as a measure of success elsewhere in the manuscript. In particular, as TN is not a variable in the F1 formula, the TN value could change arbitrarily without altering the score. As identifying negatives from non-redundant contamination and rare groups is one of the stated goals, it seems wrong that the metric used does not take this into account.

*We thank the reviewer for the comment. We understand the concern, however we would like to highlight that we aim to demonstrate that pan-GEMs perform better than GEMs obtained from individual (and most probably incomplete) MAGs. For this reason, we are not focusing on the overall value of the F1 score but rather on the F1 score difference between the two categories. In Fig. 6a-d, it can be seen that the individual metrics of the pan-GEMs consistently show higher performance in TP and TN and lower in FP and FN, suggesting that the measured improvement was not an artefact of the selected metrics. Thus, while the provided “naive prediction” example is relevant to model reconstruction tools in general, we believe it be less so in our case.*

*Nevertheless, we acknowledge that the MCC could be a better choice as it takes class imbalance into account. We have therefore implemented the suggested MCC metric in place of the F1 score throughout the manuscript to quantify the structural and functional similarity between pan-GEMs and the other GEMs. In the context of structural similarity, the definition of a negative reaction set was introduced to describe the reactions not belonging to the pan-reactome of a species. To define this set for all the SGBs, all the 7333 ModelSEED reactions used by gapseq were identified (retrievable in the “dbhit” column of the “-all-Reactions.tbl.gz” table). These reactions were further filtered by selecting only those annotated as “approved” or “corrected” in the list available in the GitHub repository at ./dat/seed_reactions_corrected.tsv. After filtration, 3760 reactions represent the full set of reactions that could potentially be included in a bacterial GEM. For each SGB, the negative set was eventually defined as the full set minus the pan-reactome of the isolates (i.e., the species-representative gold standard). The new results using MCC are analogous to those obtained previously with the F1 score and confirm the structural and functional improvement of pan-GEMs over MAG-GEMs.*

*We invite the reviewer to look at the updated version of Figures 2-3, 5-6 and the corresponding new section of the manuscript which underwent an extended revision and updating process. Below are reported modifications included in the “Method” section. Page 34, lines 723-743:*

“The quality of the reconstructed metabolic models was assessed through pairwise network comparisons. The reactions encoded by genes in the reference genomes were aggregated to define the full metabolic potential of a microbial species. The resulting lists of reactions from this process will be referred to as species-specific reactome gold standards. Simultaneously, we also defined the universe of available reactions to generate a bacterial model with *gapseq.* This was done by parsing the "dbhit" column of the “-all-Reactions.tbl.gz” table which identifies pathway predictions in bacterial species mapping to the *gapseq* reaction database. The resulting 7,333 reactions were filtered by selecting only those annotated as “approved” or “corrected” in the ./dat/seed_reactions_corrected.tsv table, available in the GitHub repository. After filtration, 3,760 reactions represented the reaction universe used by *gapseq* to build bacterial GEM. This filtered list was then compared to the species-specific reactome gold standards to define the negative reaction set of each SGB, which includes biochemical transformation outside the metabolic boundaries of the species.

Subsequently, the reactomes of the MAGs were derived from their corresponding GEMs and stored as a binary matrix indicating the presence or absence of reactions in each model (rxn2mod.tsv). To assess accuracy, the MCC ~~F1 score~~ was computed ~~to compare~~ by comparing each column of the matrix with the reactome gold standard expressed in a consistent binary form (Fig. 2c, d). This same metric was applied to evaluate the quality of the reconstructed *pan*-GEMs (Fig. 3, 5), with the MCC formula ~~used was~~ defined according to Equation 1.

MCC=TN x TP - FN x FP(TP + FP) (TP + FN)(TN + FP)(TN + FN) (1)

~~The same metric was used to quantify the quality of the reconstructed pan-GEMs (Fig. 3, 4)~~.”

Minor Comments

**1.** Line 27: It is unclear what the authors refer to by "challenges of individual genomes."

*We thank the reviewer for requesting clarification. These challenges refer to the issues related to the incompleteness and contamination of MAGs. We have revised the sentence for clarity in the updated version of the manuscript, page 1, lines 27-29 as follows:*

“By comparing MAGs clustered at the species~~-~~ level, *pan-Draft* addresses ~~challenges~~ the issues due to the incompleteness and contamination of individual genomes, providing high-quality draft models and an accessory reactions catalog supporting the gapfilling step.”

**2.** Line 77: The acronym MIMAG should be left expanded as it is never used again in the manuscript.

There are several points where "GEMs" is pluralized but perhaps should not be, E.G. lines 41, 57

*We thank the reviewer for the comments. The acronym MIMAG was not expanded as the Minimum Information about a Metagenome-Assembled Genome is now widely recognized as an international standard in the field. The suggested correction for GEM has been implemented in all instances in the updated version of the manuscript. One example is at page 1, line 42:*

 “The approach adopted for GEM~~s~~ reconstruction by some of the most successful tools, such as […]”

**3.** Lines 54-56: It is unclear how network topology is an example of "additional contextual information" that would be relevant to MAGs in particular over whole genomes.

*We thank the reviewer for the comments. In this context, "contextual information" refers to additional data and knowledge that go beyond the basic genomic sequence information of a single genome. Network topology refers to the structure and organization of metabolic networks, which can inform gapfilling methods by highlighting essential pathways and connections that need to be preserved or established for a functional model. This type of information is generally not considered during model generation and gapfilling by software like gapseq and CarveMe. By incorporating such contextual information, more precise and biologically relevant metabolic models can be constructed, enhancing our understanding of the metabolic capabilities of MAGs. For these reasons we believe network information to be a relevant example and a sentence was added in the updated version of the manuscript, page 2, lines 57-65:*

“Consequently, developing a precise ~~metabolic model~~GEM reconstruction strategy ~~for~~ from MAGs requires innovative frameworks that go beyond the annotation of single genomes and can take advantage of ~~by leveraging~~ additional contextual information. Examples of ~~such~~ relevant modelling approaches include ~~constrained~~ gapfilling methods that limit the reaction universe based on taxonomic information~~, or~~ and topology-based gapfilling methods ~~guided by~~ that leverage high-order network properties to train machine learning models and resolve non-trivial inconsistencies ~~topology~~ [12,13]. By drawing relevant information from taxonomically or topologically related organisms, gaps arising from MAG incompleteness can be resolved.”

**4.** Line 59: Consider referencing the specific errors from within the citation to provide a more concrete example to the reader.

*We thank the reviewer for the comment. An example of a simple error that could arise in a GEM generated from an incomplete MAG would be the impossibility to reproduce production of a given secondary metabolite due to the lack of genes categorised in the relevant module.*

*A more descriptive sentence was added to the text in the updated version of the manuscript, pages 2-3, lines 66-70.*

“Neglecting the relevance of MAG~~s'~~ completeness level in GEM~~s~~ reconstruction may result in erroneous prediction of metabolic capabilities.~~, such as~~ For instance, misinterpreting an artifactual deficit of functions as a genuine biological signal could occur when considering modules in the ‘nucleotide metabolism’ and 'biosynthesis of other secondary metabolites' domains, which have been found to be most affected by genome incompleteness [18].”

**5.** Lines 63-66: It is unclear how this sentence helps your point. If MAG-derived models correlate well with more traditional models, that makes it sound as though there is less of a need for this new method.

*We thank the reviewer for the comment. The need for this method arises from the fact that current metagenomics approaches often fail to retrieve complete MAGs for some species. As shown in Fig. 2, some frequently detected species in the UHGG and OMD databases consistently result in incomplete MAGs (less than 90% completeness for the “best” MAG). Furthermore, a previous comparison of highly complete MAGs (approximately 95%) with isolated genomes of E. coli revealed that MAGs correctly captured only 77% of the core genes and 50% of the variable genes within the population ([Meziti et al.,](https://www.zotero.org/google-docs/?broken=TsOQGf) 2021). This suggests that current methods for assessing MAG quality do not always provide reliable scores. In this context, the pan-Draft approach can help address the challenges of GEM reconstruction from incomplete genomes by leveraging additional information given by a multitude of MAGs. The corresponding sentence was modified to be clearer in the updated version of the manuscript, page 3, lines 72-80:*

“In general, GEMs derived from low-quality MAGs have limited ability in representing reliable metabolic capabilities [19]. Additionally, ~~comparing~~ although there is a positive correlation between MAG completeness and the structural integrity of MAG~~s~~-derived metabolic models ~~and reference-derived with models derived from high-quality reference genomes (e.g. from isolated species) revealed a noteworthy positive correlation between the network structural similarity and the completeness level~~ [6], highly complete MAGs may fail to capture core genes [20]. As a consequence, there is a pressing need for novel reconstruction approaches that consider biases induced by technical limitations of the metagenomic approach.”

**6.** Lines 70-80: I do not see how the problem that "the retrieved best MAGs within a SGB remain susceptible to biases" derives from the proceeding addressed point "many SGBs encompass only metagenomic sequences".

*The mentioned section was revised for greater clarity in the updated version of the manuscript, pages 3-4, lines 86-94:*

“However, since a large fraction of the species living in the natural environment are yet to be isolated, ~~It is important to note that, while some SGBs may contain both reference genomes and MAGs,~~ many SGBs include only ~~encompass solely~~ metagenomic sequences~~. This happens because a large fraction of the species living in the natural environment are yet to be isolated~~ [21,22]. As a result, for these species similarity search can, at best, identify MAGs ~~with~~ of high~~er~~ quality according to MIMAG standards [24]. ~~However~~Thus, ~~despite the improved genome completion,~~ in these cases the retrieved best MAGs within a SGB remain susceptible to ~~biases~~incompleteness, thereby limiting the interpretability ~~of~~ in related analysis.”

**7.** Line 74: Is there any estimation on how many SGBs encompass only metagenomic sequences?

*We thank the reviewer for the required clarification. Yes, the second-to-last column of "Table 1. Summary of OMD and UHGG catalogs characteristics" in the original version of the manuscript shows the number of SGBs with a varying minimum of MAGs and no reference genome in the two datasets. A comment was added to better clarify this aspect in the updated version of the manuscript, page 8, lines 184-187:*

“Notably, the majority of these SGBs lack an isolated representative (see columns 5-6 in Table 1), highlighting both the gap existing between cultured and uncultured microorganisms and the exploitable genomic redundancy for uncultured taxa.”

**8.** Line 74: Delete the comma after "that"

*To simplify the syntax, the sentence has been rephrased as follows. Page 3-4, lines 86-94:*

“However, since a large fraction of the species living in the natural environment are yet to be isolated, ~~It is important to note that, while some SGBs may contain both reference genomes and MAGs,~~ many SGBs include only ~~encompass solely~~ metagenomic sequences”

**9.** Line 81: Change to "In recent years,"

*Done as suggested (page 4, line 95).*

“In ~~the last~~ recent years, multiple efforts have tried to organize [...]”

**10.** Lines 90-92: Wording is unnecessarily complex: Please consider rewording to something like "Using this approach, a model of the opportunistic human fungal pathogen Aspergillus fumigatus was developed from 252 isolates and MAGs to understand the metabolic background of infection versatility."

*Done as suggested. The mentioned section was removed in the updated version of the manuscript, page 4, lines 105-109:*

“~~Utilizing 252 isolates and MAGs, the metabolic model of Aspergillus fumigatus, an opportunistic human fungal pathogen, was curated to understand the metabolic background of infection versatility~~ [~~[29]~~](https://www.zotero.org/google-docs/?broken=xyEN7e)~~.~~ For example, a model of the opportunistic human fungal pathogen *Aspergillus fumigatus* was developed from 252 isolates and MAGs to understand the metabolic background of infection versatility."

**11.** Line 109: As far as I understand it, a SGB contains sequences grouped based on similarity, but that does not ensure the SGB is monophyletic.

*We thank the reviewer for the observation. Indeed, the genetic relatedness between sequences is a good proxy for clustering of MAGs belonging to the same species, nevertheless it does not guarantee the SGB is monophyletic. The sentence was modified accordingly in the updated version of the manuscript, page 5, lines 127-130:*

“Specifically, through pan-reactome analysis~~,~~ the frequency of non-redundant metabolic reactions encoded in the genomes of a SGB can be quantified, thereby effectively characterizing the whole enzymatic capacity of ~~the monophyletic~~ a group of sequences.”

**12.** Line 113: Is the solid core network referring to a metabolic network? Consider replacing the word "solid" to more specifically describe how it is intended to describe the term "core network."

*We thank the reviewer for the required clarification. The sentence was modified in the updated version of the manuscript, page 5, lines 132-134, to improve clarity on both the term “solid” and “core”.*

“~~The~~ Cross-comparison of draft models belonging to ~~the~~ a same taxon facilitates the reconstruction of a ~~solid “core”~~ network representative of the majority of the metabolic variability within the cluster.”

**13.** Lines 103-115: As pan-Draft relies on gapseq, a brief description of gapseq might be necessary in the introduction. Also, it is unclear to me how pan-Draft differs from available tools to determine core pan-reactome and accessory reactions. If it differs significantly, I suggest comparing it to available tools, such as the Anvi'o anvi-estimate-metabolism function.

*We thank the reviewer for the comment. A brief description of gapseq has been added at the beginning of the results section, in “2.1 Implementation of pan-Draft and dataset structure”, page 5, lines 140-153 (see below). Additionally, tools such as Anvi'o anvi-estimate-metabolism function rely on KEGG annotations to estimate the pathway completeness in a genome and to determine the metabolic potential of the species. In contrast, pan-Draft was developed to assess the frequency of reactions in a set of GEMs independently from pathway annotation data. Therefore, we believe the suggested tool is not directly comparable with pan-Draft, and for this reason, a comparison was not implemented.*

“The *pan-Draft* module is integrated within ~~the~~ *gapseq*, a bioinformatics pipeline designed for the automated de-novo reconstruction of ready-to-use GEMs. Given a genomic sequence, the pipeline uses two modules (i.e. *find* and *find-transport*) to perform a sequence similarity search against databases collecting gene-protein-reaction annotation rules and a third module (i.e. *draft*) to reconstruct a preliminary metabolic network [6]. ~~b~~By processing the output of the *find*, *draft* and *find-transport* modules *pan-Draft* aims ~~(Figure 1). The main target of the pan-Draft module is~~ to reconstruct high-quality draft metabolic models from several ~~(potentially complementary)~~ incomplete MAGs having a potentially complementary gene content through a comparative pan-reactome analysis (Fig. 1). Briefly, ~~the tool performs~~ a network analysis is performed using a minimum reaction frequency (MRF) threshold to generate a species-level draft model. Simultaneously, ~~it assesses the species metabolic spectrum by weighting the reactions present in the input MAGs by frequency to adjust reaction weights for further gapfilling~~ reaction weights, used in the gapfilling step, are defined depending on respective reaction frequencies (further details in the Methods section).”

**14.** Lines 160-162: Why are the criteria for inclusion different for the different datasets?

*We thank the reviewer for the required clarification. As introduced in the “Background” section and further detailed in “2.1 Implementation of pan-Draft and dataset structure”, the difference in inclusion criteria is due to the distinct characteristics of the two datasets. Specifically, the filtering difference stands in the higher number of reference genomes per SGB in the UHGG dataset as compared to the OMD dataset. UHGG represents the microbial community of a relatively simple environment, with a number of species per sample typically around 100 that has been deeply analysed in the literature and target for several microbial isolation studies due to its high medical relevance. These extensive studies led to the isolation of the same species with a higher frequency than in the OMD, which represents a much more complex and diverse microbiome subject to a wide spectrum of environmental parameters and diverse ecological conditions.*

*To validate our approach, the optimal scenario would involve having SGBs with a high number of both reference genomes and MAGs to verify whether the pan-GEMs can accurately capture the genetic variability of the species. Unfortunately, due to limitations in the current datasets availability, looser thresholds had to be imposed for the OMD dataset because no species had a large number of MAGs and at least 10 isolates.*

**15.** Lines 162-163: It may be helpful to clarify why you chose the single species you did, and how that avoids the bias in question.

*We thank the reviewer for this comment. To take advantage of the larger number of available species and further validate our initial findings, the revised version of the manuscript includes all species that passed the filtering step. As a result, multiple species within the same genus were not excluded. The description of the filtering strategy has been updated accordingly by removing the following sections:*

*“2.1 Implementation of pan-Draft and dataset structure” section:*

~~Finally, to avoid biases due to an over-representation of specific metabolic groups, a single species per genus was retained. Four species with more than 150 reference genomes were excluded from the analysis being well-known model organisms such as Escherichia coli, or pathogens such as Klebsiella pneumoniae or Clostridium difficile.~~

*“5.1 Reference genome and metagenome databases” section:*

~~To reduce the computational load and focus on phylogenetically distant taxa a single species per genus was selected; if multiple species passed the imposed threshold, only the species with the highest number of available genomes was retained.~~

**16.** Lines 164-5: It is unclear why these major organisms are being excluded. The pipeline is aimed at less understood species, but would these not be useful for benchmarking your results?

*We thank the reviewer for the comment. These organisms were excluded since an excessive number of genomes were present in the database compared to the average availability for other species, thus were initially considered as not representative of common application scenarios. However, following reviewer #1’s suggestion, we decided to build the ~10.000 model for the missing species in order to include them in the benchmark analysis. Please refer to the new version of the manuscript as reported above (response #3, reviewer #1) to see the multiple changes done accordingly.*

**17.** Lines 168-169: It is unclear to me how completeness and contamination was assessed for MAGs in SGBs without reference genomes. As I understand it, a reference is needed to determine both metrics, to know what should or should not be found.

*We appreciate the reviewer’s concern regarding the assessment of completeness and contamination. To estimate them, we used CheckM as this is the most widely recognised and applied method in the field. The tool is specifically designed for assessing the quality of MAGs based on lineage-specific marker genes rather than direct comparison with reference genomes. By using a broad set of such lineage-specific marker genes, CheckM can reliably determine the completeness and contamination levels of MAGs without requiring matched reference genomes. This method is robust and effective for quality assessment in diverse microbial communities. Please refer to the original manuscript for further information (*[*https://doi.org/10.1101%2Fgr.186072.114*](https://doi.org/10.1101%2Fgr.186072.114)*). We added a short description of how checkM can calculate in a reliable way the quality of the MAGs even without a reference genome updated version of the manuscript, page 30, lines 644-646:*

“Estimation of MAG completeness and contamination through CheckM is possible even without matched reference genomes as this tool uses a broad set of lineage-specific marker genes obtained through the reconstruction of a reference genome tree.”

**18.** Line 170: The results of this section and the relevant discussion need to be rephrased to more directly answer the question proposed by the section title.

*We thank the reviewer for this comment. Both the title of this section and the content were modified in the updated version of the manuscript, page 9, lines 209-234:*

“2.2 ~~How model reconstruction can benefit from a “pan” perspective~~ Role of genome completeness in model reconstruction: the foundation for a “pan” perspective

To define a baseline for pan-GEM reconstruction, we first analyzed the relationship between MAG completeness and draft GEM quality in the two datasets. The selected MAGs covered a wide spectrum of completeness in both environments, ranging from 50 to 100%~~.~~ and the maximal ~~MAG~~ completeness of MAGs for individual SGBs without cultured representatives also varied widely. ~~also displayed a wide distribution when considering those without a cultured representative,~~ In several cases, these MAGs did not reach~~ing~~ 90% of completeness ~~in several cases~~

[…]

Traditional de novo draft GEM reconstruction is expected to yield highly gapped models in these cases ~~(Table 1)~~. To assess the extent of these gaps ~~test whether this was the case~~, we computed the correlation between the MAG completeness ~~level of the MAGs~~ and the quality of the reconstructed GEMs. The ~~quality of the models~~ latter was quantified by ~~the similarity~~ comparing the presence and absence of reactions shared between GEMs reconstructed from individual MAGs (MAG-GEMs) and GEMs of reference genomes (iso-GEMs). This similarity was expressed as Matthews correlation coefficient (MCC) ~~F1 score~~. By fitting a generalized additive model to the data (Fig. 2c, 2d) a strong and statistically significant positive correlation emerged (Spearman correlation, *rho*=0.78, P-value <2.2e-16) between the genome completeness and the MCC ~~F1 score~~, confirming the need of a method to overcome current completeness limits. Interestingly, significant ~~differences~~ variability in ~~the~~ this relationship ~~between model quality and genome completeness were~~ was also identified across bacterial phyla (Additional File 1: Fig. S2). Specifically, Bacteroidota and Firmicutes A, the most abundant phyla in the human gut, exhibited the lowest correlation between genome completeness and model quality (Fig. 2c).”

**19.** Line 176: Report the percentage of SGBs that fall below 90% completeness.

*We thank the reviewer for requesting clarification. The percentages of SGBs with more than 30 MAGs and completeness below 90% are 0.6% and 0.7% of the total, and 7.46% and 46.82% of the SGBs without reference genomes for the UHGG and OMD databases, respectively. Despite the number of species falling into this category is low, it is important to note that they are common in the analyzed ecosystems, therefore confirming their key functional role. Their high frequency is confirmed by the fact that more than 30 MAGs have been identified and used for generating accurate pan-GEMs. As suggested, these percentages have been added in the updated version of the manuscript, page 10, lines 218-220:*

“Specifically, OMD had 59 SGBs (0.7% of the total) with maximal MAG completeness levels below 90%, whereas UHGG had ~~38~~ 28 (0.6% of the total - Table 1).”

**20.** Lines 177-178: Table 1 does not show information regarding gaps or reactions. Also, this is only true if the missing genes in MAGs tend to be metabolic genes included in GEMs.

*We thank the reviewer for the comment. Indeed, Table 1 was incorrectly cited and has been moved to the previous sentence. Moreover, it is true that the missing genes will affect the GEMs' quality only if they are directly involved in the metabolism. Nevertheless, Fig. 2 shows an improvement trend even for MAGs with completeness levels between 90% and 100%. Therefore, it is reasonable to assume that missing genes in all MAGs with a completeness level below 90% are likely to include metabolic reactions. The sentence was corrected in the updated version of the manuscript, page 10, lines 218-221 as follows:*

“Specifically, OMD had 59 SGBs (0.7% of the total) with maximal MAG completeness levels below 90%, whereas UHGG had ~~38~~ 28 (0.6% of the total - Table 1). Traditional *de novo* draft GEM reconstruction is expected to yield highly gapped models in these cases ~~(Table 1)~~.”

**21.** Line 181: It would be helpful to quickly mention here that the F1 score is being calculated on reaction presence/absence.

*Done as suggested. The sentence was corrected in the updated version of the manuscript, page 10, lines 223-226 as follows:*

“The ~~quality of the models~~ latter was quantified by ~~the similarity~~ comparing the presence and absence of reactions shared between GEMs reconstructed from individual MAGs (MAG-GEMs) and GEMs of reference genomes (iso-GEMs). This similarity was expressed as Matthews correlation coefficient (MCC) ~~F1 score~~.”

**22.** Line 182: The Methods section does not mention how the additive model for statistical purposes was performed.

*We thank the reviewer for the comment. Additional explanation has been provided in “5.4 Statistical analysis and model quality quantification” in the updated version of the manuscript, pages 34-35, lines 744-747 as follows:*

“Additionally, a generalised additive model fitted to the data to highlight the correlation between GEM quality and MAG completeness (Fig. 2c, d) This was implemented using the “stat_smooth()” function from the “ggplot2” library, with parameters method = 'mgcv::gam' and formula = y ~ s(x, bs = "cs").”

**23.** Line 186: What is "Firmicutes A"?

*Suffixes like “A” are applied to groups of species that, while not appearing monophyletic in the GTDB reference tree, have other evidence suggesting they might actually be monophyletic. The suffixes are also used by the GTDB tool for groups whose placement tends to change or be unstable between different releases of the database. This system helps researchers to understand the current state of knowledge and potential uncertainties in the classification of these groups. The “Firmicutes” phylum is part of the first category, and “A” and “C” were used in the text to distinguish species falling in these two taxa. We clarified this in the updated version of Methods section on page 30, lines 640-644 as follows:*

“The taxonomic classification includes suffixes added according to the GTDB nomenclature to indicate groups of species that, while not appearing monophyletic in the GTDB reference tree, have other evidence suggesting they might actually be monophyletic, as well as groups whose placement tends to change or be unstable between different releases of the database.”

**24.** Lines 274-5: It would help clarify the goals to specify at this point what you mean by "fermentation capabilities," E.G. if you mean the amount produced, the set of products that can be made, etc.

*Modified as suggested, the sentence was corrected in the updated version of the manuscript on page 19, lines 392-399 as follows:*

“Next, we sought to test whether the obtained *pan*-GEMs could more accurately reproduce fermentation capabilities of gut microbial species compared to traditional MAG-~~based~~ and iso-GEMs. The fermentative potential for a given compound was defined as the capacity of a species to secrete that compound over a pre-defined flux threshold normalized over its growth rate (details in the Methods section). To assess this, a ~~A~~ comparative ~~assessment~~ analysis of the predictive accuracy of *in-silico* simulations was conducted for eight common anaerobic fermentation products using metabolic model reconstructions for ~~up to 20~~ 41 different species of the UHGG catalog (Fig. ~~5~~6).”

**25.** Lines 287-290: It is unclear how this accuracy calculation is being done, especially for the FVA. Is a product considered a model positive if it is produced anywhere in the FVA results? Or if it is always produced? Was the FVA performed at or near the maximum growth rate?

*We clarified this aspect in the text as requested. The FVA analysis was performed at the maximum growth rate. Moreover, a GEM was considered as able to produce a by-product if the maximisation of its export flux was greater than 1e-4 mmol∗gDW^−1^, and not merely if the compound export was positive when it was not the focus of the objective function. To improve clarity the updated version of the manuscript was modified, pages 19 and 37 as follows, lines 402-413:*

“The selected metabolites include common anaerobic fermentation end-products, such as acetate, butyrate, lactate, and ethanol. The evaluation involved quantifying the percentage of correctly and incorrectly estimated end-products based on the total number of predictions performed for all the models of a species (Additional File 2: Table S5). Specifically, the confusion matrix per SGB was determined by summing up all the predictions belonging to the same category (e.g. true positive - TP, true negative - TN, false positive - FP, or false negative - FN). Afterwards, the average percentage of TP and TN metabolites across species were estimated through the normalisation of the confusion matrix of each SGB by the total number of tests in that group (i.e. = num. GEMs * num. tested compounds; Additional file 2: Table S6). ~~Moreover, a~~Also in this case, to investigate the influence of the MAG completeness level on the prediction performance, the species-level models were divided into five sets with increasing source MAG quality.”

*5.7 Validation of fermentation product predictions, lines 800-804:*

“[...] actual by-products. Species were considered metabolically capable of secreting a specific compound if positive values were obtained when maximizing the export flux during FVA performed at the maximum growth rate. We set a minimum threshold to include in the fermentation end-product spectrum of the model only metabolites with normalized exchange fluxes (measure in mmol∗gDW^−1^) higher than 1e-4.”

**26.** Lines 274-301: The authors must show TP and TN values, rather than only specifying the trends observed (e.g. minimum, maximum, median, etc).

*We thank the reviewer for the comment. Fig. 6 currently shows the percentage of TP and TN values for each species (grey symbols). In the plot, it is possible to appreciate the percentage of predictions per class (TP, TN, FP, and FN) over all the performed predictions for the MAGs of a species. We believe that a direct representation of a confusion matrix would not be informative due to the significant disparity in the number of MAGs, and thus the number of predictions, between species. To make the results more accessible, the raw simulation results were added to the Supplementary Information (Additional file 2: Table S6).*

**27.** Lines 313-314: How was this 14.6% alignment rate calculated?

*The percentage mentioned by the reviewer refers to the analysis implemented in the work of* [*Nayfach et al., 2021 (*](https://www.zotero.org/google-docs/?broken=KbLGjH)[*https://doi.org/10.1038/s41587-020-0718-6*](https://doi.org/10.1038/s41587-020-0718-6)[*).*](https://www.zotero.org/google-docs/?broken=VoJXku) *Specifically, the authors of the paper reported the following method in the paragraph “Metagenomic read recruitment to MAGs and reference genomes”:*

“3,170 metagenomic samples with available sequencing reads were retrieved from the Joint Genome Institute and Sequence Read Archive databases to quantify mappability (Supplementary Table [4](https://www.nature.com/articles/s41587-020-0718-6#MOESM3)). Up to 500,000 reads from each metagenome were aligned to a database containing 52,515 MAGs and another database containing 151,730 genomes obtained from NCBI RefSeq (release 93).”

*For clarity, the reference was also added to the corresponding sentence. Page 23, lines 463-466:*

“Here, on average only ~14.6% of reads per sample ~~were~~ could be aligned to ~~isolate genomes~~ the references [27].~~, indicating that~~ Thus, *in -silico* metabolic network analysis of most microbial species, just like genomic analysis, has to rely on MAGs, with the associated risks of potentially missing relevant functional components.”

**28.** Lines 318-320: Using a MRF of 7% to reconstruct species-level GEMs seems to contradict the goal to "assess which reactions are persistent constituents of the metabolism of a taxon" - a reaction being present in only 7% of genomes does not seem to be part of the core set.

*We thank the reviewer for the comment. We agree that the described goals were too stringent and do not reflect the scope of a tool aiming to reconstruct functional metabolic models. Indeed, including in the pan-GEMs only the reaction persistently present in all the GEMs of a species would exclude a large fraction of the species metabolism (see consideration on pan-reactome structure commented in the answer to the major comments n.1 of the first reviewer). The target of the tool was better clarified in the updated version of the manuscript as follows:*

*Page 23, lines 470-473:*

“The novelty of pan-Draft lies in the combination of a homology based approach, predicting the metabolic potential of a genome, with a pan-reactome analysis~~,~~ assessing which reactions are ~~persistent~~ common constituents of the metabolism of a taxon.”

*Page 5, lines 132-134:*

“~~The~~ Cross-comparison of draft models belonging to ~~the~~ a same taxon facilitates the reconstruction of a ~~solid “core”~~ network representative of the majority of the metabolic variability within the cluster.”

*Page 11, lines 247-250:*

“Moreover, correctly predicted reactions (i.e. those actually belonging to ~~the~~ a species pan-reactome) that are present only within small sub-populations ~~are~~ should be preferentially filtered out to generate a draft representative of ~~the~~ a species ~~core~~ metabolism ~~of a species~~.”

*Page 32, lines 684-686:*

“The aim of this step is to assess which reactions share higher frequency among the models in order to identify the central ~~core~~ metabolism of a species and exclude the rarest functions.”

**29.** Line 400: Change to "low rank taxonomy" or "species level."

*Done as suggested, page 27, lines 557-559:*

“Moreover, while pan-Draft has been introduced for reconstructing species-level models, in principle its ~~application~~ applicability is not limited to low ~~taxonomies~~ rank taxonomy.”

**30.** Line 445: What is "solidity" in this context?

*The sentence was modified for clarity, page 28, lines 603-605:*

“Finally, future studies should explore the impact of contamination on the ~~solidity~~ quality of species-level models, specifically testing ~~the~~ its influence on ~~selecting~~ the MRF threshold ~~for reactions inclusion in the model~~.”

**31.** Line 599: Change to 1e-4 without an exponent. Also correct superscript format in "mmol∗gDW−1"

*Done as suggested, page 37, lines 802-804:*

“We set a minimum threshold to include in the fermentation end-product spectrum of the model only metabolites with normalized exchange fluxes (measure in mmol∗gDW^−1^) higher than 1e^-4^.”

**32.** Lines 559-567: "Minimum reaction frequency" is not defined.

*Added as suggested, page 35, lines 749-753:*

“The MRF is the minimum occurrence in the genome pool, expressed in percentage, necessary for a reaction to be included in the *pan*-GEM. This frequency is calculated as the ratio of the number of MAG-GEMs containing the reaction to the total number of MAG-GEMs in that SGB. To determine the MRF necessary for a reaction to […]”.

**33.** Line 560: Correct "To determine theMRF necessary."

*Done as suggested. See the previous comment.*

**34.** Line 568: Consider rephrasing this header to something that more specifically states what is being found, E.G. "assessing MAGs completeness in quality of species-level GEMs."

*Done as suggested, page 36, line 767:*

“5.6. ~~Test improvement potential of single strain to species-level model~~ Quantify structural improvement of species-level models with respect to MAGs completeness”

Figures

**1.** Figure 1: It is unclear what is meant by the red box around reaction prediction, pathway prediction, and draft network reconstruction.

*We thank the reviewer for the comment. The red box highlights which information (e.g. files) pan-Draft needs to reconstruct a draft pan-GEM. It shows which output of the gapseq pipeline the pan-Draft module integrates within the GEM reconstruction. The caption of the figure was modified to improve clarity.*

“**Fig. 1. Flowchart showing the dataset filtering steps and the pan-Draft workflow.** Set of filtering criteria implemented on the UHGG and OMD dataset to select SGBs with non-redundant genomic information for pan-Draft validation. A minimum threshold of 30 MAGs was imposed to reconstruct a set of draft models through *gapseq*. *pan-Draft* is integrated within the reconstruction pipeline by processing the draft models and the ~~correlated~~ genetic evidence of reactions ~~(highlighted in red) to generate a pan-GEM~~. The information used for each genome of a SGB to generate the *pan*-GEM is highlighted by the red box.”

2. Fig 4b: The F1 score difference legend should be placed next to the heatmap. Currently it appears to be part of 4a.

*We thank the reviewer for the comment. The legend of the heatmap (b) was placed on the left of the image for space optimization of the multipanel. We believe that the fact that red gradients are not present in the box plot (5a) excludes misinterpretation of the conveyed information. We slightly rearranged the single elements and we think that the updated version of Fig.5 shows a better displacement of the legends. Also, due to the increased number of species analysed only SGB with more than 30 MAGs in each completeness subset were depicted in Fig.5. The remaining SGBs have been added in Fig. S4. of the supplementary material.*

**Fig. ~~4~~5. Structural quality improvement obtained for the *pan*-GEMs in comparison to the MAG models.** Graphs showing the changes in quality between the *pan*-GEM and MAGs’ models in relation to the reference reaction set. Global results are reported for all SGBs except *E. coli* (**a**) and for single species (**b**). The heatmap reports explicitly the average difference between the MCC ~~F1 score~~ of each species *pan*-GEM and of the GEM from its most complete MAG. Species-level model improvements were analyzed based on MAG completeness. Completeness level values highlight the thresholds used to subset the MAGs into five groups. Only SGBs with more than 30 MAGs in each subset were depicted ~~Gray boxes refer to missing~~ *~~pan~~*~~-GEMs occurring when the number of available MAGs in the subsets were below 30~~. All statistics are calculated on gapfilled models.

**Fig. S4. Enhancement in structural quality of *pan*-GEMs compared to MAG models.** The heatmap shows the average difference in MCC between each species' *pan*-GEM and the GEM from its most complete MAG. The analysis was conducted at the species level, with MAGs grouped based on their completeness. Threshold values indicate the categories used to partition the MAGs into five groups. Gray boxes denote missing *pan*-GEMs, a condition occurring when the subset contains fewer than 30 available MAGs. All statistics are based on gapfilled models.

**2^nd^ round**

**Reviewer 1**

I thank the authors for their answers and for their solid work in responding to all my comments with arguments and additions:
- more description of the results, with new paragraphs and addition of definition in supplementary and the exploration of the impact of the number of MAGs.
- a new comparison with MIGRENE and the addition of figures on the functional classification of pan-reactome.
- the added experiments on well-known species. The results for Escherichia coli, although different from those for the other species, are consistent with the concept of an open pangenome for this species (such has indicated in this article https://bmcbiol.biomedcentral.com/articles/10.1186/s12915-022-01347-7).
- the addition of iso-GEMs in Figure 5.
- the addition of the runtimes of gapseq and pan-Draft in supplementary file.
- the consistent use of the MCC instead of the F-measure as proposed by reviewer 2.

\# Software review

I thank the authors for taking care of my comments and updating gapseq code.
I used gapseq and gapseq pan-Draft from the Zenodo archive at the species-level (‘Aliivibrio fischeri’) and with genomes only defined by their genus name (‘Qipengyuania sp.’) of the Marine v2.0 dataset of MGnify (https://ftp.ebi.ac.uk/pub/databases/metagenomics/mgnify_genomes/marine/v2.0/).
Thanks to the changes made by the authors, using 'gapseq doall' then 'gapseq pan' worked without any problems.
I found similar results than the ones of the article and in my first round of review. But with the genomes associated with genus Qipengyuania, I had a difference in the number of reactions with far more reactions in the pan-GEMs compared to the isolate or the MAGs (2,428 vs 1,092 to 1,946 reactions). Furthermore, the distribution of reactions were different in the shell between this genus-level and the species-level (1,633 reactions in shell compared to 302 reactions for species-level ‘Aliivibrio fischeri’). This is expected as there is more diversity inside a genus than inside a species.
When working at the species-level, there are already more reactions in the pan-GEMs than in the MAG-GEMs or the iso-GEMs, but I fear that when working at the genus level the number of reactions added could lead to problems similar to those identified in the Escherichia coli analysis. And a possible solution could be the one suggested by the authors for Escherichia coli: dividing the genomes into a priori groups according to their genetic similarities.

\# Minor comments:

- Lines 496-497: as there are no experiments that demonstrate the use of pan-Draft at the genus-level in the article and with the limited experiment presented in the previous paragraph, a conditional sentence could be more in line with what is shown in the article.
The sentence is affirmative on the use of pan-Draft at the genus-level and implies that results could be directly analysed. But I think that several issues could come from the use of this method on higher taxonomic level with, for example, pan-GEMs with too many reactions that could pose questions on the representativeness of these GEMs.
I agree that pan-Draft could be interesting to use at the genus level, but I think that it might require work both before and after pan-Draft to ensure the representativeness and usefulness of the reconstructed pan-GEMs. That's why my suggestion to the authors is to use a more conditional sentence.

- line 43: just to point an heterogeneity, in case if it is a mistake, CarveMe, gapseq and ModelSEED are in italics whereas RAVEN, Merlin and AuReMe are not.

- line 127, “draft and find-transport pan-Draft aims to”, adding a separator could ease the reading of the sentence, such as: “draft and find-transport commands of gapseq, pan-Draft aims to”.

- Figure 4: if I am not mistaken, panels (a), (b) and (c) do not show the number of reactions/metabolites, but the ratio between the number of reactions/metabolites in the pan-GEMs compared to those in the iso-GEMs. The term 'number' could therefore be replaced by 'ratio' in the y-axis labels and captions for these panels.

- Figure 5: in panel (b), there is a missing ‘l’ in the label of the y-axis for ‘Completeness’.

- Line 660: ‘-all-Reactions.tbl.gz’, I think that the ‘.gz’ can be removed due to the modification of the software.

- line 676: missing citation of ggplot2.

- line 691: ‘form batches of 2, 5, 10, 15, 30, 60, 100, and 1000 sequences, iterating ten times for each SGB’, I think that the term sequences can be misinterpreted here (as nucleotides/protein sequences of the MAGs), could it be replaced by ‘MAGs’?

- line 718-719: missing citations of R, sybil and cplex.

- In Table S3 of Additional file 2: typo in line 2, missing ‘h’ in characterization.

I thank the authors again for their work and I recommend this article for publication as the method presented is well described and shows its usefulness and performance.

**Author’s response**

Minor Comments

- Lines 496-497: as there are no experiments that demonstrate the use of pan-Draft at the genus-level in the article and with the limited experiment presented in the previous paragraph, a conditional sentence could be more in line with what is shown in the article.

The sentence is affirmative on the use of pan-Draft at the genus-level and implies that results could be directly analysed. But I think that several issues could come from the use of this method on higher taxonomic level with, for example, pan-GEMs with too many reactions that could pose questions on the representativeness of these GEMs. I agree that pan-Draft could be interesting to use at the genus level, but I think that it might require work both before and after pan-Draft to ensure the representativeness and usefulness of the reconstructed pan-GEMs. That's why my suggestion to the authors is to use a more conditional sentence.

We appreciate the reviewer's insightful comment and the concerns raised regarding the application of pan-Draft at the genus-level. We agree that further experimentation and validation are necessary to fully understand the implications of using pan-Draft across higher taxonomic levels. In response, we have revised the statement to reflect a more conditional tone.

“Moreover, while pan-Draft has been introduced for reconstructing species-level models, in principle its applicability is may not be limited to low rank taxonomy. For instance, it could can be extended to the genus level by simply modifying properly curating the selection of input MAGs.”

- line 43: just to point an heterogeneity, in case if it is a mistake, CarveMe, gapseq and ModelSEED are in italics whereas RAVEN, Merlin and AuReMe are not.

We thank the reviewer for the comment. The style was corrected.

The approach adopted for GEM reconstruction by some of the most successful tools, such as CarveMe, gapseq, RAVEN, Merlin, AuReMe, and ModelSEED, is based on sequence homology search and the definition of gene-protein-reaction rules [6–11].

- line 127, “draft and find-transport pan-Draft aims to”, adding a separator could ease the reading of the sentence, such as: “draft and find-transport commands of gapseq, pan-Draft aims to”.

We thank the reviewer for the comment. The sentence was modified as suggested. “By processing the output of the find, draft and find-transport commands, pan-Draft aims to reconstruct high-quality draft metabolic models from several incomplete MAGs having a potentially complementary gene content through a comparative pan-reactome analysis.”

- Figure 4: if I am not mistaken, panels (a), (b) and (c) do not show the number of reactions/metabolites, but the ratio between the number of reactions/metabolites in the pan-GEMs compared to those in the iso-GEMs. The term 'number' could therefore be replaced by 'ratio' in the y-axis labels and captions for these panels.

We thank the reviewer for the comment. The figure and caption were modified as follows.

Fig. 4 Structural and metabolic features of pan-GEMs. The upper boxplots summarize the ratios of the number of metabolites (a), the number of reactions (b), and the number of reactions with associated genetic information (c) in of MAG-GEMs and pan- GEMs, relative to the corresponding normalized by the median values of iso-GEMs. Red dots indicate the statistics for the pan-GEMs reconstructed with all the MAGs of an SGB, either in the UHGG (red boxes) and OMD dataset (light blue boxes). The lower boxplot shows the functional classification of reactions grouped into pan-reactome categories by MetaCyc pathways ontology (d). Core, shell, and cloud consist of all reactions with a frequency higher than 95%, between 5% and 95%, and below 5%, respectively, in all the GEMs of any SGB.

- Figure 5: in panel (b), there is a missing ‘l’ in the label of the y-axis for ‘Completeness’.

We thank the reviewer for noticing the error. The label was modified as suggested.

- Line 660: ‘-all-Reactions.tbl.gz’, I think that the ‘.gz’ can be removed due to the modification of the software.

Modified as suggested.

“This was done by parsing the "dbhit" column of the “-all-Reactions.tbl.gz” table which identifies pathway predictions in bacterial species mapping to the gapseq reaction database.”

- line 676: missing citation of ggplot2.

The citation was added as suggested.

“This was implemented using the “stat_smooth()” function from the “ggplot2” library, with parameters method = 'mgcv::gam' and formula = y ~ s(x, bs = "cs") [59].”

The “References” section was modified as follow:

“59. Wickham H. ggplot2: Elegant Graphics for Data Analysis. Springer-Verlag New York 2016; ISBN 978-3-319-24277-4, https://ggplot2.tidyverse.org.”

- line 691: ‘form batches of 2, 5, 10, 15, 30, 60, 100, and 1000 sequences, iterating ten times for each SGB’, I think that the term sequences can be misinterpreted here (as nucleotides/protein sequences of the MAGs), could it be replaced by ‘MAGs’?

We appreciate the reviewer's for the required clarification. The text was modified as suggested.

“Additionally, to validate whether the identified threshold is reliable using a varying number of genomes, MAGs each SGB was were randomly sampled to form batches of 2, 5, 10, 15, 30, 60, 100, and 1000 sequences MAGs, with each sampling process repeated iterating ten times for each SGB.”

- line 718-719: missing citations of R, sybil and cplex.

The citations were added as suggested.

“All the simulations were performed in R (v.4.3.1) using sybil (v.2.2.0) and CPLEX solver (v.22.11) [62-64].”

The “References” section was modified as follow:

62. R Core Team (2024). R: A language and environment for statistical computing. R

Foundation for Statistical Computing, Vienna, Austria. https://www.R-project.org/.

63. Gelius-Dietrich G, Desouki AA, Fritzemeier CJ, Lercher MJ. sybil – Efficient constraint-based modelling in R. BMC Syst Biol. 2013;7:125.

64. Cplex, I. I. (2009). V12. 1: User’s Manual for CPLEX. International Business Machines Corporation. https://www.ibm.com/products/ilog-cplex-optimization-studio

- In Table S3 of Additional file 2: typo in line 2, missing ‘h’ in characterization.

We appreciate the reviewer’s correction.

“* SGBs with characterization of the fermentation products but less than 30 MAGs in the completeness level subset to generate a pan-GEMs”
